# Modeling native and seeded Synuclein aggregation and related cellular dysfunctions in dopaminergic neurons derived by a new set of isogenic iPSC lines with SNCA multiplications

**DOI:** 10.1038/s41419-022-05330-6

**Published:** 2022-10-19

**Authors:** Angelo Iannielli, Mirko Luoni, Serena Gea Giannelli, Rosangela Ferese, Gabriele Ordazzo, Matteo Fossati, Andrea Raimondi, Felipe Opazo, Olga Corti, Jochen H. M. Prehn, Stefano Gambardella, Ronald Melki, Vania Broccoli

**Affiliations:** 1 National Research Council (CNR), Institute of Neuroscience, 20129 Milan, Italy; 2 Division of Neuroscience, San Raffaele Scientific Institute, 20132 Milan, Italy; 3 IRCCS Neuromed, Pozzilli, Italy; 4 IRCCS Humanitas Research Hospital, via Manzoni 56, 20089 Rozzano Milan, Italy; 5 Experimental Imaging Center, San Raffaele Scientific Institute, Milan, Italy; 6 University Medical Center Göttingen, D-37073 Göttingen, Germany; 7 Sorbonne Université, Institut du Cerveau (ICM), Inserm U1127, CNRS, UMR 7225 Paris, France; 8 Royal College of Surgeons in Ireland University of Medicine and Health Sciences, Department of Physiology and Medical Physics and SFI FutureNeuro Research Centre, 123 St. Stephen’s Green, Dublin, Ireland; 9 Department of Biomolecular Sciences, University of Urbino “Carlo Bo,, Urbino, Italy; 10 Institut Francois Jacob, Molecular Imaging Center (MIRCen), Commissariat à l’Energie Atomique et aux Energies Alternatives (CEA) and Centre National de la Recherche Scientifique (CNRS), Université Paris-Saclay, Fontenay-aux-Roses, France

**Keywords:** Parkinson's disease, Neurodegeneration

## Abstract

Triplication of the *SNCA* gene, encoding the protein alpha-Synuclein (αSyn), is a rare cause of aggressive and early-onset parkinsonism. Herein, we generated iPSCs from two siblings with a recently described compact *SNCA* gene triplication and suffering from severe motor impairments, psychiatric symptoms, and cognitive deterioration. Using CRISPR/Cas9 gene editing, each *SNCA* copy was inactivated by targeted indel mutations generating a panel of isogenic iPSCs with a decremental number from 4 down to none of functional *SNCA* gene alleles. We differentiated these iPSC lines in midbrain dopaminergic (DA) neuronal cultures to characterize αSyn aggregation in native and seeded conditions and evaluate its associated cellular dysfunctions. Utilizing a new nanobody-based biosensor combined with super-resolved imaging, we were able to visualize and measure αSyn aggregates in early DA neurons in unstimulated conditions. Calcium dysregulation and mitochondrial alterations were the first pathological signs detectable in early differentiated DA neuronal cultures. Accelerated αSyn aggregation was induced by exposing neurons to structurally well-characterized synthetic αSyn fibrils. 4x*SNCA* DA neurons showed the highest vulnerability, which was associated with high levels of oxidized DA and amplified by TAX1BP1 gene disruption. Seeded DA neurons developed large αSyn deposits whose morphology and internal constituents resembled Lewy bodies commonly observed in Parkinson’s disease (PD) patient brain tissues. These findings provide strong evidence that this isogenic panel of iPSCs with *SNCA* multiplications offers a remarkable cellular platform to investigate mechanisms of PD and validate candidate inhibitors of native and seeded αSyn aggregation.

## Introduction

Although Parkinson’s disease (PD) is a neurodegenerative disorder which commonly is manifested in idiopathic forms, about 5% of cases are familiar and caused by pathological alterations in single genes [1–3]. The alpha-Synuclein (αSyn) encoding gene *SNCA* was the first genetic locus identified with a missense mutation responsible for a dominant genetic form of PD originally described in the large Contursi kindred [4]. Importantly, αSyn protein aggregates are one of the major constituents of Lewy Bodies, cytoplasmic inclusions are predominantly present in neurons of autopsy-derived PD brain tissues [5]. Subsequent genetic studies identified additional pathogenetic alterations in *SNCA*, including other point mutations and multiplications in the form of duplications and triplications [6–8]. Although *SNCA* duplications are relatively common in inherited dominant PD forms, missense mutations and triplications are extremely rare [9]. Patients with three *SNCA* copies manifest comparable disease onset and progression with those described in idiopathic forms, although they are more likely to develop cognitive decline and sleep disturbance [8]. In contrast, patients with *SNCA* triplication develop aggressive and early-onset parkinsonism, combined with additional non-motor signs, such as cognitive dysfunctions with features reminiscent of dementia with Lewy bodies [9, 10]. These clinical presentations suggest that *SNCA* triplications cause a more severe phenotype than *SNCA* duplications, consistently with the underlying genetic defects. Thus, PD patients with four *SNCA* copies offer a unique opportunity to unveil the underlying pathophysiological mechanisms directly triggered by increased *SNCA* gene dosage. With this in mind, iPSC technology offers a powerful system to generate and characterize patient-specific cells affected by the disease. Recent studies have convincingly reported a variety of pathophysiological defects exhibited by neural cells with *SNCA* triplication, such as increased endoplasmic reticulum stress, altered lysosomal and mitochondrial functions, dysregulated autophagy and heightened oxidative stress levels [11–17]. However, these pathological alterations have been described in neurons after advanced time in culture, while the exact early dysfunctions and chain of pathological events remain to be fully elucidated. In addition, the available iPSCs with *SNCA* triplication have been originally derived by only two patients, and most of the cell lines were reprogrammed by integrating retroviruses [16–18]. Thus, it is imperative to produce additional iPSC lines with non-integrating methodologies from more patients and develop isogenic controls for a more stringent characterization. Moreover, the availability of iPSCs from patients with different clinical presentations might offer new opportunities for identifying disease gene modifiers by candidate analysis or unbiased screens. Recently, an additional pedigree with a dominant inheritance of multiplication of *SNCA* has been reported that was characterized by four copies of *SNCA,* three of which derived from a triplicated region of 351Kb containing *SNCA*, and a duplication of a genomic region flanking the triplication [19, 20]. The two siblings (brother and sister) analyzed in these studies showed rapid motor deterioration, cognitive and psychiatric symptoms, and sleep disturbances. However, the brother showed earlier onset compared to the sister (age 28 and 42, respectively) and significantly faster progression of both motor and non-motor clinical presentations. Thus, although *SNCA* triplication causes a highly penetrant and aggressive PD form, the time and severity of symptom presentations can vary, requiring a deep and longitudinal clinical assessment to classify the diverse phenotypes. Similar symptomatic differences have been extrapolated among PD patients belonging to 8 different families with *SNCA* triplication inheritance [8]. Herein, we generated iPSCs from the two siblings described above and produced a panel of isogenic iPSCs by targeted inactivation of each of the SNCA copies by CRISPR/Cas9 gene editing up to obtaining isogenic SNCA knock-out cells. These iPSCs were differentiated into DA neuronal cultures to characterize native and seeded αSyn aggregation with a new biosensor and correlate αSyn aggregation with cellular dysfunctions and neuronal survival.

## Results

### Generation of a panel of isogenic iPSC lines with a novel SNCA multiplication

**Fig. 1:**
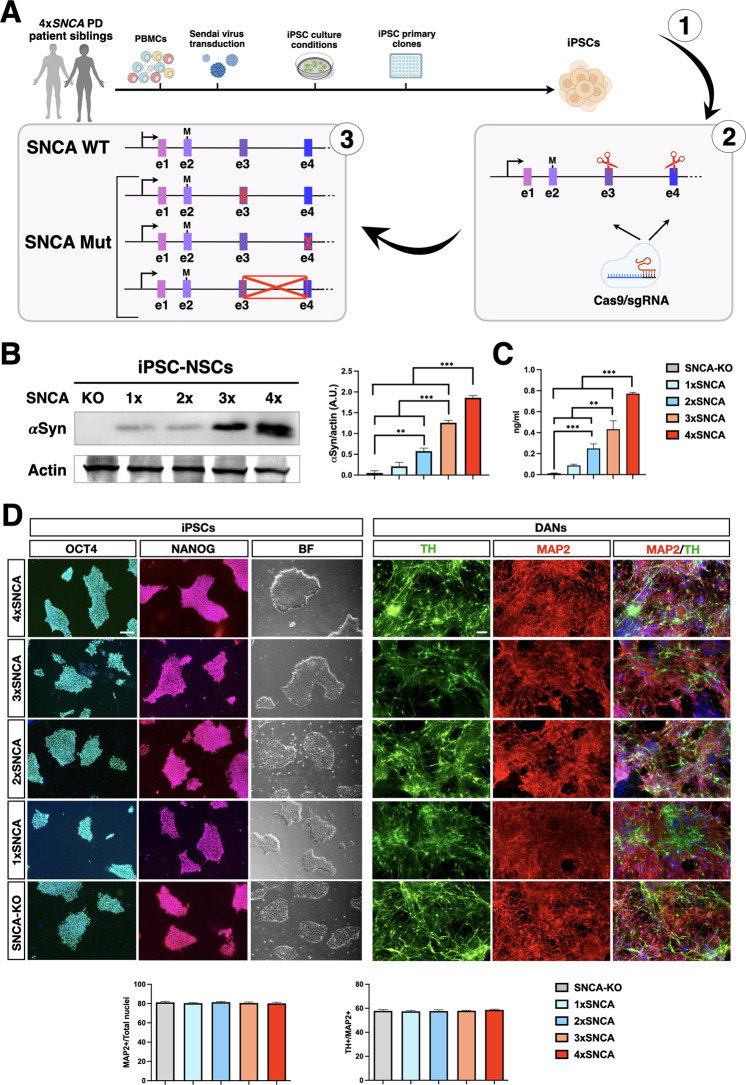
Generation of a set of iPSCs with an incremental number of SNCA alleles by CRISPR/Cas9 gene editing. **A** Schematic representation of the protocol for generating 4x, 3x, 2x, and 1x*SNCA* iPSC lines. **B** Immunoblot analysis and quantification for αSYN protein in 4x, 3x, 2x, and 1x*SNCA* NPCs (*n* = 3 independent experiments). **C** Quantification of αSYN protein levels in cell supernatants (*n* = 3 independent experiments). **D** iPSC colonies were immunostained with the pluripotency markers OCT4 and NANOG and detected in a bright field for morphology appearance assessment. Representative images and quantification for midbrain DA neuronal cultures from 4X, 3X, 2X, and 1X *SNCA* and SNCA-KO iPSCs immunostained with the DA marker TH and neuronal marker MAP2 (three fields, three independent experiments). Values are mean ± SEM of *n* = 3 independent experiments. *p < 0.05; **p < 0.01, ***p < 0.001. Statistical analysis is performed using one-way ANOVA followed by Tukey post-test. Scale bars, 100 µm.

Given the uniqueness of this *SNCA* multiplication and its association with a particularly severe and fast-progressing PD pathological phenotype, we decided to derive iPSC lines through cell reprogramming. To achieve this goal, peripheral blood withdrawals from both siblings were obtained from isolated mononuclear cells (PBMCs) that were reprogrammed into iPSCs with a non-integrating system based on Sendai viruses expressing the Yamanaka’s genes (CytoTune^TM^-iPS). iPSC lines were initially selected based on the correct colony morphology, normal karyotype, expression of crucial pluripotency genes, and silencing of fibroblast-specific genes (Figs. 1A and S1A, B). Then, two independent lines for each of the siblings (brother: iPSC-3.3 A,B and sister: iPSC-3.2 A,B) were assessed for the presence of the *SNCA* triplication that was confirmed through MLPA analysis (Fig. S1C). Next, the four selected iPSC lines were induced into neural progenitor cells (NPCs) following a consolidated in vitro differentiation protocol [21]. While the iPSC-3.2 A,B lines derived from the sister consistently generated highly proliferating and homogeneous NPC cultures, the brother’s iPSC-3.3 A,B lines gave rise to highly unstable NPC cultures with poor differentiation capability (data not shown). These alterations in iPSCs are in accordance with the relatively different severity of symptoms manifested by the two siblings, with the brother displaying a significantly earlier onset and faster disease progression with respect to the affected sister [19]. Therefore, these results indicate that unknown genetic modifiers can substantially modulate the outcome of the disease phenotype despite the high penetrance of the genetic *SNCA* triplication. Despite our interest in this aspect, we decided to focus this study exclusively on the analysis of the sister’s iPSC-3.2 lines and their derivatives. In order to generate isogenic controls, we employed CRISPR/Cas9 technology to inactivate each of the four *SNCA* alleles in the 4x*SNCA* iPSC-3.2 A line (henceforth cited only as 4x*SNCA*). To do so, we validated one sgRNA with an efficient and selective targeting of the *SNCA* exon 3 encoding for the N-terminal amphipathic region (Fig. 1A). After simultaneous transfection of sgRNA-Ex3 and spCas9 expressing plasmids and transient selection with antibiotics, we isolated and screened primary clones for the desired modifications. Through TIDE analysis of the PCR product combined with Sanger sequencing of at least 40 amplicons, we selected iPSCs lines where either one, two, or three *SNCA* alleles were targeted with frameshift-inducing indel mutations, generating the 3x, 2x, and 1x*SNCA* iPSC lines, respectively (Figs. 1A and S2). However, we were unable to obtain a clone with all four *SNCA* alleles inactivated. Thus, we designed a second sgRNA on *SNCA* exon 4 and repeated the CRISPR/Cas9 gene editing with the same 4x*SNCA* line combining this time the two exon 3 and 4 sgRNAs. By genomic PCR amplification, we selected iPSC clones with an evident deletion between *SNCA* exons 4 and 3, followed by TIDE analysis and amplicon sequencing to detect indel mutations in either exon. After this analysis, we were able to isolate *SNCA*-KO iPSCs where all four *SNCA* alleles were either deleted or inactivated by indel mutations (Fig. S2). To confirm the functional effects of the Cas9-induced indel mutations, we analyzed αSyn protein levels in NPCs, since undifferentiated iPSCs have negligible levels of αSyn expression. Intriguingly, the total amount of αSyn protein was strictly correlated to the number of unmodified *SNCA* alleles, with the patient 4x*SNCA* NPCs having the highest abundance while the SNCA-KO NPCs showed no detectable trace of αSyn protein (Fig. 1B). These results were corroborated by immunohistochemistry on iPSC-derived neurons where the intensity of the immunostaining signal between the different lines was correlated to the number of unmodified and functional *SNCA* alleles (Fig. S1D). In addition, analysis of extracellular αSyn secreted into the culture medium by ELISA assay revealed marked increased αSyn release in 4x*SNCA* NPCs (Fig. 1C). Thus, we selected two iPSC lines each for 4x*SNCA* (parental and unmodified iPSC-3.2 A lines), 2x*SNCA* (clones #22, #29) and *SNCA*-KO (clones #40, #51) that showed stable clone morphology and growth, normal karyotype, high expression of the pluripotent genes and highly efficient neuronal differentiation for the subsequent functional analysis (Figs. 1D and S1A).

### Cytoplasmic αSyn assemblies in iPSC-derived DA neuronal cultures in basal conditions

**Fig. 2:**
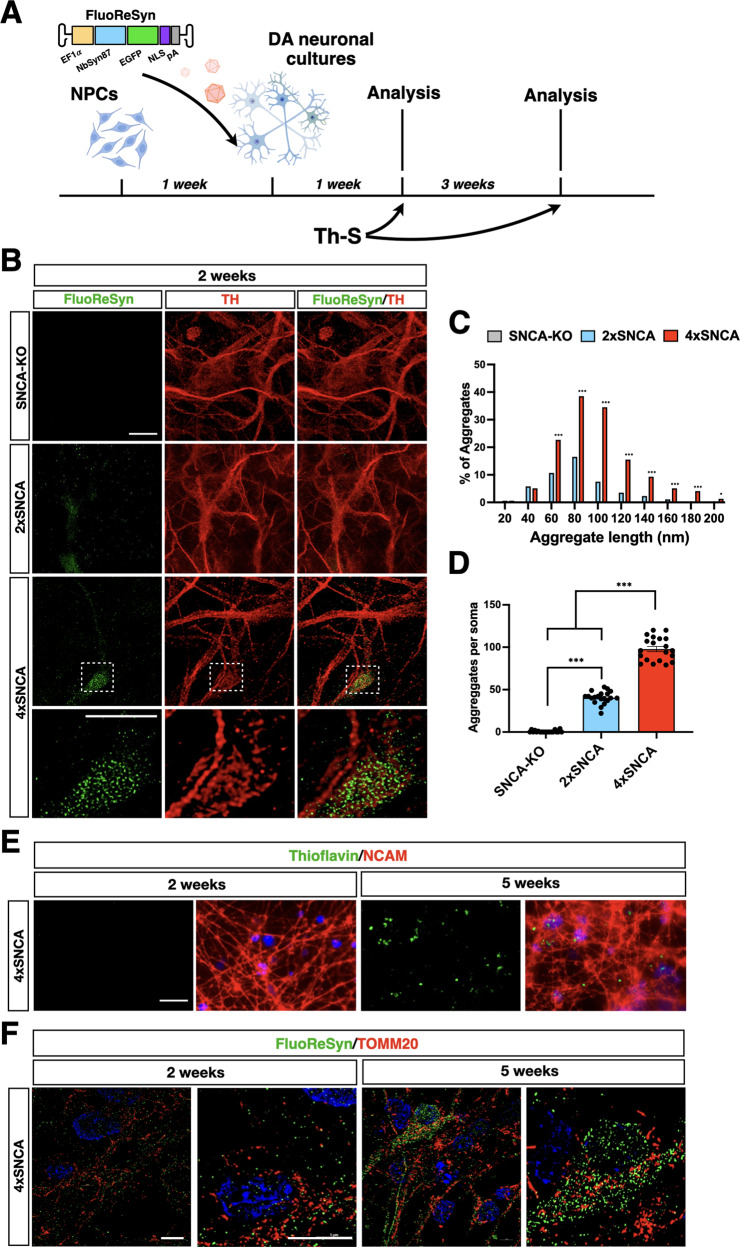
FluoReSyn imaging enables the early detection of endogenous αSyn aggregates in DA neurons. **A** Schematic view of the protocol to infect DA neuronal cultures with AAV-FluoReSyn. **B** Immunostaining for FluoReSyn shows high-level αSyn assemblies in 4x*SNCA* compared to isogenic control 2x*SNCA* DA neurons. **C**, **D** Quantification of the number of αSyn aggregates (**C**) and their size (**D**) in 2x and 4x*SNCA* DA neurons. Dots indicate quantifications in five different fields for four independent experiments. **E** Representative images of Thioflavin S signal in 4x*SNCA* DA neurons. **F** Images of super-resolved imaging show overlapping between αSyn puncta and mitochondrial structures in 2 and 4 weeks old 4x*SNCA* DA neurons. Values are mean ± SEM. *p < 0.05, **p < 0.01, ***p < 0.001. Statistical analysis is performed using one-way ANOVA followed by Tukey post-test. Scale bars, 100 µm.

Given the general high abundance of αSyn protein in 4x*SNCA* iPSCs, we wondered whether these cells would be prone to develop signs of αSyn aggregation in unstimulated conditions. Hence, iPSCs were induced into midbrain DA neurons following a standard neural midbrain floor-plate protocol which included the generation of homogeneous cultures of DA neural progenitors as an intermediate step during the cell differentiation process (Fig. S3). All iPSC lines showed a very high neuronal differentiation efficiency with a large fraction of MAP2^+^ neurons expressing Tyrosine Hydroxylase (TH) (4x*SNCA*: 67% TH^+^/MAP2^+^; 2x*SNCA*: 71% TH^+^/MAP2^+^; *SNCA*-KO: 74% TH^+^/MAP2^+^) (Figs. 1 and S3). To visualize αSyn oligomers in early generated neurons, we decided to combine fluorescent reporter staining with super-resolution microscopy. Initially, we generated both lentiviruses (LVs) and adeno-associated viruses (AAVs) expressing a GFP-tagged SNCA that could bind to nascent αSyn assemblies and, thereby, preferentially highlight cytoplasmic aggregates. However, with both viruses, GFP expression appeared very homogeneous throughout the cytoplasm of neurons, failing to discriminate any αSyn aggregates (Fig. S4 and data not shown). Next, we decided to exploit the nanobody-derived fluorescent reporter FluoReSyn recently generated by Gerdes and colleagues [22], which relies on the specific and selective binding of a nanobody to the C-terminal region of αSyn. Interestingly, FluoReSyn has a second binding to the 20S proteasome subunit Rpn10, which stimulates its degradation in the absence of αSyn binding. Thus, FluoReSyn has an excellent background-to-signal ratio representing a valuable biosensor to detect αSyn protein. We reasoned that FluoReSyn could be further exploited to highlight intra-cytoplasmatic αSyn assemblies thanks to its small size, stability, and low background. Thus, we packaged FluoReSyn into an AAV-PHP.B (AAV-FluoReSyn) to transduce 1-week-old iPSC-derived DA neurons before processing them for STED3X and Lattice SIM^2^ super-resolution microscopy with a resolution below 50 nm (Fig. 2A). Intriguingly, the FluoReSyn signal was easily detectable after immunocytochemical staining revealing significantly more positive puncta in 4x*SNCA* when compared to isogenic control 2x*SNCA* DA neurons (Fig. 2B). Importantly, FluoReSyn staining was undetectable in *SNCA*-KO neuronal cultures confirming the specificity and low background of this biosensor (Fig. 2B). Quantitative analysis revealed that 4x*SNCA* neurons developed a more diffuse accumulation of αSyn aggregates per neuronal somata (4x*SNCA*: 94 ± 14; 2x*SNCA:* 37 ± 8; *SNCA*-KO: 4 ± 3), with larger average size compared to isogenic control DA neurons (4x*SNCA*: 112 nm ± 18; 2x*SNCA:* 46 nm ± 9 in average) (Fig. 2C, D). Next, we stained DA neuronal cultures with Thioflavin S, a dye commonly utilized to detect mature protein aggregates of an amyloid nature. Differently from FluoReSyn, Thioflavin S was not sensitive enough to highlight small aggregates in 2 weeks old DA neurons. However, Thioflavin S positive puncta were detectable in 5 weeks old 4x*SNCA* neurons indicating a progressive accumulation of αSyn aggregates during neuronal maturation in vitro (Fig. 2E). Intriguingly, the FluoReSyn signal increased proportionally with Thioflavin S and Serine 129 phosphorylated αSyn (pS129αSYN) staining in neuronal cultures of iPSCs with additional *SNCA* copy number and over time in culture (Figs. S5–S8). Moreover, direct side-by-side staining revealed that pS129αSYN-positive puncta were virtually always stained with the FluoReSyn biosensor (Fig. S9).

Given the well-described interactions between αSyn aggregates and mitochondria, we assessed their physical proximity on 4x*SNCA* neuronal cultures. Remarkably, already in 2 weeks old 4x*SNCA* neurons, about 9% ± 2 of all FluoReSyn puncta were superimposable with TOMM20 positive mitochondrial structures in super-resolved imaging (Fig. 2F). The index of proximity was further increased to 14% ± 4 in 5 weeks old 4x*SNCA* neurons (Fig. 2F). A comparable juxtaposition was revealed using a co-staining between FluoReSyn and ATP5A1 (Fig. S10), a subunit of the ATP-synthase complex which has been previously co-localized with αSyn oligomers [23].

These results reveal that 4x*SNCA* neurons exhibit early aggregate formation in the basal state without the need for any external inducers or stressors, and aggregation dynamics can be profiled and measured using a nanobody-based biosensor coupled with super-resolved imaging.

### Metabolic dysfunctions and calcium dysregulation in 4x*SNCA* DA neurons

**Fig. 3:**
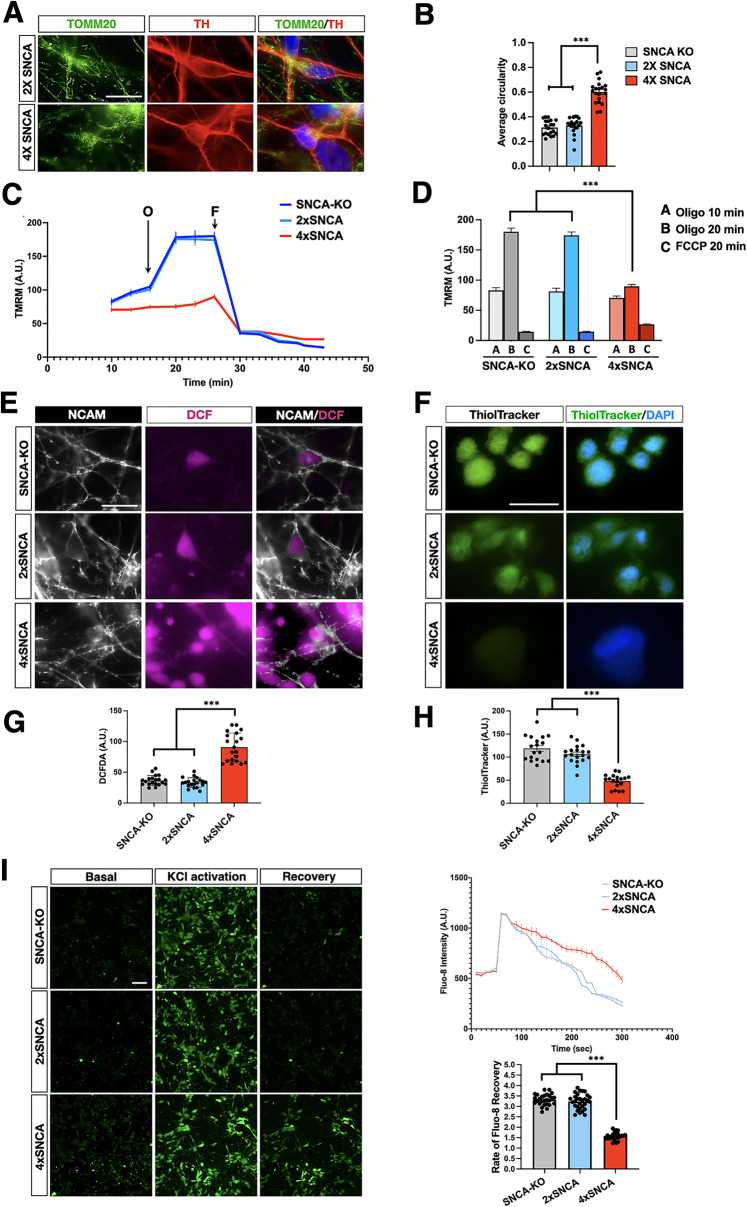
4xSNCA mDA neurons exhibit mitochondrial dysfunctions, heightened oxidative stress, and calcium mishandling. **A** Representative images of mitochondrial morphology stained with TOMM20 (green) in 2x and 4x*SNCA* mDA. **B** Quantification of mitochondrial morphology confirming the fragmented morphology in 4x*SNCA* mDA compared to isogenic control. Dots indicate quantifications in five different fields for four independent experiments. **C**, **D** Analysis of the mitochondrial membrane potential by TMRM live staining. TMRM signal is followed over time during exposure to Oligomycin (O) and FCCP (F) in SNCA-KO, 2x, and 4x*SNCA* DA neuronal cultures. Quantification of the TMRM signal is normalized on the number of cells (*n* = 25 somata). **E**, **F** Representative images of neuronal cultures stained with the ROS-sensitive fluorescent probe DCFDA (**E**) and ThiolTracker Violet (**F**). The antibody for the Neural Cell Adhesion Molecule (NCAM) is employed for live staining analysis of neurons. **G**, **H** Quantification of DCFDA (seven fields; three biologically independent experiments) and ThiolTracker Violet fluorescence (six fields; three biologically independent experiments) reveals a heightened oxidative state in 4x*SNCA* DA neurons. **I** Representative Ca^2+^ images in response to KCl and single-cell trace showing delayed recovery after KCl stimulation in 4x*SNCA* mDA neurons. Quantification of the Fluo-8 recovery rate is calculated from the Fluo-8 intensity values recorded 80 s after peak stimulation with KCl. Values are mean ± SEM. ***p < 0.001. Statistical analysis is performed using one-way ANOVA followed by Tukey post-test. Scale bars, 100 µm.

Given the significant physical interaction between αSyn aggregates and mitochondria, we thoroughly evaluated mitochondrial morphology and functionality using several readouts in 3-week-old 4x*SNCA* and control DA neuronal cultures. Immunostaining for the protein of the external mitochondrial membrane TOMM20 showed a filamentous mitochondrial network in 2x*SNCA* DA neurons, which was significantly more fragmented in 4x*SNCA* cellular counterparts (Fig. 3A, B). Collectively, these results indicated that 4x*SNCA* neurons have a global altered mitochondrial network. Next, we sought to determine whether these alterations might affect the overall bioenergetic profile of these cells. Thus, we evaluated the mitochondrial membrane potential (MMP) as determined by loading with TMRM, a cationic fluorescent dye that accumulates in negatively charged, polarized mitochondria and is released when MMP decreases. Under control conditions, 4x*SNCA* neurons showed a lower basal MMP than 2x*SNCA* or *SNCA*-KO neurons. In 2x*SNCA* DA and *SNCA*-KO neurons, the addition of the ATP-synthase inhibitor oligomycin caused an expected hyperpolarization of MMP (Fig. 3C, D). In contrast, 4x*SNCA* neurons showed a depolarization of MMP in response to oligomycin, suggesting that the mitochondrial respiratory chain was not sufficient to maintain a proton motive force, and that the ATP-synthase was working in reverse mode to maintain a physiological MMP (Fig. 3C, D). An adverse effect of impaired respiration is the increase of radical oxygen species (ROS) generation. Thus, we monitored the intracellular oxidants using the fluorescent ROS-sensitive 2′-7′-dichlorodihydrofluorescein diacetate (DCFDA) on 4x*SNCA* and isogenic control neurons in basal conditions. Notably, ROS levels were strongly enhanced in the 4x*SNCA* compared to control DA neurons (Fig. 3E, G). We, then, measured the reduced form of glutathione using the ThiolTracker Violet probe. In line with the heightened ROS levels, significantly lower levels of reduced glutathione were detected in 4x*SNCA* neurons compared to 2x*SNCA* or *SNCA*-KO cell counterparts (Fig. 3F, H). Mitochondrial dysfunctions can lead to altered regulation of calcium response, and, thus, we wondered whether calcium handling was affected by αSyn heightened levels in 4x*SNCA* DA neurons. We stimulated DA neuronal cultures after 3 weeks of differentiation with KCl (50 mM) to induce plasma membrane depolarization, neurotransmitter release, and the opening of ligand- and voltage-gated calcium channels. We recorded calcium influx using the cell-permeable fluorescent calcium dye Fluo-8. Both 4x*SNCA* and isogenic control DA neurons showed a robust cytosolic calcium peak after KCl stimulation, confirming the presence of functional calcium channels on the neuronal cell membrane (Fig. 3I). However, neurons with *SNCA* multiplication exhibited a significant delay in cytosolic calcium recovery also over an extended period of time (4x*SNCA*: 3.4 ± 0.8; 2x*SNCA:* 3.3 ± 0.9; *SNCA*-KO*:* 1.5 ± 0.4 recovery rate) (Fig. 3I). These results suggest that mitochondrial dysfunctions and calcium buffering alterations are early pathological signs in 4x*SNCA* DA neurons. Aberrant accumulation of αSyn assemblies have been shown to derange intracellular degradation processes in multiple ways [24, 25]. Accordingly, we detected enhanced mitophagy in 4x*SNCA* DA neurons as assessed by the simultaneous live staining of mitochondria and autophagolysosomes with the MitoTracker green and Lysotracker Red, respectively (Fig. S11A). On the same line, levels of total LC3-GFP and lipidated LC3-II form were found markedly increase in 4x*SNCA* DA neurons, suggesting altered autophagy process dynamics (Fig. S11B, C).

### 4x*SNCA* DA neurons show accelerated αSyn accumulation and heightened cell death

**Fig. 4:**
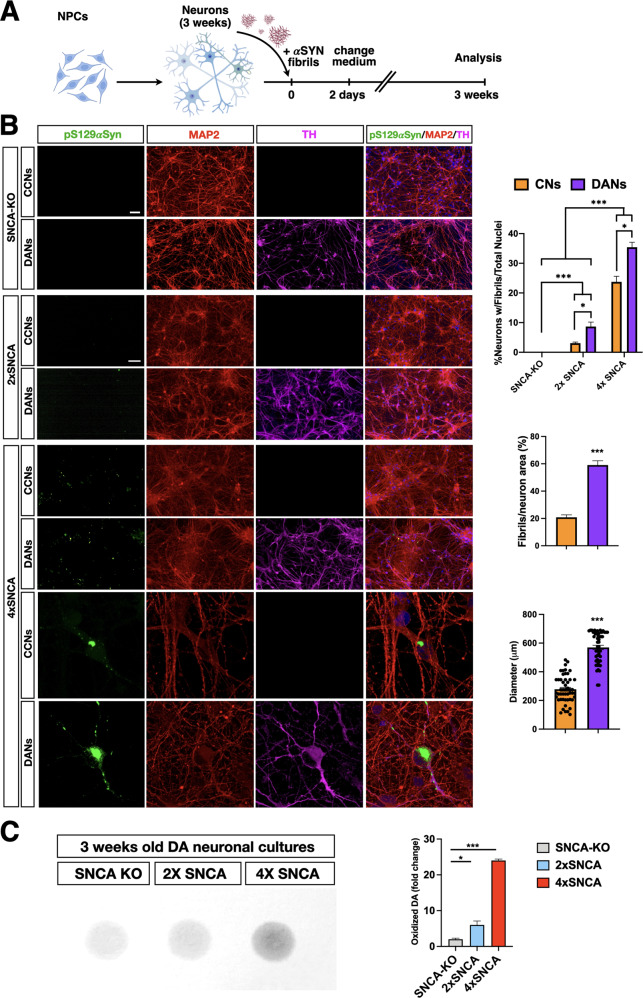
Accelerated accumulation of αSYN aggregates in 4xSNCA mDA compared to cortical neurons. **A** Schematic view of the protocol to intoxicate cortical and DA neuronal cultures with P91 αSyn fibrils. **B** Immunostaining for pS129αSYN in *SNCA*-KO 2x and 4x*SNCA* iPSC-derived cortical and DA neuronal cultures. Representative high-magnification images of pS129αSyn+ aggregates in 4x*SNCA* iPSC-derived cortical and DA neuronal cultures after exposure to P91 fibrils. Quantification of the number of neurons exhibiting pS129αSyn+ aggregates (three3 fields; three biologically independent experiments) and the ratio between the areas of aggregates and somata (*n* = 25 somata). αSYN aggregate size was calculated by measuring the perpendicular axis with respect to the larger diameter for all assemblies (50 aggregates in total). **C** Near-infrared fluorescence to profile oxidized dopamine and quantification shows high levels in 4x*SNCA* DA neuronal cultures compared to control and SNCA-KO DA neuronal cultures. Values are mean ± SEM. *p < 0.05, ***p < 0.001. Statistical analysis is performed using the Student *t*-test, one-way ANOVA followed by Tukey post-test, and two-way ANOVA followed by Bonferroni post-test. Scale bars, 100 µm.

**Fig. 5:**
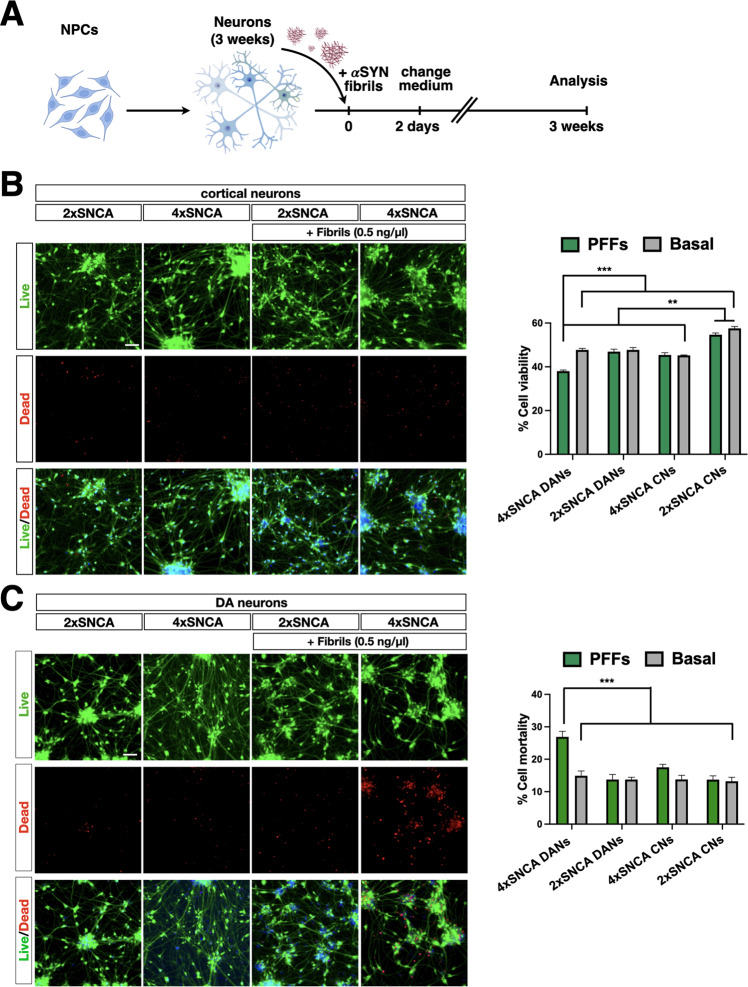
Impaired survival of 4xSNCA mDA neurons exposed to fibrils. **A** Schematic view of the protocol to intoxicate cortical and DA neuronal cultures with P91 αSyn fibrils. **B** Live/dead fluorescence staining and quantification of iPSC-derived cortical neurons with or without exposure to P91 fibrils. **C** Live/dead fluorescence staining in iPSC-derived DA neurons with or without exposure to P91 fibrils and relative quantification (for each group *n* = 50 fields, automatic counts). Values are mean ± SEM of *n* = 3 biologically independent experiments. **p < 0.01, ***p < 0.001. Statistical analysis is performed using two-way ANOVA followed by the Bonferroni post-test. Scale bars, 100 µm.

To determine whether the addition of exogenous αSyn fibrils assembled in vitro can seed the aggregation of endogenous αSyn in either cortical or DA neuronal cultures, we monitored the accumulation of pS129αSyn which occurred only during pathological αSyn aggregation. Synthetic αSyn fibrils91 were added into the culture medium of 3 weeks old neurons (0.5 ng/µl) and the extent of seeding was assessed by pS129αSyn immunohistochemistry 3 weeks later (Fig. 4A). pS129αSyn was analyzed and compared between cortical (CNs) and DA neuronal (DANs) cultures differentiated from 4x*SNCA* or isogenic control iPSCs (Fig. 4B). Interestingly, a significantly higher fraction of both 4x*SNCA* cortical and DA neurons were positive for pS129αSyn staining when compared to their relative control cellular counterparts (Fig. 4B) (4x*SNCA*-CNs: 24% ± 5; 4x*SNCA*-DANs: 35% ± 4; 2x*SNCA*-CNs: 3% ± 1; 2x*SNCA*-DANs: 8% ± 5). Importantly, *SNCA*-KO DA neuronal cultures exposed to synthetic αSyn fibrils91 were negative for pS129αSyn immunostaining, confirming that the pS129αSyn signal strictly related to the aggregation of endogenous αSyn (data not shown). This indicates that the relative abundance of endogenous αSyn is a key determinant of the extent of its pathological aggregation. Moreover, a direct comparison between DA and cortical neurons with the same 4x*SNCA* genotype showed that the former population presented more numerous pS129αSyn-positive cells with larger aggregates both in soma and neurites (4x*SNCA*-CNs: 20% ± 3; 4x*SNCA*-DANs: 58% ± 6) (Fig. 4B). Calcium dysregulation and mitochondrial oxidative stress induce dopamine oxidation with the formation of oxidized toxic intermediates that stimulate ROS production, protein dysfunction through cysteinyl residue formation, glutathione inactivation and αSyn aggregate stabilization [26, 27]. We, then, employed near-infrared fluorescence to profile oxidized dopamine levels that were substantially increased in 3-week-old 4x*SNCA* DA neuronal cultures when compared to control and *SNCA*-KO cellular counterparts (Fig. 4C). Thus, heightened oxidized DA products can additionally contribute to the induction and following stabilization of αSyn aggregation on foot of the rapid accumulation of αSyn deposits in 4x*SNCA* DA neurons. Next, we evaluated the consequences of αSyn aggregation on the fitness of the neuronal cultures in 3 weeks in vitro cultures. Simultaneous two-color fluorescence discrimination between live and dead cells based on intracellular esterase activity and plasma membrane integrity showed that 4x*SNCA* DA neurons treated with αSyn fibrils displayed a marked loss of survival three weeks after initial treatment (Fig. 5). In addition, some viability loss was also detectable although to a much lower magnitude in 4x*SNCA* cortical neurons and 2x*SNCA* DA neurons with or without pre-formed fibrils when compared to 2x*SNCA* cortical neurons (+fibrils: 4x*SNCA*-DANs 27% ± 3; 4x*SNCA*-CNs 18% ± 3; 2x*SNCA*-DANs 15% ±;3; 2x*SNCA*-CNs 14% ± 2. Basal conditions: 4x*SNCA*-DANs 15% ± 3; 4x*SNCA*-CNs 14% ± 2; 2x*SNCA*-DANs 14% ± 3; 2x*SNCA*-CNs 14% ± 2) (Fig. 5). Taken together, the rapid endogenous αSyn aggregation induced through seeding by exogenous αSyn fibrils91 leads to a loss of cell viability mostly evident in DA 4x*SNCA* DA neurons preceded by large pS129αSyn inclusions and aberrant levels of oxidized DA.

### Generation of Lewy body-like structures in 4x*SNCA* DA neurons upon seeding

**Fig. 6:**
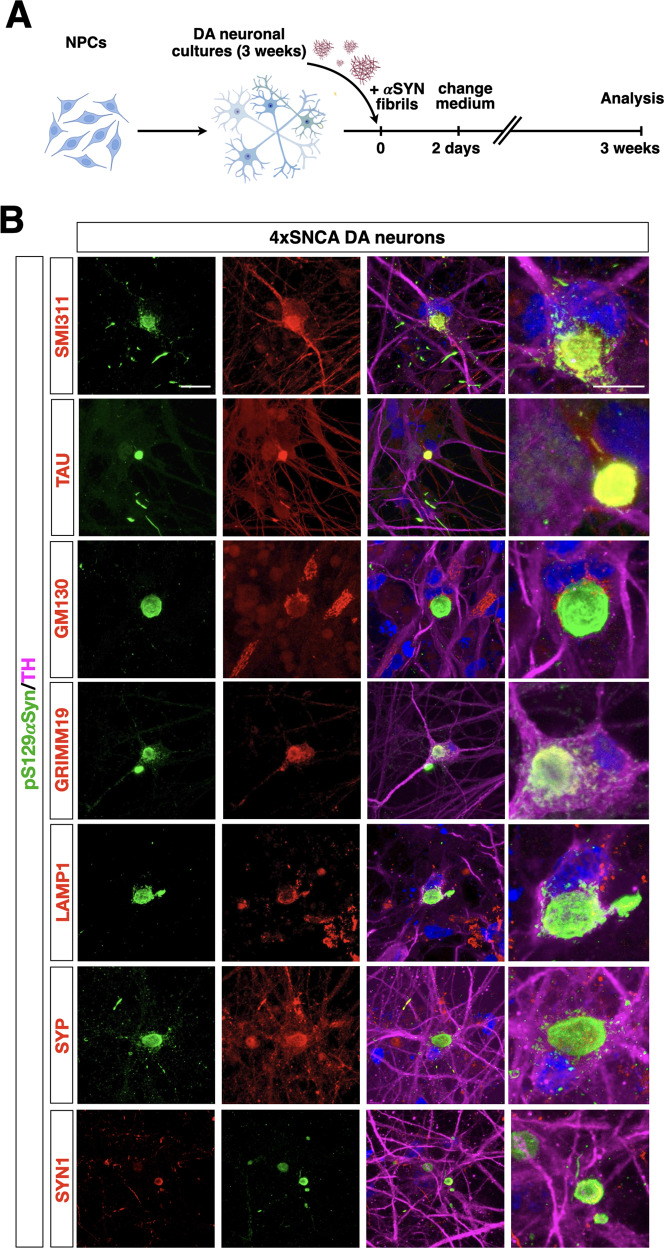
Generation of Lewy body-like structures in seeded 4xSNCA mDA neurons. **A** Schematic view of the protocol to intoxicate DA neuronal cultures with P91 αSyn fibrils. **B** Representative images of Lewy body-like structures stained with different markers for neurofilament (TAU), mitochondria (GRIMM19), lysosomes (LAMP1), cis-Golgi membranes (GM130), and synaptic protein (SYN1, SYP). Scale bar, 100 µm.

Three weeks after initial seeding with exogenous αSyn fibrils91 4x*SNCA* DA neurons were the only population to develop numerous large round deposits within the somata in close vicinity with the nucleus and smaller and elongated inclusions along the neurites positive for pS129αSyn (Fig. 6A). Next, we performed triple immunohistochemistry to characterize the organization and internal constituents of these structures. The majority of these inclusions were uniformly immunodecorated by antibodies recognizing neurofilaments and the microtubule-associated protein TAU (Fig. 6B). Moreover, most of these structures, both in the soma and neurites, were associated with organelles as shown by the co-staining of selective markers for mitochondria (GRIMM19) and lysosomes (LAMP1). GM130 positive cis-Golgi membranes were often found to partially enwrap pS129αSyn deposits in the soma (Fig. 6B). Finally, the two resident pre-synaptic proteins Synaptophysin (SYP) and Synapsin-1 (SYN1) were found ectopically clustered within the pS129αSyn deposits, suggesting that a significant fraction of synaptic vesicles got entrapped into these structures (Fig. 6B). We next performed correlative light-electron microscopy (CLEM) combining fluorescence with electron microscopic analysis on 5 weeks old iPSC-derived neurons treated with αSyn fibrils. The ultrastructural assessment revealed excessive crowding of vesicles and membranous structures both at the periphery and at the core of the αSyn-positive inclusions (Fig. S12A). Moreover, altered numerous mitochondria with abnormal morphology were located at the border of the αSyn-positive structures (Fig. S12A). Finally, immunoblots with the insoluble fraction of the cell lysates detected higher molecular species of αSyn only in 2-week-old 4x*SNCA* DA neuronal cultures treated with exogenous fibrils (Fig. S12B), suggesting that αSyn aggregation is advanced only in 4x*SNCA* neurons at this stage in vitro. Thus, we renamed αSyn inclusions in 4x*SNCA* neurons as Lewy body-like structures, given their resemblance in both morphological traits and associated organelles with typical Lewy bodies detectable in most PD brain tissues [28]. Collectively, these results show that the relatively high abundance of native αSyn protein levels in 4x*SNCA* DA neurons predispose them, upon an exogenous seeding trigger, to the rapid development of Lew body-like structures.

### Disruption of TAX1BP1 strongly exacerbates αSyn pathology in DA neurons

**Fig. 7:**
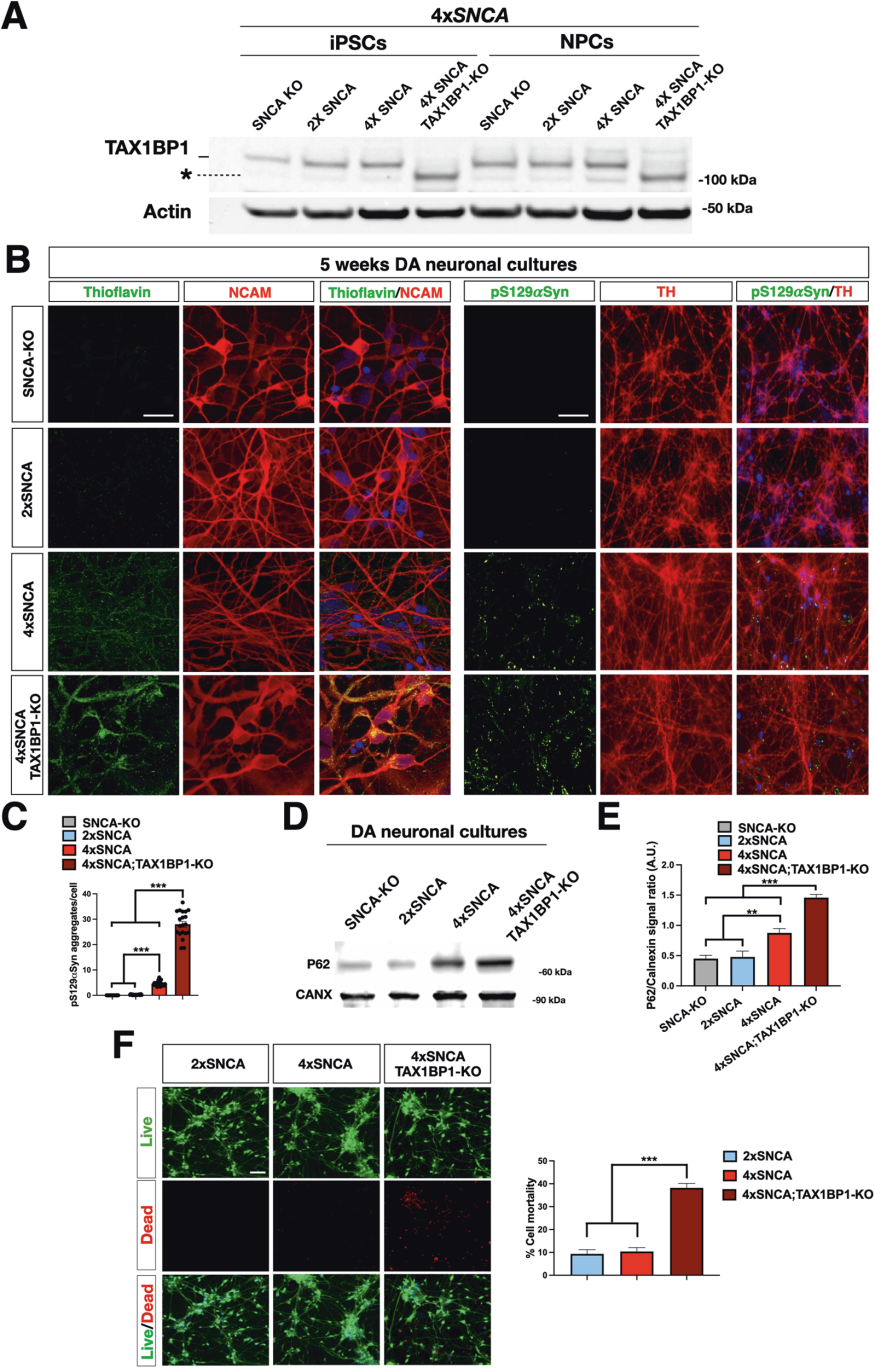
TAXBP1 inactivation exacerbates αSyn aggregates in DA neurons. **A** Immunoblot analysis for the TAX1BP1 protein confirms the inactivation of the gene in 4x*SNCA*;TAXBP1-KO iPSCs. * indicates a non-specific signal. **B** Representative images of 4x*SNCA*;TAXBP1-KO stained with Thioflavin S and pS129αSyn. **C** Quantification of the number of pS129αSyn+ aggregates in SNCA-KO, 2x, 4x*SNCA*, and 4x*SNCA*;TAXBP1-KO DA neurons. Dots represent quantifications in five fields for four independent experiments. **D**, **E** Immunoblot and quantification of p62 signal in SNCA-KO, 2x, 4x*SNCA*, and 4x*SNCA*;TAXBP1-KO DA neurons (*n* = 3 independent experiments). **F** Live/dead fluorescence staining and quantification of cell viability and toxicity 2x, 4x*SNCA*, and 4x*SNCA*;TAXBP1-KO DA neurons. Values are mean ± SEM of *n* = 3 independent experiments (*n* = 50 fields for each group, automatic counts). **p < 0.01, ***p < 0.001. Statistical analysis is performed using one-way ANOVA followed by Tukey post-test. Scale bars, 100 µm.

Protein aggregates are preferentially eliminated by selective autophagy (aggrephagy). TAX1BP1 is an autophagy receptor with a key role in promoting the clearance of Huntingtin and polyQ protein inclusions [29]. However, whether TAX1BP1 has a similar role in αSyn protein quality control has remained yet unaddressed. Thus, we decided to inactivate *TAX1BP1* in the 4x*SNCA* iPSC line by CRISPR/Cas9 gene editing. We selected a sgRNA targeting exon 4, which is in common with all the five *TAX1BP1* gene isoforms and induced indel mutations by transient transfection of spCas9 and sgRNA expressing plasmids followed by antibiotic selection in proliferating iPSCs. We selected one iPSC line based on the following criteria: the presence of indel mutations harboring frameshift in both *TAX1BP1* gene copies, protein loss by Western blotting, correct colony morphology, stable growth, and homogeneous expression of the pluripotency genes (Figs. 7 and S13). Next, *SNCA* iPSCs with or without *TAX1BP1* gene deletion were differentiated in DA neuronal cultures for 5 weeks and then inspected for the accumulation of native αSyn aggregates by Thioflavin S staining and pS129αSyn immunohistochemistry. As reported earlier, among *SNCA* DA neuronal cultures only in the presence of 4x*SNCA*, scattered pS129αSyn aggregates were detectable at this time point. However, additional *TAX1BP1* gene inactivation triggered a 5- and 3-fold increase of Thioflavin S and pS129αSyn staining, respectively (Fig. 7B, C). Exacerbated protein aggregation in 4x*SNCA;TAX1BP1*-KO DA neuronal cultures was confirmed by the highest levels of P62 protein accumulation among the cell lines in analysis (Fig. 7D, E). Moreover, only after the *TAX1BP1*-KO gene targeting a significant loss of viability was ascertained in DA neuronal cultures by using a two-color fluorescence live/dead assay (2x*SNCA:* 9% ± 3; 4x*SNCA* % ± 3; 4x*SNCA;TAX1BP1:* 36% ± 5) (Fig. 7F). Thus, 4x*SNCA;TAX1BP1*-KO DA neuronal cultures accumulated marked αSyn aggregates levels sufficient to impair neuronal viability in basal conditions without the need of any exogenous trigger.

## Discussion

Herein we generated and characterized a panel of isogenic iPSC lines derived from a PD patient with one of the smallest genomic triplication of *SNCA*. Using CRISPR/Cas9 gene editing, each of the 4 *SNCA* genes was destroyed with indel mutations inducing frameshift or exon 3/4 deletions, generating a set of isogenic iPSCs with an incremental number of *SNCA* gene alleles from 4 (parental line) to none (*SNCA*-KO). PD patients with *SNCA* triplication are extremely rare, and for this reason, the multiple studies in the literature were performed using iPSCs derived from only two individuals, a 48 years old male [17, 30] and a 55 years old female [16, 18]. Most of the early iPSC lines were generated by integrating reprogramming vectors, and only in one case, an isogenic control iPSC line is available, although its corresponding parental line was reprogrammed with retroviral vectors [15]. Thus, the new set of isogenic iPSCs generated in this study significantly expands the number of iPSC lines available for this important PD-causing genetic alteration and provides for the first time a full panel of isogenic cells that differ for only the incremental number of *SNCA* genes. Generating iPSCs from more individuals with *SNCA* triplication is also extremely relevant for identifying ultimately the molecular determinants explaining symptomatic differences between patients. In fact, despite the general severity of the disease phenotype in these patients, the two siblings in this study presented substantial differences in clinical motor and cognitive manifestations with different ages of onset [19]. Thus, a wide collection of iPSCs from these patients, together with their deep, homogeneous, and longitudinal clinical assessments, would represent a powerful resource to investigate *SNCA* genetic modifiers.

We showed that αSyn protein levels increase in accordance with the number of functional *SNCA* genes reaching a maximal peak in 4x*SNCA* cells. We showed that endogenous αSyn abundance was associated with an early αSyn aggregation in 2 weeks old DA neuronal cultures. These conclusions were reached by exploiting the new nanobody-based biosensor FluoReSyn which provided a sensitive and effective tool to visualize small αSyn assemblies when coupled with super-resolution imaging. Its high specificity was confirmed by the lack of any meaningful staining in isogenic *SNCA*-KO cells. These results emphasize the importance to have also available an *SNCA* knock-out cell line in the panel of iPSCs for specificity readouts. This analysis provided a neat description of the development of endogenous αSyn aggregates in early differentiated DA neurons in basal conditions without any exogenous stimuli. This finding identified 4x*SNCA* DA neurons as an excellent cellular system to investigate biophysical determinants of αSyn aggregation and identify specific inhibitors of this process by candidate testing or screening campaigns. These data were in line with previous observations, in which αSyn aggregates in 4x*SNCA* DA neurons were inferred by the proximity ligation assay (PLA) [13]. However, this is a cumbersome technique with extremely low throughput. In contrast, AAV-based FluoReSyn expression enables a direct, multi-color, and rapid assessment of αSyn assemblies on in vitro neuronal cultures, which is compatible with longitudinal analysis and time-lapse imaging. As a valuable example of this, FluoReSyn staining enabled us to identify a significant proportion of αSyn aggregates associated with mitochondria in both early and late neuronal cultures. This finding prompted us to investigate mitochondrial alterations in 4x*SNCA* DA neurons, revealing the occurrence of mitochondrial fragmentation, loss of membrane potential, and heightened oxidative damage. Interestingly, in concomitance with mitochondrial dysfunctions, we observed altered calcium signaling in 4x*SNCA* DA neurons with a significant delay in cytosolic calcium efflux after KCl stimulation. High cytosolic calcium levels may also favor the opening of the mitochondrial permeability transition pore (PTB), which induces loss of the mitochondrial membrane potential [31, 32]. Thus, calcium mishandling can represent an early alteration in 4x*SNCA* DA neurons which destabilizes mitochondrial homeostasis.

In addition, we showed that upon the challenge to exogenous αSyn fibrils to accelerate endogenous αSyn seeding, 4x*SNCA* DA neurons showed the highest burden of αSyn aggregates, which significantly impaired their survival in culture. In fact, 4x*SNCA* DA neurons showed the largest cell fraction with accelerated development of αSyn deposits as compared to 4x*SNCA* cortical neurons and isogenic control 2x*SNCA* neurons. This selective impairment should be routed within the specific DA metabolism. In fact, we found aberrant levels of oxidized DA in seeded 4x*SNCA* DA neurons, which can further enhance oxidative damage and, thereby, accelerate αSyn aggregation. Given that altered oxidized DA has been already described in iPSC-derived neurons of other idiopathic and familiar PD patients [14], its accumulation can be considered a key event in the pathophysiological mechanisms leading to DA neuronal degeneration by enhancing oxidative damage and facilitating αSyn aggregation and, thereby, establishing a detrimental vicious cycle between these two pathological events.

We showed that over time the αSyn aggregates in seeded 4x*SNCA* DA neurons developed over time into large deposits localized in soma and processes. Immunohistochemistry analysis indicated that they are enriched in mitochondrial and lysosomal components suggesting a stable association with these organelles or part of them. Intriguingly, a recent ultrastructural analysis in *post-mortem* patient brain tissues nicely showed that the major constituents of Lewy bodies are dysmorphic organelles and vesicles intermingled with heterogeneous membrane fragments [28]. Given the high resemblance of their morphological structures, we consider it appropriate to refer to αSyn aggregates in seeded 4x*SNCA* DA neurons as Lewy body-like structures. Similar findings were reported by Mahul-Mellier et al. [33], applying advanced imaging approaches to study the dynamics of the formation of seeded αSyn aggregation in mouse hippocampal neurons. These results indicate that αSyn aggregation, when forced through exogenous seeding, can follow similar principles of spatial organization and internal constituents between different neuronal cell types. However, our results also show that only 4x*SNCA* DA neuron viability was severely affected by αSyn aggregation, highlighting that cell-type specific pathophysiological consequences caused by the development of these aggregates require the generation of authentic in vitro counterparts of midbrain-specific DA neurons.

Collectively, our data suggest that 4x*SNCA* DA neurons offer a remarkable cellular platform to investigate mechanisms and mediators of native and seeded αSyn aggregation. As proof of principle for this, we assessed the role of TAX1BP1, a key autophagy receptor, in this scenario.

Simultaneous inactivation of *TAX1BP1* by CRISPR/Cas9 gene editing in 4x*SNCA* DA neurons showed that αSyn aggregation is more aggressive and diffuse, revealing a key role of TAX1BP1 in modulating the clearance of αSyn aggregates. In addition, *TAX1BP1* gene loss sensitizes 4x*SNCA* DA neurons to cell death even in the absence of exogenous stimulated seeding. This result indicates that TAX1BP1 deficiency enhances total endogenous αSyn protein content, which jeopardizes the fitness and survival of 4x*SNCA* DA neurons.

In conclusion, we produced iPSCs starting from a PD patient harboring a new and compact *SNCA* triplication and using CRISPR/Cas9 gene editing, we generated an isogenic allelic series of iPSCs by inactivating each of the *SNCA* alleles. By differentiating midbrain DA neuronal cultures, we identified the early accumulation of endogenous αSyn aggregates by combining a new and sensitive biosensor with super-resolved imaging. In addition, we revealed novel cellular dysfunctions in both native and seeded conditions in 4x*SNCA* DA neurons which can partly explain the selective vulnerability of these neurons to αSyn aggregation burden.

## Methods

### Plasmid cloning

sgRNAs were designed using the online software CRISPOR [34] and selected according to the higher specificity score. Then, sgRNAs were cloned into the LV-U6-filler-crRNA-Ef-1α-Blasticidin vector, previously described in ref. [35]. The expression vector pCAG-spCas9-P2A-Puromicin [35] was employed for the transient expression of spCas9 in human iPSC cultures.

### Cell cultures

Peripheral blood samples from patients were obtained from the IRCCS Neuromed of Pozzilli through informed consent approved by the local Ethical Committee (Clinical Trials: #NCT03682458; ID: CGM-02). Primary peripheral blood mononuclear cells (PBMCs) were isolated from the blood withdrawals and reprogrammed by non-integrating Sendai viruses expressing the four Yamanaka’s factors using the CytoTune-2.0 kit (Thermo Fisher) at the IRCCS San Raffaele Hospital in Milan. iPSC lines were maintained in feeder-free conditions in mTeSR1 (Stem Cell Technologies) and expanded in HESC-qualified Matrigel (Corning)-coated six-well plates.

### Gene editing

iPSCs were maintained in feeder-free conditions in mTeSR1 (Stem Cell Technologies) and seeded in HESC-qualified Matrigel (Corning)-coated six-well plates. At >80% confluency, the iPSCs were transfected with two separate plasmids expressing sgRNA-blast and spCas9-Puro using Lipofectamine^TM^ Stem Reagent (Thermo). Co-transfected colonies were then selected by the combination of puromycin (1 µg/ml, Sigma) and blasticidin (10 μg/ml, Thermo Fisher Scientific) and then isolated by single colony picking. Finally, cell clones with the correct genomic deletions were assessed by genomic PCR analysis followed by Sanger sequencing.

### Neuronal differentiation

NPCs were generated as previously described with appropriate optimization [36]. Briefly, iPSCs were dissociated in cell clusters using Accutase (Sigma-Aldrich) and seeded onto low-adhesion plates in mTeSR1 supplemented with N2 (1:200, Thermo Fisher Scientific), Pen/Strept (1%, Sigma-Aldrich), human Noggin (0.5 μg/ml, R&D System), SB431542 (5 μM, Sigma-Aldrich), and Y27632 (10 μM, Selleckchem). After 10 days, embryoid bodies were seeded onto matrigel-coated plates (1:100, matrigel growth factor reduced, Corning) in DMEM/F12 (Sigma-Aldrich) supplemented with N2 (1:100), non-essential amino acids (1%, MEM NEAA, Thermo Fisher Scientific), and Pen/Strept. After 10 days, rosettes were dissociated with Accutase and plated onto matrigel-coated-flasks in NPC media containing DMEM/F12, N2 (1:200), B27 (1:100, Thermo Fisher Scientific), Pen/Strept (1%) and FGF2 (20 ng/ml, Thermo Fisher Scientific). For differentiation, NPCs were dissociated with Accutase and plated on matrigel-coated six-well plates (1 × 300,000 cells per well) in NPC medium. Two days after, the differentiation medium containing Neurobasal (Thermo Fisher Scientific), Pen/Strep (1%), B27 (1:50), with SU5402 (Sigma-Aldrich, 10 µM), PD0325901 (Sigma-Aldrich, 8 µM), DAPT (Sigma-Aldrich, 10 µM) was added and kept for 2 days. The differentiation medium was replaced every day with a fresh one on days 1 and 2. On day 3, cells were detached by Accutase solution incubation at 37 °C for 10 min in order to obtain a single-cell suspension. Cells were centrifuged, counted, and seeded at a density of 55,000 cells/cm^2^ onto poly-l-lysine/laminin/fibronectin (all from Sigma-Aldrich, 100 µg/ml, 2 µg/ml, 2 µg/ml)-coated plates in neuronal maturation medium supplemented with ROCK inhibitor Y27632 (10 µM) for the first 24 h. Neuronal maturation medium was composed of Neurobasal A (Thermo Fisher Scientific) supplemented with 1× B27 supplement, 2 mM glutamine, 1% Pen/Strept, BDNF (Peprotech, 20 ng/ml), ascorbic acid (Sigma-Aldrich, 100 nM), Laminin (1 μg/μl), DAPT (10 μM), and dbcAMP (Selleckchem, 250 μM). The culture medium was replaced the next day to remove the ROCK inhibitor, and then half of the medium was replaced with a fresh neuronal maturation medium twice a week.

Dopaminergic neurons were generated as previously described with small modifications [37, 38]. iPSCs were dissociated with Accutase and plated on matrigel-coated six-well plates (1 × 200.000 cells per well) in mTeSR1 medium. One day after, the medium was replaced by a differentiation medium containing LDN193189 (100 nM, Stemgent), SB431542 (10 mM, Tocris), SHH C25II (100 ng ml21, R&D), Purmorphamine (2 mM, Sigma-Aldrich), FGF8 (100 ng/ml, Sigma-Aldrich), and CHIR99021 (CHIR; 3 mM, Milteny) in mTeSR1 medium for 11 days. mTeSR1 medium was gradually shifted to N2 medium starting on day 5 of differentiation. The half medium was changed every 2–3 days. After 9 days, cells were dissociated with Accutase and plated on poly-l-lysine/laminin-coated 24-well plates for the final maturation. BDNF (10 ng/ml), GDNF (10 ng/ml), DAPT (10 μM, Sigma-Aldrich), and Ascorbic Acid (10 μM, Sigma-Aldrich) were added from day 20 to promote neuronal maturation and survival.

### MPLA analysis

The commercially available kit P051-P052 (MRC- Holland, Amsterdam, Netherlands) was used for the multiplex dosage of exons in iPSC genomic DNA for the following genes: TNFRSF9 (one probe in P051), DJ1 (four probes in P051), ATP13A2 (two probes in P051, two probes in P052), SNCA (five probes in P051, one probe in P052), LPA (one probe in P051), PARKIN (12 probes in P051, 12 in P052), LRRK2 (eight probes in P052), PINK1 (eight probes in P051), GCH1 (five probes in P052), PACRG (one probe in P052), CAV1/2 (two probes in P052), and UCHIL1 (four probes in P052).

### αSyn fibrils91 preparation

Human wild-type *SNCA* was expressed in *E. coli* BL21 DE3 CodonPlus cells (Agilent Technologies) and purified as described previously [39]. Pure monomeric αSyn was assembled into the fibrillar polymorph, named fibrils91, as it exhibits the highest seeding potency in neurons in vitro and in vivo [40, 41] in 20 mM KPO4, 150 mM KCl at 37 °C under continuous shaking in an Eppendorf Thermomixer set at 600 r.p.m for 7 days [42]. The aggregation reaction was followed by withdrawing aliquots (10 µl) from the reaction at different time intervals, mixing them with Thioflavin T (400 µl, 10 µM final) and recording the fluorescence increase on a Cary Eclipse Fluorescence Spectrophotometer (Varian Medical Systems Inc.) using an excitation wavelength = 440 nm, an emission wavelength = 480 nm and excitation and emission slits set at 5 and 10 nm, respectively. The resulting αSyn fibrils91 were centrifuged twice at 15,000 × *g* for 10 min and re-suspended twice in PBS at 250 µM. αSyn fibrils91 were fragmented by sonication for 20 min in 2 mL Eppendorf tubes in a Vial Tweeter powered by an ultrasonic processor UIS250v (250 W, 2.4 kHz; Hielscher Ultrasonic, Teltow, Germany) to generate fibrillar particles with an average size of 42–52 nm as assessed by TEM analysis. The fibrillar nature of aSyn was assessed by Transmission electron microscopy (TEM) after adsorption of the fibrils onto carbon-coated 200 mesh grids and negative staining with 1% uranyl acetate using a Jeol 1400 transmission electron microscope before and after fragmentation. The images were recorded with a Gatan Orius CCD camera (Gatan, Pleasanton, CA, USA). We further quantified the endotoxin levels in αSyn fibrils91 preparations as described previously [43, 44] to make sure that endotoxin levels were below 0.02 endotoxin units/mg (EU/mg) using the Pierce LAL Chromogenic Endotoxin Quantification Kit.

### Immunocytochemistry

Cells were seeded on matrigel-coated glass coverslips and they were fixed for 20 min in ice in 4% paraformaldehyde (PFA, Sigma), solution in phosphate-buffered saline (PBS, Euroclone). Then, cells were permeabilized for 30 min in a blocking solution, containing 0.5% Triton X-100 (Sigma-Aldrich) and 10% donkey serum (Sigma-Aldrich), and incubated overnight at 4 °C with the primary antibodies in a blocking solution. Then, cells were washed with PBS and incubated for 1 h at room temperature with Hoechst and with secondary antibodies. The following antibodies were used: anti-OCT4 (1:100, Abcam), anti-NANOG (1:100, Abcam), anti-FOXA2 (1:300, Abcam), anti-NESTIN (1:300 Millipore), anti-TH (1:200, Immunological Sciences), anti-MAP2 (1:500, Immunological Sciences), anti-TOMM20 (1:300, Novus), anti-α-Synuclein (clone LB509, 1:100, Thermo), anti-GFP (1:500, Thermo), anti-α-Synuclein (phosphoS129, 1:300, Abcam), anti-TAU (1:500, Millipore), anti-LAMP1 (1:500, Abcam), anti-Synapsin-1 (1:500, Synaptic Systems), anti-SMI311 (1:500, BioLegend), anti GM130 (1:300, BD), anti-GRIM19 (1:300, Abcam). All the secondary antibodies used for the immunofluorescence staining are Alexa Fluor^TM^.

### Molecular cloning and viral production

For the LV:LC3-GFP, the LC3 coding region, followed by the GFP coding sequence, was cloned into a lentiviral vector downstream to the EF-1α promoter. For the LV:Syn-GFP, the GFP coding sequence was cloned under the control of the α-Synapsin promoter in a lentiviral vector. Replication-incompetent VSVg-coated lentiviral particles were packaged in 293 T cells.

### Cell lysate preparation and immunoblotting

Cell lysates were prepared with a buffer containing 50 mM Tris-Hcl (pH 7.4), 150 mM NaCl, 0,1% SDS and 1% Triton X-100. Protease and phosphatase inhibitor cocktails (Roche) were added immediately prior to use. The protein concentration was measured by BCA Protein Assay Kit (Thermo Fisher Scientific). Generally, 25 μg of protein lysates were loaded onto SDS-polyacrylamide gel electrophoresis and protein was transferred onto a nitrocellulose membrane. The membrane was blocked with 5% non-fat dry milk in PBST (phosphate-buffered saline with 0.1% Tween-20). For αSYN fractioning immunoblot, cell lysates were processed in order to collect the PBS- and SDS-soluble fractions as described by Luk et al. [45]. Briefly, cells were washed with PBS and scraped into ice-cold lysis buffer (50 mM Tris/150 mM NaCl/1% Triton X-100, pH 7.4) containing protease and phosphatase inhibitors. After lyses, the supernatant was clarified by 100,000 × *g* centrifugation at 4 °C for 1 h. The resulting supernatant represents the PBS-soluble fraction. Then pellets were solubilized in PBS-SDS (1% SDS w/v) by sonication and centrifuged at 100,000 × *g* for 30 min at 25 °C. Samples were loaded in a gradient gel (4–12% Bis-tris gel, NP0322BOX; Invitrogen). The transfer was performed for 1 h at 100 V on a nitrocellulose membrane (GE Healthcare). Membranes were then blocked in 5% non-fat-milk for 1 h and the primary antibody (Syn211) was incubated overnight at 4 °C. After incubation with the appropriate HRP-conjugated secondary antibody for 30 min, the signal was then revealed and processed as previously described.

The primary antibodies and dilutions used were the following: anti-ACTIN (1:1000, Sigma-Aldrich), anti-CALNEXIN (1:2000, Sigma-Aldrich), anti-P62 (1:4000, Novus), anti-α-Synuclein (clone Syn211, 1:1000, Millipore), anti-TAX1BP1 (1:1000, Bethyl Laboratories). Antibody incubation was followed by horseradish peroxidase (HRP)-conjugated goat anti-mouse and anti-rabbit secondary antibodies (1:10000, Dako). The signal was revealed using the ECL-chemiluminescence kit (GE Healthcare Amersham ECL Western Blotting Detection Reagent) and detected with ChemiDoc Touch Imaging System (Biorad). Quantitation of band intensity on unsaturated exposures was performed with the Volume tool of the Image Lab 5.0 software. The adjusted values of the proteins of interest were normalized on those of Actin or Calnexin bands of the corresponding lanes.

### Structured illumination microscopy (SIM)

Samples for SIM were cultured on laminin-coated glass coverslips. They were fixed and stained for intracellular markers as described in the Immunostaing section. SIM was performed on an Elyra (Zeiss, ELYRA 7 with Lattice SIM²), using a 60x oil objective. Images were processed and channels aligned using the automatic settings on the ZEN Black software (Zeiss).

### Mitochondrial morphology

Dopaminergic neurons from patients and isogenic controls were seeded on matrigel-coated glass coverslips. Mitochondrial morphology was assessed by TOMM20 immunostaining. Cellular fluorescence images were acquired with a Nikon Eclipse Ni microscope. Images were collected using an X63/1.4 oil objective and analyzed using the Mito-Morphology macro in ImageJ.

### Mitochondrial membrane potential determination

Neurons from patients and isogenic controls were seeded on matrigel-coated glass coverslips. Mitochondrial membrane potential was measured based on the accumulation of Tetramethylrhodamine Methyl Ester (TMRM; Life Technologies). Cells were incubated with 2 μM Oligomycin (Sigma-Aldrich) and loaded with 250 nM TMRM for 20 min. At the end of each experiment, mitochondria were fully depolarized by the addition of 1 μM of the protonophore carbonyl cyanide 4-(trifluoromethoxy) phenylhydrazone (FCCP; Sigma-Aldrich). Cellular fluorescence images were acquired every 3 min for each sample with a fluorescence microscope (Nikon Eclipse Ni) and analyzed by ImageJ.

### Mitophagy analysis

For assessment of the mitophagy, neuronal cultures were incubated with MitoTracker Green FM (1 μM final concentration) (Thermo Fisher Scientific) for 30 min at 37 °C and then extensively washed with PBS. LysoTracker Red DND-99 (1 μM final concentration) (Thermo Fisher Scientific) was then added, and cells were immediately observed on a Nikon LiveScan Confocal Microscope. The green and red signal colocalization rate was evaluated using the colocalization counter JACOP available in Fiji software. For each condition, the colocalization of these two signals was also determined by manual counting of fluorescent puncta.

### ROS and glutathione quantitative analysis

iPSC-derived neurons were incubated with Alexa Fluor 647 mouse anti-human CD56 (anti-NCAM, BD Biosciences, diluted 1:40) for 1 h, with 20 μM of 20, 70 -dichlorodihydrofluorescein diacetate (H2DCFDA; Molecular Probes) for 15 min, and with 2 μg/ml of Hoechst 33342 for 2 min at 37 °C. Cells were washed multiple times and randomly analyzed using a fluorescence microscope (Nikon Eclipse Ni). The DCFDA signal from NCAM–positive cells was quantified by ImageJ software analysis to determine the relative ROS content.

For Glutathione analysis, neurons were incubated with 20 μM ThiolTracker Violet (Invitrogen) for 30 min at 37 °C, and then, washed with PBS and analyzed by fluorescence microscope (Nikon Eclipse Ni). The ThiolTracker Violet fluorescence was collected to compare relative glutathione contents by ImageJ image analysis.

### Live/dead labeling

Cells plated on matrigel-coated glass coverslips were incubated with 5 μM of Live/Dead Assay Kit (Thermo Fischer) for 10 min at 37 °C. Then, cells were washed with PBS and acquired by an inverted fluorescence microscope (Nikon, Eclipse Ti). For the analysis, the images were collected using an X20/0.45 objective and analyzed using ImageJ.

### Measurement of [Ca^2+^]

Cells were seeded in 96 well-optic black microplates and loaded with 4 μM of Fluo-8 in KRH for 30 min at 37 °C followed by 2x KRH washes. The images were taken with a fully automated microscope, ArrayScan XTI HCA Reader (Thermo Fisher Scientific), equipped with a liquid handling module for the dispensing of the stimuli and a Photometrics X1, 14-bit, high-resolution camera. A Zeiss LD Plan-NEOFLUAR 20x/0,4NA objective was used to capture one image per well with the following settings: Fluo-8 (excitation wavelength: 485/20 nm, emission filter 520/21 nm) and Hoechst (excitation wavelength: 386/23 nm, emission filter: 440/20 nm) were detected both with camera gain2 and 60% of LED intensity. Thirty frames were acquired at 0.5 Hz with 20 msec exposure time for Fluo-8 and 10 msec exposure time for Hoechst. About 50 ul of 50 mM KCl was dispensed at a rate of 50 ul/sec. At least four baseline images were acquired. The analysis was done with HCS Studio software using SpotDetector bioapplication (Thermo Fisher Scientific). Hoechst-positive nuclei were identified and counted in all the images. The nuclei segmentation method was identical for all the conditions within the same experiment but was optimized among the experiments. The 3D_Surface Fitting method (parameter value:255) was applied for the removal of the background before the quantification of the Fluo-8 signal. The mean intensity of the Fluo-8 signal was then quantified in the cell body area of each single cell and the normalized mean intensity was obtained for each well.

### Near-infrared fluorescence for oxidized dopamine assessment

Oxidized dopamine analysis were performed as described by Burbulla et al. [14]. Briefly, for each experiment, 1 x 10e6 neurons were harvested and cell pellets were homogenized in 1% Triton X-100 lysis buffer. Insoluble pellets were extracted in 2% SDS/50 mM Tris by boiling and sonication. Leftover insoluble pellets from a 150,000 × *g* spin (30 min, 4 °C) were further extracted in 1 N NaOH, followed by incubation at 55 °C. Then, the solutions were lyophilized in a Speed Vac Concentrator until the pellet was completely dry. Pellets were washed once with ultrapure H_2_O for removal of hydroxides, and then lyophilized again before the dried pellet was taken up in ultrapure H_2_O and finally analyzed. About 10 mM oxidized dopamine (DA) stock was used as a standard and prepared to start from 10 mM DA (in D-PBS) mixed with 20 mM NaIO_4_. Each experimental sample or standard dilution was dropped onto Nylon membranes and scanned using an Odyssey infrared imaging system. Samples were quantified by obtaining integrated spot intensities using Odyssey infrared imaging software (LI-COR).

### Measurement of α-Syn protein levels in the cell culture supernatant

For the quantification of the α-Syn protein, neurons were plated on laminin-coated six-well plates. Collection of the medium was carried out after 7 days in which the medium was not changed and the analysis of protein in cell culture was performed with a Human Alpha-Synuclein ELISA Kit (Abcam).

### ThT fluorescence staining

iPSC-derived neurons were incubated with Alexa Fluor 647 mouse anti-human CD56 (anti-NCAM, BD Biosciences, diluted 1:40) for 1 h, with 1 mM of Thioflavin T (Sigma-Aldrich) for 15 min, and with 2 μg/ml of Hoechst 33342 for 2 min at 37 °C. Cells were washed multiple times and randomly analyzed using a fluorescence microscope (Nikon Eclipse Ni).

### Correlative light-electron microscopy (CLEM)

Cells were grown on finder grids and Z-stacks of cells of interest were taken with the PerkinElmer UltraView ERS confocal microscope. The coordinates of the cells on the finder grid were determined by bright-field microscopy. Cells were fixed in 1% glutaraldehyde in 0.1 M cacodylate buffer (Sigma) and post-fixed with 1.5% potassium ferricyanide, 1% OsO4 in 0.1 M cacodylate buffer. Cells were stained with 0.5% uranyl acetate overnight, dehydrated in ethanol, and embedded in epon. After baking for 48 h at 60 °C, the resin was released from the glass coverslip by temperature shock in liquid nitrogen. Serial sections (70–90 nm) were collected on carbon-coated formvar slot grids and imaged with a Zeiss LEO 512 electron microscope. Images were acquired by a 2k × 2k bottom-mounted slow-scan Proscan camera controlled by EsivisionPro 3.2 software. Immunofluorescence and IEM images were aligned using Icy bioimage analysis.

### Statistics

All values are expressed as mean ± SEM. Differences between means were analysed using the Student *t*-test, one-way or two-way analysis of variance (ANOVA) depending on the number of groups and variables in each experiment. In vitro and in vivo data were then submitted to Tukey or Bonferroni post hoc test using GraphPad Prism software. The null hypothesis was rejected when the *P* value was <0.05.

## Supplementary information

Supplementary Figure Legends

Supplementary Figure 1

Supplementary Figure 2

Supplementary Figure 3

Supplementary Figure 4

Supplementary Figure 5

Supplementary Figure 6

Supplementary Figure 7

Supplementary Figure 8

Supplementary Figure 9

Supplementary Figure 10

Supplementary Figure 11

Supplementary Figure 12

Supplementary Figure 13

Original WB

Checklist

Agreement from all authors

Revised Manuscript_MarkedUp

## Data Availability

The datasets used and/or analyzed during the current study are available from the corresponding author upon reasonable request.
