# Supplementary material for: Modeling native and seeded Synuclein aggregation and related cellular dysfunctions in dopaminergic neurons derived by a new set of isogenic iPSC lines with SNCA multiplications
Source: Cell Death Dis. 2022 Oct 19;13(10):881. doi: 10.1038/s41419-022-05330-6 (PMC9581971; doi:10.1038/s41419-022-05330-6)
Supplement: Supplementary file 1 — Supplementary Figure Legends [file 41419_2022_5330_MOESM1_ESM.docx]

**Supplementary Figure Legends**

**Supplementary Figure 1:** (**A**) qPCR analysis of pluripotency genes in iPSCs compared to the corresponding human fibroblasts. (**B**) Karyotype analysis of iPSC lines displayed a normal euploid chromosome content. (**C**) MLPA analysis confirms the presence of the SNCA triplication. (**D**) Representative images and quantification of 4x*SNCA* NPCs show high level of αSyn compared to *SNCA*-KO and isogenic control neurons. Values are mean ± SEM. Dots indicate quantifications in 5 fields for 3 independent experiments. ***p < 0.001. Statistical analysis is performed using one-way ANOVA followed by Tukey post-test. Scale bars, 100 µm.

**Supplementary Figure 2:** (**A**) Schematic representation of the SNCA gene locus**.** (**B**) Tide analysis and Sanger sequencing of iPSCs line with 4x, 3x, 2x and 1x*SNCA* copies. (**C**) Schematic representation of two sgRNAs on exon 3 and exon 4 used to obtain the *SNCA*-KO iPSCs. (**D**) Image of PCR amplication used to select clone with deletion. (**E**) Tide analysis and Sanger sequencing confirms the deletion in the *SNCA*-KO iPSCs.

**Supplementary Figure 3:** (**A**) Differentiation protocol to generate DA neuronal cultures. (**B**) Representative images and quantification of regionalized midbrain NPCs stained for Nestin (red) and FOXA2 (green) in 4x, 3x, 2x, 1x*SNCA* and *SNCA*-KO midbrain NPCs. Values are mean ± SEM in 3 fields for 3 biological independent experiments. **p < 0.01, ***p < 0.001. Statistical analysis is performed using one-way ANOVA followed by Tukey post-test. Scale bars, 100 µm.

**Supplementary Figure 4:** Representative images of SNCA-KO**,** 2x and 4x*SNCA* neurons infected with the LV:Syn-GFP and stained for GFP and the pan-neuronal marker MAP2. Scale bars, 100 µm.

**Supplementary Figure 5:** Representative images and quantification of the FluoReSyn signal in 4x, 3x, 2x, 1x*SNCA* and *SNCA*-KO iPSC-derived DA neurons. Values are mean ± SEM. Dots represent quantification in 5 fields for 4 independent experiments. ***p < 0.001. Statistical analysis is performed using one-way ANOVA followed by Tukey post-test. Scale bars, 100 µm.

**Supplementary Figure 6:** Representative images and quantification of the Thioflavin S signal in 3 weeks old 4x, 3x, 2x, 1x*SNCA* and *SNCA*-KO DA neurons. Values are mean ± SEM of 4 biological independent experiments. ***p < 0.001. Statistical analysis is performed using one-way ANOVA followed by Tukey post-test. Scale bars, 100 µm.

**Supplementary Figure 7:** Representative images and quantification of 4x, 3x, 2x, 1x*SNCA* and *SNCA*-KO DA neurons co-stained for pS129αSyn and TH at 2, 3, 4 and 5 weeks of in vitro neuronal differentiation. Values are mean ± SEM. Dots indicate quantifications in 5 fields for 4 biological independent experiments. ***p < 0.001. Statistical analysis is performed using one-way ANOVA followed by Tukey post-test. Scale bars, 100 µm.

**Supplementary Figure 8:** Representative images of 4x, 3x, 2x, 1x*SNCA* and *SNCA*-KO mDA neurons immunostained for pS129αSyn (green) and total αSyn (red) showing the similar increase over time and relative co-distribution in neuronal cells (n=3 independent biological experiments). Scale bars, 100 µm.

**Supplementary Figure 9:** Representative images of 4x, 2x*SNCA* and *SNCA*-KO DA neurons immunostained for FluoReSyn-GFP signal (green) and pS129αSyn (red) to highlight their co-distribution in DA neuronal cultures. Signal corresponding to one αSyn inclusion is shown in the insets (n = 3 biological experiments). Scale bar, 100 µm.

**Supplementary Figure 10:** Super-resolved images showing the distribution of αSyn puncta and ATP5 positive mitochondrial figures and their relative proximity in 5 week old 4x*SNCA* DA neurons. Scale bars, 100 µm.

**Supplementary Figure 11:**(**A**) Representative images and quantification of the co-colocalization between mitochondria (Mitotracker green) and lysosomes (Lysotracker red) in 2x and 4x*SNCA* DA neurons. Dots indicate the number of cells analyzed (n = 30 somata). (**B**)  Representative images and quantification of the LC3-GFP signal in 2x, 4x*SNCA* and *SNCA*-KO DA neurons. Dots indicate quantifications in 5 fields for 3 independent experiments.  (**C**) Immunoblot for detecting LC3-I and LC3-II forms in 2x, 4x*SNCA* and *SNCA*-KO DA neuronal cultures. Values are mean ± SEM. ***p < 0.001. Statistical analysis is performed using one-way ANOVA followed by Tukey post-test. Scale bars, 100 µm.

**Supplementary Figure 12:** (**A**)Representative images of CLEM analysis in 4x*SNCA* DA neurons. Red arrows indicate the border of the αSYN inclusion. Green and light blue asterisks indicate abnormal mitochondria and autophagosomes, respectively. (**B**) Western blot analysis shows insoluble αSYN signal in 2 week old 4x*SNCA* treated with αSYN fibrils. (n = 3 independent experiments).

**Supplementary Figure 13:** (**A**) Schematic representation of TAXBP1 gene locus. (**B-C**) Sanger sequencing and Tide analysis confirm the nucleotide changes in the 4x*SNCA*;TAXBP1-KO iPSCs. (**D**) iPSC colonies immunostained for the pluripotency markers Oct4, NANOG and their morphology appearance of the 4x*SNCA*;TAXBP1-KO iPSC line. Scale bars, 100 µm.
