# Supplementary material for: Modeling native and seeded Synuclein aggregation and related cellular dysfunctions in dopaminergic neurons derived by a new set of isogenic iPSC lines with SNCA multiplications
Source: Cell Death Dis. 2022 Oct 19;13(10):881. doi: 10.1038/s41419-022-05330-6 (PMC9581971; doi:10.1038/s41419-022-05330-6)

WB αSyn (Figure 1)

αSyn (Syn211)

SNCA KO 1x 2x 3x 4x

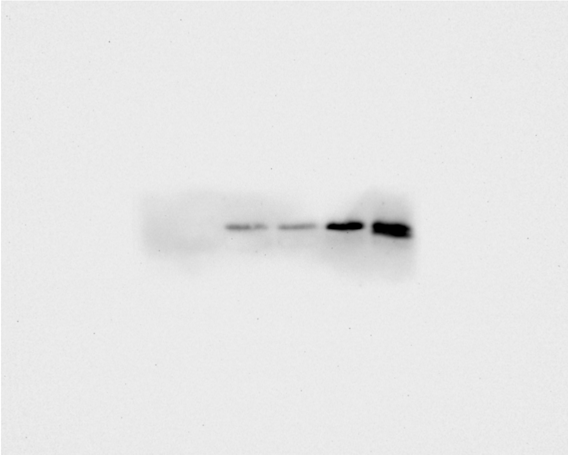

Actin

SNCA KO 1x 2x 3x 4x

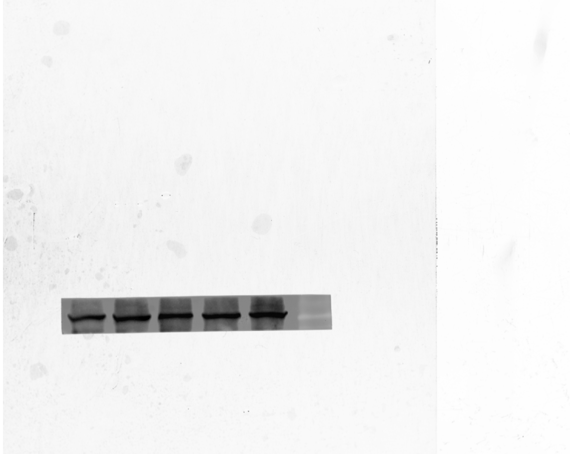

WB p62 (Figure 7)

p62

SNCA-KO 2xSNCA 4xSNCA 4xSNCA TAX1BP1-KO

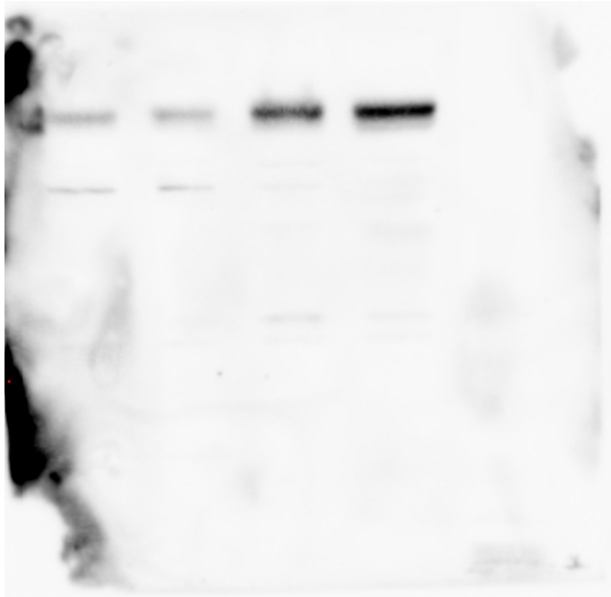

Calnexin

SNCA-KO 2xSNCA 4xSNCA 4xSNCA TAX1BP1-KO

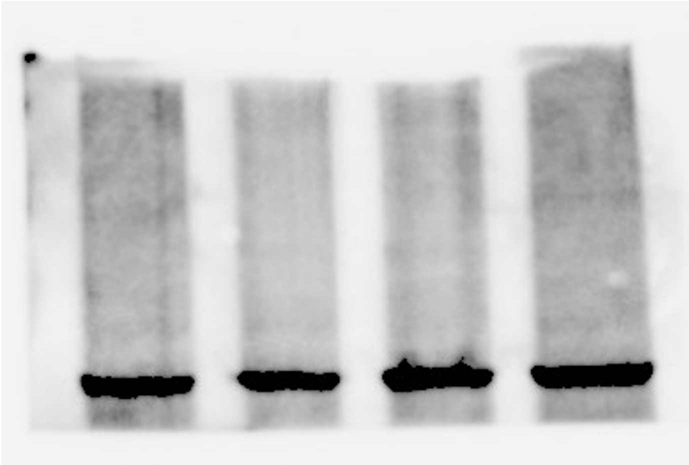

WB TAX1BP1 (Figure 7)

TAX1BP1

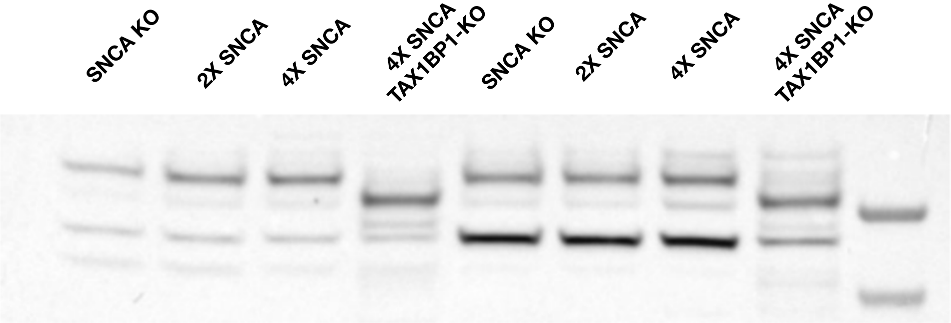

Actin

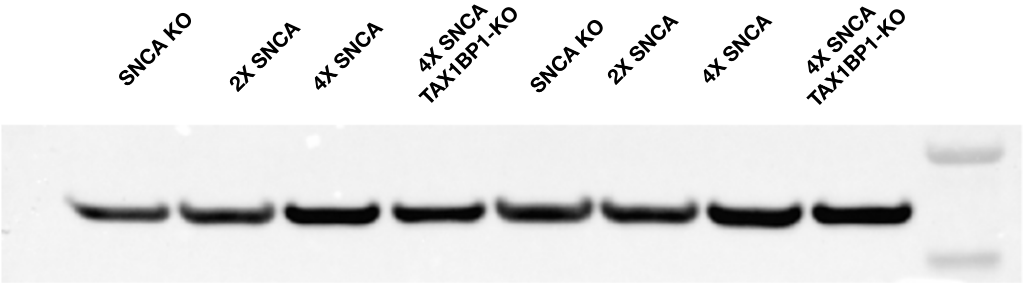

WB LC3 (Figure S11)

LC3

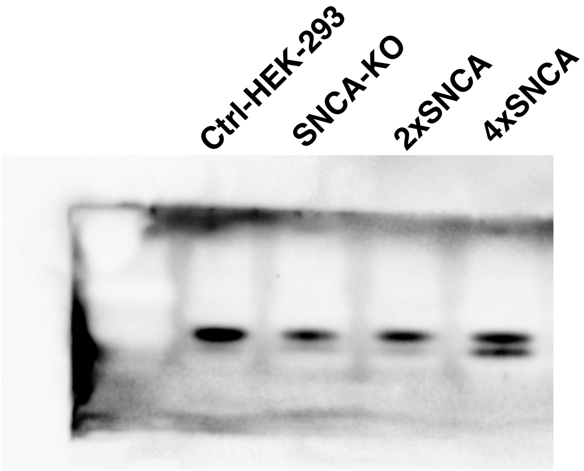

Calnexin

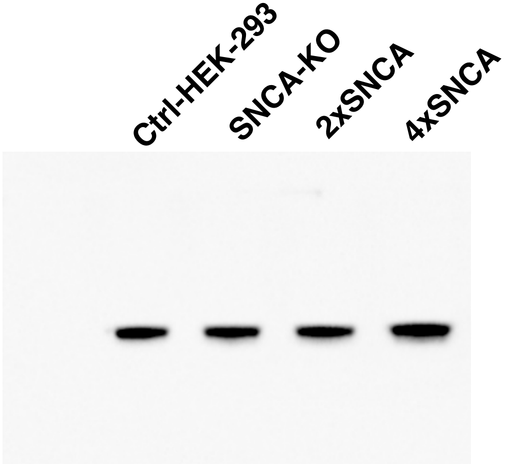

WB αSyn (Figure S12)

Soluble

αSyn (Syn211)

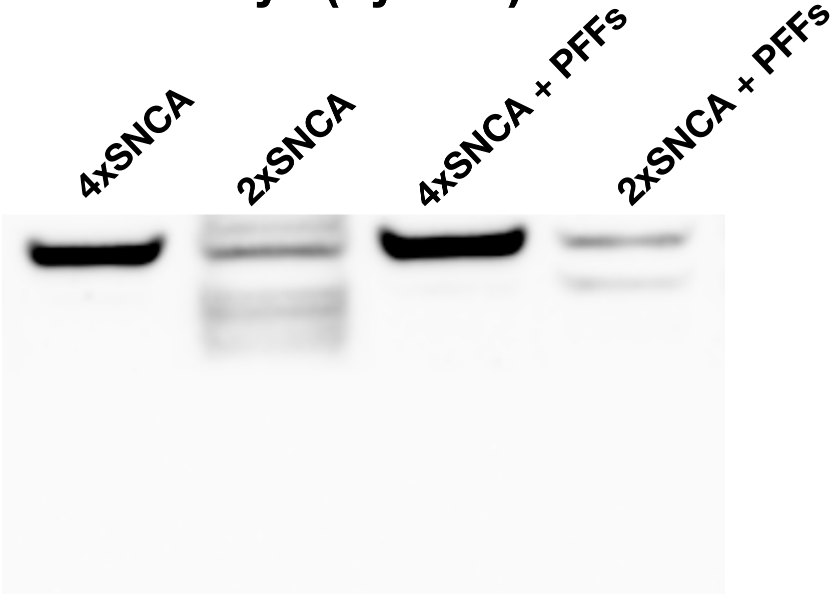

CLNX

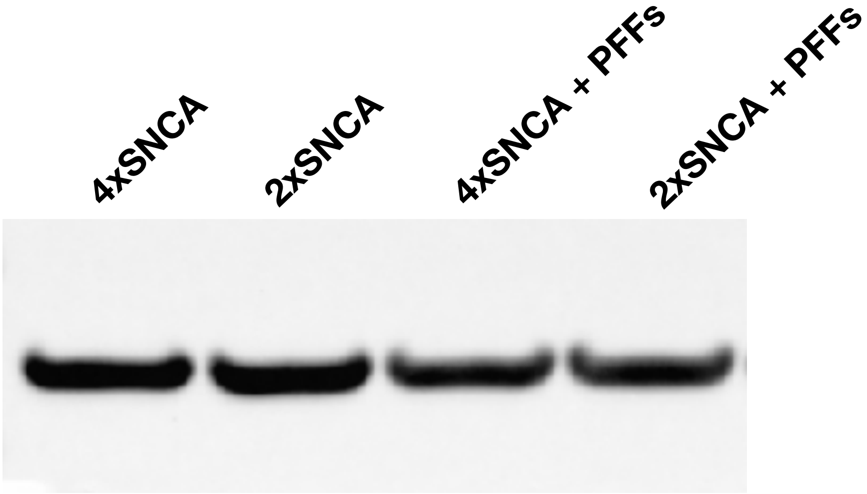

Insoluble

αSyn (Syn211)

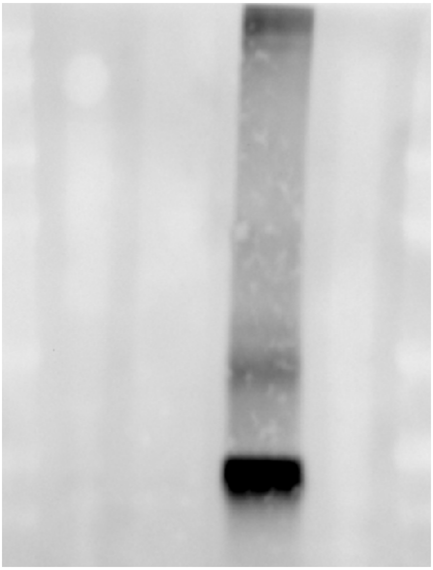

Supplement: Supplementary file 15 — Original WB [file 41419_2022_5330_MOESM15_ESM.pdf]
