# Supplementary material for: Modeling native and seeded Synuclein aggregation and related cellular dysfunctions in dopaminergic neurons derived by a new set of isogenic iPSC lines with SNCA multiplications
Source: Cell Death Dis. 2022 Oct 19;13(10):881. doi: 10.1038/s41419-022-05330-6 (PMC9581971; doi:10.1038/s41419-022-05330-6)
Supplement: Supplementary file 17 — Agreement from all authors [file 41419_2022_5330_MOESM17_ESM.pdf]

Da: **IANNIELLI ANGELO** iannielli.angelo@hsr.it
Oggetto: Re: CDDIS-22-0012RR Initial Quality Check
Data: 4 ottobre 2022, 18:45
A: Broccoli Vania broccoli.vania@hsr.it
Cc: LUONI MIRKO luoni.mirko@hsr.it, Giannelli Serena giannelli.serena@hsr.it, ferese.rosangela@gmail.com, Ordazzo Gabriele ordazzo.gabriele@hsr.it, Matteo Fossati matteo.fossati@in.cnr.it, RAIMONDI ANDREA raimondi.andrea@hsr.it, Felipe Opazo fopazo@gwdg.de, olga corti olga.corti@upmc.fr, Jochen Prehn JPrehn@rcsi.ie, Stefano Gambardella stefanogambardella@gmail.com, Ronald Melki ronald.melki@cnrs.fr

AI

Dear Vania,

I agree with the final version and authorship of the paper.

Angelo Iannielli

Stem Cells and Neurogenesis Unit
Division of Neuroscience
San Raffaele Scientific Institute
Via Olgettina 58, 20132 Milan, Italy

Phone: +39 022643 4612/4926
email: iannielli.angelo@hsr.it

www.vaniabroccolilab.com

Il giorno mar 4 ott 2022 alle ore 17:26 Broccoli Vania <broccoli.vania@hsr.it> ha scritto:

> Dear all,
>
> as you know our work on the iPSCs with SNCA multiplication have been provisionally accepted by CDDIS for publication.
> However, since the final version of the work includes 2 more authors, the Editorial Office requests a mail from each of you that you agree with the final authorship. I have attached below the final manuscript ready for publication.
>
> Please, send to me a mail agreeing with this autorship and then I will collect all the mails and forward them to the Editorial Office,
>
> Thanks so much for your collaboration!
> Vania
>
> Vania Broccoli
>
> CNR - National Research Council
> Institute of Neuroscience, Milan
> http://www.in.cnr.it/
> Email:vania.broccoli@cnr.it
>
> Head “Stem Cells and Neurogenesis” Unit
> Division of Neuroscience
> San Raffaele Scientific Institute
> Via Olgettina 58, 20132 Milan, Italy
> Tel. 02 26434616
> Email:broccoli.vania@hsr.it
>
> www.vaniabroccolilab.com
>
> > Inizio messaggio inoltrato:
> >
> > **Da:** cddisease@springernature.com
> > **Oggetto:** **CDDIS-22-0012RR Initial Quality Check**

Da: **LUONI MIRKO** luoni.mirko@hsr.it
Oggetto: Re: CDDIS-22-0012RR Initial Quality Check
Data: 4 ottobre 2022, 17:41
A: MELKI Ronald ronald.melki@cnrs.fr, Broccoli Vania broccoli.vania@hsr.it
Cc: IANNIELLI ANGELO iannielli.angelo@hsr.it, Giannelli Serena giannelli.serena@hsr.it, ferese.rosangela@gmail.com, Ordazzo Gabriele ordazzo.gabriele@hsr.it, Matteo Fossati matteo.fossati@in.cnr.it, RAIMONDI ANDREA raimondi.andrea@hsr.it, Felipe Opazo fopazo@gwdg.de, olga corti olga.corti@upmc.fr, Jochen Prehn JPrehn@rcsi.ie, Stefano Gambardella stefanogambardella@gmail.com

LM

Dead Vania,

I fully agree with the final authorship.

Mirko

Da: MELI Ronald <ronald.melki@cnrs.fr>
Inviato: martedì 4 ottobre 2022, 17:37
A: Broccoli Vania <broccoli.vania@hsr.it>
Cc: IANNIELLI ANGELO <iannielli.angelo@hsr.it>; LUONI MIRKO <luoni.mirko@hsr.it>; Giannelli Serena <giannelli.serena@hsr.it>; ferese.rosangela@gmail.com <ferese.rosangela@gmail.com>; Ordazzo Gabriele <ordazzo.gabriele@hsr.it>; Matteo Fossati <matteo.fossati@in.cnr.it>; RAIMONDI ANDREA <raimondi.andrea@hsr.it>; Felipe Opazo <fopazo@gwdg.de>; olga corti <olga.corti@upmc.fr>; Jochen Prehn <JPrehn@rcsi.ie>; Stefano Gambardella <stefanogambardella@gmail.com>
Oggetto: Re: CDDIS-22-0012RR Initial Quality Check

Drear Vania, Dear All,

I just learned the manuscript is accepted. Congratulations to all.

I fully agree with the final authorship. Please do forward my e-mail to the editorial office of Cell Death and Disease.

With my kind regards,

Ronald

Ronald Melki
Team Protein misfolding and aggregation in neurodegenerative diseases
https://jacob.cea.fr/drf/ifrancoisjacob/english/Pages/Departments/MIRCen/ResearchThemes/Protein-misfolding-aggregation-neurodegenerative-diseases.aspx
E-mail: ronald.melki@cnrs.fr
Tel: (33) 146549378 - Fax: (33) 146549116
Postal address: Institut Francois Jacob (MIRCen), CEA and Laboratory of Neurodegenerative Diseases, CNRS
18 Route du Panorama
92265 Fontenay-Aux-Roses cedex, France

> Le 4 oct. 2022 à 17:26, Broccoli Vania <broccoli.vania@hsr.it> a écrit :
>
> Dear all,
>
> as you know our work on the iPSCs with SNCA multiplication have been provisionally accepted by CDDIS for publication.
> However, since the final version of the work includes 2 more authors, the Editorial Office requests a mail from each of you that you agree with the final authorship. I have attached below the final manuscript ready for publication.
>
> Please, send to me a mail agreeing with this autorship and then I will collect all the mails and forward them to the Editorial Office,
>
> Thanks so much for your collaboration!
> Vania

Da: **Giannelli Serena** giannelli.serena@hsr.it
Oggetto: R: CDDIS-22-0012RR Initial Quality Check
Data: 4 ottobre 2022, 18:06
A: Broccoli Vania broccoli.vania@hsr.it, IANNIELLI ANGELO iannielli.angelo@hsr.it, LUONI MIRKO luoni.mirko@hsr.it, ferese.rosangela@gmail.com, Ordazzo Gabriele ordazzo.gabriele@hsr.it, Matteo Fossati matteo.fossati@in.cnr.it, RAIMONDI ANDREA raimondi.andrea@hsr.it, Felipe Opazo fopazo@gwdg.de, olga corti olga.corti@upmc.fr, Jochen Prehn JPrehn@rcsi.ie, Stefano Gambardella stefanogambardella@gmail.com, Ronald Melki ronald.melki@cnrs.fr

---

Dear Dr Broccoli,

I agree with the final version and authorship of the paper.

Serena Gea Giannelli
Stem Cells and Neurogenesis Unit
Ospedale San Raffaele
via Olgettina 60
20132 Milano, Italia
tel: 02-2643-5790 or 4612
e-mail: giannelli.serena@hsr.it

Da: Broccoli Vania <broccoli.vania@hsr.it>
Inviato: martedì 4 ottobre 2022 17:26
A: IANNIELLI ANGELO <iannielli.angelo@hsr.it>; LUONI MIRKO <luoni.mirko@hsr.it>; Giannelli Serena <giannelli.serena@hsr.it>; ferese.rosangela@gmail.com <ferese.rosangela@gmail.com>; Ordazzo Gabriele <ordazzo.gabriele@hsr.it>; Matteo Fossati <matteo.fossati@in.cnr.it>; RAIMONDI ANDREA <raimondi.andrea@hsr.it>; Felipe Opazo <fopazo@gwdg.de>; olga corti <olga.corti@upmc.fr>; Jochen Prehn <JPrehn@rcsi.ie>; Stefano Gambardella <stefanogambardella@gmail.com>; Ronald Melki <ronald.melki@cnrs.fr>
Oggetto: Fwd: CDDIS-22-0012RR Initial Quality Check

Dear all,

as you know our work on the iPSCs with SNCA multiplication have been provisionally accepted by CDDIS for publication.
However, since the final version of the work includes 2 more authors, the Editorial Office requests a mail from each of you that you agree with the final authorship. I have attached below the final manuscript ready for publication.

Please, send to me a mail agreeing with this autorship and then I will collect all the mails and forward them to the Editorial Office,

Thanks so much for your collaboration!
Vania

Vania Broccoli

CNR - National Research Council
Institute of Neuroscience, Milan
http://www.in.cnr.it/
Email:vania.broccoli@cnr.it

Head “Stem Cells and Neurogenesis” Unit
Division of Neuroscience
San Raffaele Scientific Institute
Via Olgettina 58, 20132 Milan, Italy
Tel. 02 26434616
Email:broccoli.vania@hsr.it

www.vaniabroccolilab.com

Da: **Rosangela Ferese** ferese.rosangela@gmail.com
Oggetto: Re: CDDIS-22-0012RR Initial Quality Check
Data: 5 ottobre 2022, 10:25
A: Broccoli Vania broccoli.vania@hsr.it
Cc: IANNIELLI ANGELO iannielli.angelo@hsr.it, LUONI MIRKO luoni.mirko@hsr.it, Giannelli Serena giannelli.serena@hsr.it, Ordazzo Gabriele ordazzo.gabriele@hsr.it, Matteo Fossati matteo.fossati@in.cnr.it, RAIMONDI ANDREA raimondi.andrea@hsr.it, Felipe Opazo fopazo@gwdg.de, olga corti olga.corti@upmc.fr, Jochen Prehn JPrehn@rcsi.ie, Stefano Gambardella stefanogambardella@gmail.com, Ronald Melki ronald.melki@cnrs.fr

RF

Dear Vania,

I agree with the final authorship and congratulations.

Rosangela

Dr. Rosangela Ferese, Ph.D.
Molecular Genetics Unit
IRCCS Neuromed Institute, INM
Parco Tecnologico
Via dell'Elettronica, c/da Camerelle
86077 Pozzilli (IS), Italy
Phone: +39 0865 915 379
Fax: +39 0865 927 575
Email: ferese.rosangela@gmail.com
rosangela.ferese@neuromed.it

The information contained in this message is confidential and is intended for the exclusive use of the person(s) or company(s) above addressed. If you are not the intended recipient(s), you are expressly prohibited from using, copying and distributing this message or any part of it in accordance with art. 616 of the criminal code and the law 196/03 (privacy). If you receive this message please don't read it but destroy it and then inform us by e-mail.

Respect the environment: if it's not necessary, don't print this mail.

Sostieni anche tu la ricerca nel campo delle malattie neurologiche: devolvi il 5x1000 del tuo reddito all'IRCCS Neuromed inserendo il codice fiscale 00068310945 nella casella " finanziamento agli enti di ricerca sanitaria ". Scopri di più su www.neuromed.it

Il giorno mar 4 ott 2022 alle ore 17:26 Broccoli Vania <broccoli.vania@hsr.it> ha scritto:

> Dear all,
>
> as you know our work on the iPSCs with SNCA multiplication have been provisionally accepted by CDDIS for publication.
> However, since the final version of the work includes 2 more authors, the Editorial Office requests a mail from each of you that you agree with the final authorship. I have attached below the final manuscript ready for publication.
>
> Please, send to me a mail agreeing with this autorship and then I will collect all the mails and forward them to the Editorial Office,
>
> Thanks so much for your collaboration!
> Vania

Da: **Ordazzo Gabriele** ordazzo.gabriele@hsr.it
Oggetto: Re: CDDIS-22-0012RR Initial Quality Check
Data: 4 ottobre 2022, 17:58
A: LUONI MIRKO luoni.mirko@hsr.it
Cc: MELKI Ronald ronald.melki@cnrs.fr, Broccoli Vania broccoli.vania@hsr.it, IANNIELLI ANGELO iannielli.angelo@hsr.it, Giannelli Serena giannelli.serena@hsr.it, ferese.rosangela@gmail.com, Matteo Fossati matteo.fossati@in.cnr.it, RAIMONDI ANDREA raimondi.andrea@hsr.it, Felipe Opazo fopazo@gwdg.de, olga corti olga.corti@upmc.fr, Jochen Prehn JPrehn@rcsi.ie, Stefano Gambardella stefanogambardella@gmail.com

OG

Dear Vania Broccoli,
I agree with the final version and authorship of the paper.

Gabriele Ordazzo

Stem Cells and Neurogenesis Unit

Division of Neuroscience

San Raffaele Scientific Institute

Via Olgettina 58, Milan, Italy

> Il giorno 4 ott 2022, alle ore 17:41, LUONI MIRKO <luoni.mirko@hsr.it> ha scritto:
>
> Dead Vania,
>
> I fully agree with the final authorship.
>
> Mirko
>
> ---
>
> **Da:** MELI Ronald <ronald.melki@cnrs.fr>
> **Inviato:** martedì 4 ottobre 2022, 17:37
> **A:** Broccoli Vania <broccoli.vania@hsr.it>
> **Cc:** IANNIELLI ANGELO <iannielli.angelo@hsr.it>; LUONI MIRKO <luoni.mirko@hsr.it>; Giannelli Serena <giannelli.serena@hsr.it>; ferese.rosangela@gmail.com <ferese.rosangela@gmail.com>; Ordazzo Gabriele <ordazzo.gabriele@hsr.it>; Matteo Fossati <matteo.fossati@in.cnr.it>; RAIMONDI ANDREA <raimondi.andrea@hsr.it>; Felipe Opazo <fopazo@gwdg.de>; olga corti <olga.corti@upmc.fr>; Jochen Prehn <JPrehn@rcsi.ie>; Stefano Gambardella <stefanogambardella@gmail.com>
> **Oggetto:** Re: CDDIS-22-0012RR Initial Quality Check
>
> Drear Vania, Dear All,
>
> I just learned the manuscript is accepted. Congratulations to all.
>
> I fully agree with the final authorship. Please do forward my e-mail to the editorial office of Cell Death and Disease.
>
> With my kind regards,
>
> Ronald
>
> Ronald Melki
> Team Protein misfolding and aggregation in neurodegenerative diseases
> https://jacob.cea.fr/drf/ifrancoisjacob/english/Pages/Departments/MIRCen/ResearchThemes/Protein-misfolding-aggregation-neurodegenerative-diseases.aspx
> E-mail: ronald.melki@cnrs.fr
> Tel: (33) 146549378 - Fax: (33) 146549116
> Postal address: Institut Francois Jacob (MIRCen), CEA and Laboratory of Neurodegenerative Diseases, CNRS
> 18 Route du Panorama
> 92265 Fontenay-Aux-Roses cedex, France

Da: **Matteo Fossati** matteo.fossati@in.cnr.it
Oggetto: Re: CDDIS-22-0012RR Initial Quality Check
Data: 5 ottobre 2022, 09:27
A: Broccoli Vania broccoli.vania@hsr.it

MF

Dear Vania,
I fully agree with the final version and authorship of the paper.
thanks

Matteo

--
Matteo Fossati, PhD
Head "Cell Biology of the Synapse" Lab
Institute of Neuroscience - CNR
Humanitas Research Hospital, Building E
via Rita Levi Montalcini 4
20072 - Pieve Emanuele (MI), Italy
Phone: +39 0282245251
Email: matteo.fossati@in.cnr.it
Email: matteo.fossati@humanitasresearch.it

Il giorno mar 4 ott 2022 alle ore 17:26 Broccoli Vania <broccoli.vania@hsr.it> ha scritto:

> Dear all,
>
> as you know our work on the iPSCs with SNCA multiplication have been provisionally accepted by CDDIS for publication.
> However, since the final version of the work includes 2 more authors, the Editorial Office requests a mail from each of you that you agree with the final authorship. I have attached below the final manuscript ready for publication.
>
> Please, send to me a mail agreeing with this autorship and then I will collect all the mails and forward them to the Editorial Office,
>
> Thanks so much for your collaboration!
> Vania
>
> Vania Broccoli
>
> CNR - National Research Council
> Institute of Neuroscience, Milan
> http://www.in.cnr.it/
> Email:vania.broccoli@cnr.it
>
> Head “Stem Cells and Neurogenesis” Unit
> Division of Neuroscience
> San Raffaele Scientific Institute
> Via Olgettina 58, 20132 Milan, Italy
> Tel. 02 26434616
> Email:broccoli.vania@hsr.it
>
> www.vaniabroccolilab.com
>
> > Inizio messaggio inoltrato:
> >
> > **Da:** cddisease@springernature.com
> > **Oggetto:** **CDDIS-22-0012RR Initial Quality Check**
> > **Data:** 4 ottobre 2022, 16:24:51 CEST
> > **A:** broccoli.vania@hsr.it
> > **Rispondi a:** cddisease@springernature.com
> >
> > Dear Professor Broccoli,
> >
> > In checking in your manuscript submitted to Cell Death & Disease it has come to our attention that the following

Da: **RAIMONDI ANDREA** raimondi.andrea@hsr.it
Oggetto: Re: CDDIS-22-0012RR Initial Quality Check
Data: 4 ottobre 2022, 22:24
A: Broccoli Vania broccoli.vania@hsr.it
Cc: IANNIELLI ANGELO iannielli.angelo@hsr.it, LUONI MIRKO luoni.mirko@hsr.it, Giannelli Serena giannelli.serena@hsr.it, ferese.rosangela@gmail.com, Ordazzo Gabriele ordazzo.gabriele@hsr.it, Matteo Fossati matteo.fossati@in.cnr.it, Felipe Opazo fopazo@gwdg.de, olga corti olga.corti@upmc.fr, Jochen Prehn JPrehn@rcsi.ie, Stefano Gambardella stefanogambardella@gmail.com, Ronald Melki ronald.melki@cnrs.fr

AR

Dear Vania,

I agree with the final authorship. Congratulations!

andrea

Il giorno mar 4 ott 2022 alle ore 17:26 Broccoli Vania <broccoli.vania@hsr.it> ha scritto:

> Dear all,
>
> as you know our work on the iPSCs with SNCA multiplication have been provisionally accepted by CDDIS for publication.
> However, since the final version of the work includes 2 more authors, the Editorial Office requests a mail from each of you that you agree with the final authorship. I have attached below the final manuscript ready for publication.
>
> Please, send to me a mail agreeing with this autorship and then I will collect all the mails and forward them to the Editorial Office,
>
> Thanks so much for your collaboration!
> Vania
>
> Vania Broccoli
>
> CNR - National Research Council
> Institute of Neuroscience, Milan
> http://www.in.cnr.it/
> Email:vania.broccoli@cnr.it
>
> Head “Stem Cells and Neurogenesis” Unit
> Division of Neuroscience
> San Raffaele Scientific Institute
> Via Olgettina 58, 20132 Milan, Italy
> Tel. 02 26434616
> Email:broccoli.vania@hsr.it
>
> www.vaniabroccolilab.com
>
>> Inizio messaggio inoltrato:
>>
>> **Da:** cddisease@springernature.com
>> **Oggetto:** **CDDIS-22-0012RR Initial Quality Check**
>> **Data:** 4 ottobre 2022, 16:24:51 CEST
>> **A:** broccoli.vania@hsr.it
>> **Rispondi a:** cddisease@springernature.com
>>
>> Dear Professor Broccoli,
>>
>> In checking in your manuscript submitted to Cell Death & Disease it has come to our attention that the following must be addressed before we can proceed.
>>
>> 1. It has come to our attention that your most recent author list differs from the one in your original submission. We find that the following authors have been added since your initial submission:
>>
>> Please request agreement from all authors including additions and deletions, these can be collected in the following way: Gabriele Ordazzo; Andrea Raimondi.

Da: **Opazo, Felipe** fopazo@gwdg.de
Oggetto: Re: CDDIS-22-0012RR Initial Quality Check
Data: 4 ottobre 2022, 17:38
A: Broccoli Vania broccoli.vania@hsr.it

FO

Dear Vania,

Many thanks for your email and the great news.
I agree with the last modified list of authors (13x authors).
All the best,
Felipe

Dr. Felipe Opazo
Molecular Probes for Quantitative Neurosciences
Center for Biostructural Imaging of Neurodegeneration (BIN)
von-Siebold-Straße 3a
37075 Göttingen
Germany
Tel.: +49 (0)551 / 39-61156
email.: fopazo@gwdg.de
web.: https://opazolab.de

> On 4. Oct 2022, at 17:26, Broccoli Vania <broccoli.vania@hsr.it> wrote:
>
> Dear all,
>
> as you know our work on the iPSCs with SNCA multiplication have been provisionally accepted by CDDIS for publication.
> However, since the final version of the work includes 2 more authors, the Editorial Office requests a mail from each of you that you agree with the final authorship. I have attached below the final manuscript ready for publication.
>
> Please, send to me a mail agreeing with this autorship and then I will collect all the mails and forward them to the Editorial Office,
>
> Thanks so much for your collaboration!
> Vania
>
> Vania Broccoli
>
> CNR - National Research Council
> Institute of Neuroscience, Milan
> http://www.in.cnr.it/
> Email:vania.broccoli@cnr.it
>
> Head “Stem Cells and Neurogenesis” Unit
> Division of Neuroscience
> San Raffaele Scientific Institute
> Via Olgettina 58, 20132 Milan, Italy
> Tel. 02 26434616
> Email:broccoli.vania@hsr.it
>
> www.vaniabroccolilab.com
>
>> Inizio messaggio inoltrato:
>>
>> **Da:** cddisease@springernature.com
>> **Oggetto: CDDIS-22-0012RR Initial Quality Check**
>> **Data:** 4 ottobre 2022, 16:24:51 CEST
>> **A:** broccoli.vania@hsr.it
>> **Rispondi a:** cddisease@springernature.com
>>
>> Dear Professor Broccoli

Da: **CORTI Olga** olga.corti@icm-institute.org
Oggetto: Re: CDDIS-22-0012RR Initial Quality Check
Data: 5 ottobre 2022, 10:04
A: Broccoli Vania broccoli.vania@hsr.it, IANNIELLI ANGELO iannielli.angelo@hsr.it, LUONI MIRKO luoni.mirko@hsr.it, Giannelli Serena giannelli.serena@hsr.it, ferese.rosangela@gmail.com, Ordazzo Gabriele ordazzo.gabriele@hsr.it, Matteo Fossati matteo.fossati@in.cnr.it, RAIMONDI ANDREA raimondi.andrea@hsr.it, Felipe Opazo fopazo@gwdg.de, CORTI Olga olga.corti@upmc.fr, Jochen Prehn JPrehn@rcsi.ie, Stefano Gambardella stefanogambardella@gmail.com, Ronald Melki ronald.melki@cnrs.fr

CO

Dear Vania,
Congratulations !
I agree with the final authorship of the manuscript. Thanks for sending my mail to the Editorial Office of CDDIS.
All the best,
Olga

Olga Corti

Physiopathologie Moléculaire de la maladie de Parkinson/Molecular Pathophysiology of Parkinson's disease
https://icm-institute.org/en/team/team-corti-corvol/
Institut du Cerveau – Paris Brain Institute
CNRS UMR 7225 – INSERM U 1127 – UPMC-P6 UMR S 1127
Hôpital de la Pitié-Salpêtrière
47, boulevard de l'Hôpital
75013 Paris, France
Tél/Phone : +33 (0)1 57 27 46 51

De : Broccoli Vania <broccoli.vania@hsr.it>
Date : mercredi, 5 octobre 2022 à 05:13
À : IANNIELLI ANGELO <iannielli.angelo@hsr.it>, LUONI MIRKO <luoni.mirko@hsr.it>, Giannelli Serena <giannelli.serena@hsr.it>, ferese.rosangela@gmail.com <ferese.rosangela@gmail.com>, Ordazzo Gabriele <ordazzo.gabriele@hsr.it>, Matteo Fossati <matteo.fossati@in.cnr.it>, RAIMONDI ANDREA <raimondi.andrea@hsr.it>, Felipe Opazo <fopazo@gwdg.de>, CORTI Olga <olga.corti@upmc.fr>, Jochen Prehn <JPrehn@rcsi.ie>, Stefano Gambardella <stefanogambardella@gmail.com>, Ronald Melki <ronald.melki@cnrs.fr>
Objet : Fwd: CDDIS-22-0012RR Initial Quality Check

Dear all,

as you know our work on the iPSCs with SNCA multiplication have been provisionally accepted by CDDIS for publication.
However, since the final version of the work includes 2 more authors, the Editorial Office requests a mail from each of you that you agree with the final authorship. I have attached below the final manuscript ready for publication.

Please, send to me a mail agreeing with this autorship and then I will collect all the

Da: **Jochen Prehn** JPrehn@rcsi.ie
Oggetto: Re: CDDIS-22-0012RR Initial Quality Check
Data: 4 ottobre 2022, 19:31
A: Broccoli Vania broccoli.vania@hsr.it
Cc: IANNIELLI ANGELO iannielli.angelo@hsr.it, LUONI MIRKO luoni.mirko@hsr.it, Giannelli Serena giannelli.serena@hsr.it, ferese.rosangela@gmail.com, Ordazzo Gabriele ordazzo.gabriele@hsr.it, Matteo Fossati matteo.fossati@in.cnr.it, RAIMONDI ANDREA raimondi.andrea@hsr.it, Felipe Opazo fopazo@gwdg.de, olga corti olga.corti@upmc.fr, Stefano Gambardella stefanogambardella@gmail.com, Ronald Melki ronald.melki@cnrs.fr

JP

Thanks Vania

I am also agreeing to the final authorship

Kind regards
Jochen

Prof. Jochen Prehn He/Him
Professor of Physiology & Director, Centre for Systems Medicine

RCSI UNIVERSITY OF MEDICINE AND HEALTH SCIENCES

Physiology & Medical Physics
123 St. Stephen's Green Dublin 2 Ireland
T: 01-402-2255 **F:** 01-402-2261
E: JPrehn@rcsi.ie **W:** www.rcsi.com

*Discover why we are Top 50 in the World for 'International Outlook' - **THE** World University Ranking 2022*

WORLD TOP 50 for GOOD HEALTH and WELL-BEING THE IMPACT RANKINGS 2022

Athena SWAN Bronze Award ECU GENDER CHARTER

Accredited in Workplace Wellbeing An Ibec initiative The KeepWell Mark

If you receive this email outside of your normal working hours, you are not expected to respond until you return to work.

Registered Charity Number: 20001957

> On 4 Oct 2022, at 16:27, Broccoli Vania <broccoli.vania@hsr.it> wrote:
>
> CAUTION: This email originated from outside of the organisation. Do not click links or open attachments unless you recognise the sender and know that the content is safe.
>
> Dear all,

Da: **Stefano Gambardella** stefanogambardella@gmail.com
Oggetto: Re: CDDIS-22-0012RR Initial Quality Check
Data: 4 ottobre 2022, 18:25
A: Broccoli Vania broccoli.vania@hsr.it

SG

Dead Vania,

I fully agree with the final authorship

Thanks
Stefano

Il mar 4 ott 2022, 17:26 Broccoli Vania <broccoli.vania@hsr.it> ha scritto:

> Dear all,
>
> as you know our work on the iPSCs with SNCA multiplication have been provisionally accepted by CDDIS for publication.
> However, since the final version of the work includes 2 more authors, the Editorial Office requests a mail from each of you that you agree with the final authorship. I have attached below the final manuscript ready for publication.
>
> Please, send to me a mail agreeing with this autorship and then I will collect all the mails and forward them to the Editorial Office,
>
> Thanks so much for your collaboration!
> Vania
>
> Vania Broccoli
>
> CNR - National Research Council
> Institute of Neuroscience, Milan
> http://www.in.cnr.it/
> Email:vania.broccoli@cnr.it
>
> Head "Stem Cells and Neurogenesis" Unit
> Division of Neuroscience
> San Raffaele Scientific Institute
> Via Olgettina 58, 20132 Milan, Italy
> Tel. 02 26434616
> Email:broccoli.vania@hsr.it
>
> www.vaniabroccolilab.com
>
>> Inizio messaggio inoltrato:
>>
>> **Da:** cddisease@springernature.com
>> **Oggetto:** **CDDIS-22-0012RR Initial Quality Check**
>> **Data:** 4 ottobre 2022, 16:24:51 CEST
>> **A:** broccoli.vania@hsr.it
>> **Rispondi a:** cddisease@springernature.com
>>
>> Dear Professor Broccoli,
>>
>> In checking in your manuscript submitted to Cell Death & Disease it has come to our attention that the following must be addressed before we can proceed.
>>
>> 1. It has come to our attention that your most recent author list differs from the one in your original submission. We find that the following authors have been added since your initial submission:
>>
>> Please request agreement from all authors including additions and deletions, these can be collected in the following way: Gabriele Ordazzo; Andrea Raimondi.
>>
>> Email your co-authors with the change, and ask them to reply to your email confirming that they agree to these changes. Once you have collected these replies, please combine all of the co-authors' email responses in one [illegible]

Da: **MELKI Ronald** Ronald.MELKI@cnrs.fr
Oggetto: Re: CDDIS-22-0012RR Initial Quality Check
Data: 4 ottobre 2022, 17:37
A: Broccoli Vania broccoli.vania@hsr.it
Cc: IANNIELLI ANGELO iannielli.angelo@hsr.it, LUONI MIRKO luoni.mirko@hsr.it, Giannelli Serena giannelli.serena@hsr.it, ferese.rosangela@gmail.com, Ordazzo Gabriele ordazzo.gabriele@hsr.it, Matteo Fossati matteo.fossati@in.cnr.it, RAIMONDI ANDREA raimondi.andrea@hsr.it, Felipe Opazo fopazo@gwdg.de, olga corti olga.corti@upmc.fr, Jochen Prehn JPrehn@rcsi.ie, Stefano Gambardella stefanogambardella@gmail.com

MR

Drear Vania, Dear All,

I just learned the manuscript is accepted. Congratulations to all.

I fully agree with the final authorship. Please do forward my e-mail to the editorial office of Cell Death and Disease.

With my kind regards,

Ronald

Ronald Melki
Team Protein misfolding and aggregation in neurodegenerative diseases
https://jacob.cea.fr/drf/ifrancoisjacob/english/Pages/Departments/MIRCen/ResearchThemes/Protein-misfolding-aggregation-neurodegenerative-diseases.aspx
E-mail: ronald.melki@cnrs.fr
Tel: (33) 146549378 - Fax: (33) 146549116
Postal address: Institut Francois Jacob (MIRCen), CEA and Laboratory of Neurodegenerative Diseases, CNRS
18 Route du Panorama
92265 Fontenay-Aux-Roses cedex, France

> Le 4 oct. 2022 à 17:26, Broccoli Vania <broccoli.vania@hsr.it> a écrit :
>
> Dear all,
>
> as you know our work on the iPSCs with SNCA multiplication have been provisionally accepted by CDDIS for publication.
> However, since the final version of the work includes 2 more authors, the Editorial Office requests a mail from each of you that you agree with the final authorship. I have attached below the final manuscript ready for publication.
>
> Please, send to me a mail agreeing with this autorship and then I will collect all the mails and forward them to the Editorial Office,
>
> Thanks so much for your collaboration!
> Vania
>
>
> Vania Broccoli
>
> CNR - National Research Council
> Institute of Neuroscience, Milan
> http://www.in.cnr.it/
> Email:vania.broccoli@cnr.it
>
> Head “Stem Cells and Neurogenesis” Unit
> Division of Neuroscience
> San Raffaele Scientific Institute
> Via Olgettina 58, 20132 Milan, Italy
> Tel. 02 26434616
> Email:broccoli.vania@hsr.it
>
> www.vaniabroccolilab.com
>
>
>> Inizio messaggio inoltrato:
>>
>> **Da:** cddisease@springernature.com
