# Supplementary material for: Modeling native and seeded Synuclein aggregation and related cellular dysfunctions in dopaminergic neurons derived by a new set of isogenic iPSC lines with SNCA multiplications
Source: Cell Death Dis. 2022 Oct 19;13(10):881. doi: 10.1038/s41419-022-05330-6 (PMC9581971; doi:10.1038/s41419-022-05330-6)
Supplement: Supplementary file 18 — Revised Manuscript_MarkedUp [file 41419_2022_5330_MOESM18_ESM.docx]

**Modeling native and seeded Synuclein aggregation and related cellular dysfunctions in dopaminergic neurons derived by a new set of isogenic iPSC lines with SNCA multiplications**

Angelo Iannielli^1,2*^, Mirko Luoni^2*^, Serena Gea Giannelli^2*^, Rosangela Ferese^3^, Gabriele Ordazzo^2^, Matteo Fossati^1,4^, Andrea Raimondi^5^, Felipe Opazo^6^, Olga Corti^7^, Jochen H.M. Prehn^8^, Stefano Gambardella^3,9^, Ronald Melki^10^, Vania Broccoli^1,2^

^1^National Research Council (CNR), Institute of Neuroscience, 20129 Milan, Italy; ^2^Division of Neuroscience, San Raffaele Scientific Institute, 20132 Milan, Italy; ^3^IRCCS Neuromed, Pozzilli, Italy; ^4^IRCCS Humanitas Research Hospital, via Manzoni 56, 20089 Rozzano, Milan, Italy; ^5^Experimental Imaging Center, San Raffaele Scientific Institute, Milan, Italy; ^6^University Medical Center Göttingen, D-37073 Göttingen, Germany; ^7^Sorbonne Université, Institut du Cerveau (ICM), Inserm U1127, CNRS UMR 7225, Paris, France. ^8^Royal College of Surgeons in Ireland University of Medicine and Health Sciences, Department of Physiology and Medical Physics and SFI FutureNeuro Research Centre, 123 St. Stephen's Green, Dublin, Ireland; ^9^Department of Biomolecular Sciences, University of Urbino "Carlo Bo," Urbino, Italy; ^10^Institut Francois Jacob, Molecular Imaging Center (MIRCen), Commissariat à l'Energie Atomique et aux Energies Alternatives (CEA) and Centre National de la Recherche Scientifique (CNRS), Université Paris-Saclay, Fontenay-aux-Roses, France.

*These authors contributed equally

**Running title:** Synuclein aggregation in isogenic iPSC lines with SNCA multiplications

**Corresponding author:**

**Vania Broccoli**

Stem Cells and Neurogenesis Unit, Division of Neuroscience, San Raffaele Scientific Institute, Via Olgettina 58, 20132 Milan, Italy. Tel: +39 02 26434616; FAX +39 02 26434621.

E-mail: broccoli.vania@hsr.it. Website: *www.vaniabroccolilab.com*

**Abstract**

**Triplication of the *SNCA* gene, encoding the protein alpha-Synuclein (αSyn), is a rare cause of aggressive and early-onset parkinsonism. Herein, we generated iPSCs from two siblings with a recently described compact *SNCA* gene triplication and suffering for severe motor impairments, psychiatric symptoms and cognitive deterioration. Using CRISPR/Cas9 gene editing, each *SNCA* copy was inactivated by targeted indel mutations generating a panel of isogenic iPSCs with decremental number from 4 down to none of functional *SNCA* gene alleles. We differentiated these iPSC lines in midbrain dopaminergic (DA) neuronal cultures to characterize αSyn aggregation in native and seeded conditions and evaluating its associated cellular dysfunctions. Utilizing a new nanobody-based biosensor combined with super-resolved imaging, we were able to visualize and measure αSyn aggregates in early DA neurons in unstimulated conditions. Calcium dysregulation and mitochondrial alterations were the first pathological signs detectable in early differentiated DA neuronal cultures. Accelerated αSyn aggregation was induced by exposing neurons to structurally well-characterized synthetic αSyn fibrils. 4x*SNCA* DA neurons showed the highest vulnerability which was associated with high levels of oxidized DA and amplified by TAX1BP1 gene disruption. Seeded DA neurons developed large αSyn deposits whose morphology and internal constituents resembled Lewy bodies commonly observed in Parkinson’s disease (PD) patient brain tissues. These findings provide strong evidence that this isogenic panel of iPSCs with *SNCA* multiplications offers a remarkable cellular platform to investigate mechanisms of PD and validate candidate inhibitors of native and seeded αSyn aggregation.**

**Introduction**

Although Parkinson’s disease (PD) is a neurodegenerative disorder which commonly is manifested in idiopathic forms, about 5% of cases are familiar and caused by pathological alterations in single genes (1-3). The alpha-Synuclein (αSyn) encoding gene *SNCA* was the first genetic locus identified with a missense mutation responsible for a dominant genetic form of PD originally described in the large Contursi kindred (4). Importantly, αSyn protein aggregates are one of the major constituents of Lewy Bodies, cytoplasmic inclusions predominantly present in neurons of autopsy-derived PD brain tissues (5). Subsequent genetic studies identified additional pathogenetic alterations in *SNCA*, including other point mutations and multiplications in the form of duplications and triplications (6-8). Although *SNCA* duplications are relatively common in inherited dominant PD forms, missense mutations and triplications are extremely rare (9). Patients with three *SNCA* copies manifest comparable disease onset and progression with those described in idiopathic forms, although they are more likely to develop cognitive decline and sleep disturbance (8). In contrast, patients with *SNCA* triplication develop an aggressive and early-onset parkinsonism, combined with additional non-motor signs, such as cognitive dysfunctions with features reminiscent of dementia with Lewy bodies (9,10). These clinical presentations suggest that *SNCA* triplications cause a more severe phenotype than *SNCA* duplications, consistently with the underlying genetic defects. Thus, PD patients with four *SNCA* copies offer a unique opportunity to unveil the underlying pathophysiological mechanisms directly triggered by increased *SNCA* gene dosage. With this in mind, iPSC technology offers a powerful system to generate and characterize patient-specific cells affected by the disease. Recent studies have convincingly reported a variety of pathophysiological defects exhibited by neural cells with *SNCA* triplication such as increased endoplasmic reticulum stress, altered lysosomal and mitochondrial functions, dysregulated autophagy and heightened oxidative stress levels (11-17). However, these pathological alterations have been described in neurons after advanced time in culture while the exact early dysfunctions and chain of pathological events remains to be fully elucidated. In addition, the available iPSCs with *SNCA* triplication have been originally derived by only two patients and most of the cell lines were reprogrammed with integrating retroviruses (16-18). Thus, it is imperative to produce additional iPSC lines with non-integranting methodologies from more patients and develop isogenic controls for a more stringent characterization. Moreover, the availability of iPSCs from patients with different clinical presentations might offer new opportunities for identifying disease gene modifier by candidate analysis or unbiased screens. Recently, an additional pedigree with dominant inheritance of a multiplication of *SNCA* has been reported that was characterized by four copies of *SNCA* three of which derived from a triplicated region of 351Kb containing *SNCA*, and a duplication of a genomic region flanking the triplication (19,20). The two siblings (brother and sister) analyzed in these studies showed rapid motor deterioration, cognitive and psychiatric symptoms and sleep disturbances. However, the brother showed earlier onset compared to the sister (age 28 and 42, respectively) and significant faster progression of both motor and non-motor clinical presentations. Thus, although *SNCA* triplication causes a highly penetrant and aggressive PD form, time and severity of symptom presentations can vary requiring a deep and longitudinal clinical assessment to classify the diverse phenotypes. Similar symptomatic differences have been extrapolated among PD patients belonging to 8 different families with *SNCA* triplication inheritance (8). Herein, we generated iPSCs from the two siblings described above and produced a panel of isogenic iPSCs by targeted inactivation of each of the SNCA copies by CRISPR/Cas9 gene editing up to obtaining isogenic SNCA knock-out cells. These iPSCs were differentiated into DA neuronal cultures to characterize native and seeded αSyn aggregation with a new biosensor and correlate αSyn aggregation with cellular dysfunctions and neuronal survival.

**Results**

**Generation of a panel of isogenic iPSC lines with a novel SNCA multiplication**

Given the uniqueness of this *SNCA* multiplication and its association with a particular severe and fast progressing PD pathological phenotype, we decided to derive iPSC lines through cell reprogramming. To achieve this goal, peripheral blood withdrawals from both siblings were obtained from isolated mononuclear cells (PBMCs) that were reprogrammed into iPSCs with a non-integrating system based on Sendai viruses expressing the Yamanaka’s genes (CytoTune^TM^-iPS). iPSC lines were initially selected based on the correct colony morphology, normal karyotype, expression of crucial pluripotency genes and silencing of fibroblast-specific genes (**Figures 1A; S1A,B**). Then, two independent lines for each of the siblings (brother: iPSC-3.3A,B and sister: iPSC-3.2A,B) were assessed for the presence of the *SNCA* triplication that was confirmed through MLPA analysis (**Figure S1C**). Next, the four selected iPSC lines were induced into neural progenitor cells (NPCs) following a consolidated *in vitro* differentiation protocol (21). While the iPSC-3.2A,B lines derived from the sister consistently generated highly proliferating and homogeneous NPC cultures, the brother’s iPSC-3.3A,B lines gave rise to highly unstable NPC cultures with poor differentiation capability (data not shown). These alterations in iPSCs are in accordance with the relative different severity of symptoms manifested by the two siblings with the brother displaying a significant earlier onset and faster disease progression with respect to the affected sister (19). Therefore, these results indicate that unknown genetic modifiers can substantially modulate the outcome of the disease phenotype despite the high penetrance of the genetic *SNCA* triplication. Despite our interest on this aspect, we decided to focus this study exclusively on the analysis of the sister’s iPSC-3.2 lines and their derivatives. In order to generate isogenic controls, we employed CRISPR/Cas9 technology to inactivate each of the four *SNCA* alleles in the 4x*SNCA* iPSC-3.2A line (henceforth cited only as 4x*SNCA*). To do so, we validated one sgRNA with an efficient and selective targeting of the *SNCA* exon 3 encoding for the N-terminal amphipathic region (**Figure 1A**). After simultaneous transfection of sgRNA-Ex3 and spCas9 expressing plasmids and transient selection with antibiotics, we isolated and screened primary clones for the desired modifications. Through TIDE analysis of the PCR product combined with Sanger sequencing of at least 40 amplicons we selected iPSCs lines where either one, two or three *SNCA* alleles were targeted with frameshift-inducing indel mutations, generating the 3x, 2x and 1x*SNCA* iPSC lines, respectively **(Figure 1A, S2)**. However, we were unable to obtain a clone with all four *SNCA* alleles inactivated. Thus, we designed a second sgRNA on *SNCA* exon 4 and repeated the CRISPR/Cas9 gene editing with the same 4x*SNCA* line combining this time the two exon 3 and 4 sgRNAs. By genomic PCR amplification we selected iPSC clones with evident deletion between *SNCA* exons 4 and 3 followed by TIDE analysis and amplicon sequencing to detect indels mutations in either exon. After this analysis, we were able to isolate *SNCA*-KO iPSCs where all four *SNCA* alleles were either deleted or inactivated by indel mutations **(Figure S2)**. To confirm the functional effects of the Cas9-induced indel mutations, we analyzed αSyn protein levels in NPCs, since undifferentiated iPSCs have negligible levels of αSyn expression. Intriguingly, the total amount of αSyn protein was strictly correlated to the number of unmodified *SNCA* alleles, with the patient 4x*SNCA* NPCs having the highest abundance while the SNCA-KO NPCs showing no detectable trace of αSyn protein **(Figures 1B)**. These results were corroborated by immunohistochemistry on iPSC-derived neurons where the intensity of the immunostaining signal between the different lines was correlated to the number of unmodified and functional *SNCA* alleles **(Figure S1D)**. In addition, analysis of extracellular αSyn secreted into the culture medium by ELISA assay revealed marked increased αSyn release in 4x*SNCA* NPCs **(Figure 1C)**. Thus, we selected two iPSC lines each for 4x*SNCA* (parental and unmodified iPSC-3.2A lines), 2x*SNCA* (clones #22, #29) and *SNCA*-KO (clones #40, #51) that showed stable clone morphology and growth, normal karyotype, high expression of the pluripotent genes and highly efficient neuronal differentiation for the subsequent functional analysis **(Figures 1D,S1A)**.

**Cytoplasmic αSyn assemblies in iPSC-derived DA neuronal cultures in basal conditions**

Given the general high abundance of αSyn protein in 4x*SNCA* iPSCs, we wondered whether these cells would be prone to develop signs of αSyn aggregation in unstimulated conditions. Hence, iPSCs were induced into midbrain DA neurons following a standard neural midbrain floor-plate protocol which included the generation of homogeneous cultures of DA neural progenitors as an intermediate step during the cell differentiation process (**Figure S3**). All iPSC lines showed a very high neuronal differentiation efficiency with a large fraction of MAP2^+^ neurons expressing Tyrosine Hydroxylase (TH) (4x*SNCA*: 67% TH^+^/MAP2^+^; 2x*SNCA*: 71% TH^+^/MAP2^+^; *SNCA*-KO: 74% TH^+^/MAP2^+^) (**Figures 1,S3**). To visualize αSyn oligomers in early generated neurons we decided to combine fluorescent reporter staining with super-resolution microscopy. Initially, we generated both lentiviruses (LVs) and Adeno-Associated Viruses (AAVs) expressing a GFP-tagged SNCA that could bind to nascent αSyn assemblies and, thereby, preferentially highlight cytoplasmic aggregates. However, with both viruses GFP expression appeared very homogeneous throughout the cytoplasm of neurons failing to discriminate any αSyn aggregates (**Figure S4** and data not shown). Next, we decided to exploit the nanobody-derived fluorescent reporter FluoReSyn recently generated by Gerdes and colleagues (22), which relies on the specific and selective binding of a nanobody to the C-terminal region of αSyn. Interestingly, FluoReSyn has a second binding to the 20S proteasome subunit Rpn10 which stimulates its degradation in absence of αSyn binding. Thus, FluoReSyn has an excellent background-to-signal ratio representing a valuable biosensor to detect αSyn protein. We reasoned that FluoReSyn could be further exploited to highlight intra-cytoplasmatic αSyn assemblies thanks to its small size, stability and low background. Thus, we packaged FluoReSyn into an AAV-PHP.B (AAV-FluoReSyn) to transduce 1 week old iPSC-derived DA neurons before processing them for STED3X and Lattice SIM^2^ super-resolution microscopy with a resolution below 50 nm (**Figure 2A**). Intriguingly, the FluoReSyn signal was easily detectable after immunocytochemical staining revealing significantly more positive puncta in 4x*SNCA* when compared to isogenic control 2x*SNCA* DA neurons (**Figure 2B**). Importantly, FluoReSyn staining was undetectable in *SNCA*-KO neuronal cultures confirming the specificity and low background of this biosensor (**Figure 2B**). Quantitative analysis revealed that 4x*SNCA* neurons developed a more diffuse accumulation of αSyn aggregates per neuronal somata (4x*SNCA*: *94* ± 14; 2x*SNCA: 37* ± 8; *SNCA*-KO: *4* ± 3)*,* with larger average size compared to isogenic control DA neurons (4x*SNCA*: 112 nm ± 18; 2x*SNCA:* 46 nm ± 9 in average) (**Figures 2C,D**). Next, we stained DA neuronal cultures with Thioflavin S, a dye commonly utilized to detect mature protein aggregates of amyloid nature. Differently from FluoReSyn, Thioflavin S was not sensitive enough to highlight small aggregates in 2 weeks old DA neurons. However, Thioflavin S positive puncta were detectable in 5 weeks old 4x*SNCA* neurons indicating a progressive accumulation of αSyn aggregates during neuronal maturation *in vitro* (**Figure 2E**). Intriguingly, the FluoReSyn signal increased proportionally with Thioflavin S and Serine 129 phosphorylated αSyn (pS129αSYN) staining in neuronal cultures of iPSCs with additional *SNCA* copy number and over time in culture (**Figures S5-S8**). Moreover, direct side-by-side staining revealed that pS129αSYN positive puncta for the were virtually always stained with the FluoReSyn biosensor (**Figure S9**).

Given the well-described interactions between αSyn aggregates and mitochondria, we assessed their physical proximity on 4x*SNCA* neuronal cultures. Remarkably, already in 2 weeks old 4x*SNCA* neurons about 9% ± 2 of all FluoReSyn puncta were superimposable with TOMM20 positive mitochondrial structures in super-resolved imaging (**Figure 2F**). The index of proximity was further increased to 14% ± 4 in 5 weeks old 4x*SNCA* neurons (**Figure 2F**). A comparable juxtaposition was revealed using a co-staining between FluoReSyn and ATP5A1 (**Figure S10**), a subunit of the ATP synthase complex which has been previously co-localized with αSyn oligomers (23).

These results reveal that 4x*SNCA* neurons exhibit early aggregate formation in basal state without the need for any external inducers or stressors, and aggregation dynamics can be profiled and measured using a nanobody-based biosensor coupled with super-resolved imaging.

**Metabolic dysfunctions and calcium dysregulation in 4x*SNCA* DA neurons**

Given the significant physical interaction between αSyn aggregates and mitochondria, we thoroughly evaluated mitochondrial morphology and functionality using several readouts in 3 week old 4x*SNCA* and control DA neuronal cultures. Immunostaining for the protein of the external mitochondrial membrane TOMM20 showed a filamentous mitochondrial network in 2x*SNCA* DA neurons, which was significantly more fragmented in 4x*SNCA* cellular counterparts (**Figures 3A,B**). Collectively, these results indicated that 4x*SNCA* neurons have a global altered mitochondrial network. Next, we sought to determine whether these alterations might affect the overall bioenergetic profile of these cells. Thus, we evaluated the mitochondrial membrane potential (MMP) as determined by loading with TMRM, a cationic fluorescent dye that accumulates in negatively charged, polarized mitochondria and is released when MMP decreases. Under control conditions, 4x*SNCA* neurons showed a lower basal MMP than 2x*SNCA* or *SNCA*-KO neurons. In 2x*SNCA* DA and *SNCA*-KO neurons, addition of the ATP-synthase inhibitor oligomycin caused an expected hyperpolarization of MMP (**Figures 3C,D**). In contrast, 4x*SNCA* neurons showed a depolarization of MMP in response to oligomycin, suggesting that the mitochondrial respiratory chain was not sufficient to maintain a proton motive force, and that the ATP-synthase was working in reverse mode to maintain a physiological MMP (**Figures 3C,D**). An adverse effect of impaired respiration is the increase of radical oxygen species (ROS) generation. Thus, we monitored the intracellular oxidants using the fluorescent ROS-sensitive 2′-7′-dichlorodihydrofluorescein diacetate (DCFDA) on 4x*SNCA* and isogenic control neurons in basal conditions. Notably, ROS levels were strongly enhanced in the 4x*SNCA* compared to control DA neurons (**Figures 3E,G**). We, then, measured the reduced form of glutathione using the ThiolTracker Violet probe. In line with the heightened ROS levels, significantly lower levels of reduced glutathione were detected in 4x*SNCA* neurons compared to 2x*SNCA* or *SNCA*-KO cell counterparts (**Figures 3F,H**). Mitochondrial dysfunctions can lead to altered regulation of calcium response and, thus, we wondered whether calcium handling was affected by αSyn heightened levels in 4x*SNCA* DA neurons. We stimulated DA neuronal cultures after 3 weeks of differentiation with KCl (50 mM) to induce plasma membrane depolarization, neurotransmitter release and the opening of ligand- and voltage-gated calcium channels. We recorded calcium influx using the cell-permeable fluorescent calcium dye Fluo-8. Both 4x*SNCA* and isogenic control DA neurons showed a robust cytosolic calcium peak after KCl stimulation confirming the presence of functional calcium channels on the neuronal cell membrane (**Figure 3I**). However, neurons with *SNCA* multiplication exhibited a significant delay in cytosolic calcium recovery also over an extended period of time (4x*SNCA*: *3.4* ± 0.8; 2x*SNCA: 3.3* ± 0.9; *SNCA*-KO*: 1.5* ± 0.4 recovery rate) (**Figure 3I**). These results suggest that mitochondrial dysfunctions and calcium buffering alterations are early pathological signs in 4x*SNCA* DA neurons. Aberrant accumulation of αSyn assemblies have been shown to derange intracellular degradation processes in multiple ways (24,25). Accordingly, we detected enhanced mitophagy in 4x*SNCA* DA neurons as assessed by the simultaneous live staining of mitochondria and autophagolysosomes with the MitoTracker green and Lysotracker Red, respectively (**Figure S11A**). On the same line, levels of total LC3-GFP and lipidated LC3-II form were found markedly increased in 4x*SNCA* DA neurons, suggesting altered autophagy process dynamics (**Figure S11B,C**).

**4x*SNCA* DA neurons show accelerated αSyn accumulation and heightened cell death**

To determine whether the addition of exogenous αSyn fibrils assembled *in vitro* can seed the aggregation of endogenous αSyn in either cortical or DA neuronal cultures, we monitored the accumulation of pS129αSyn which occurred only during pathological αSyn aggregation. Synthetic αSyn fibrils91 were added into the culture medium of 3 weeks old neurons (0.5 ng/µl) and the extent of seeding was assessed by pS129αSyn immunohistochemistry 3 weeks later (**Figure 4A**). pS129αSyn was analyzed and compared between cortical (CNs) and DA neuronal (DANs) cultures differentiated from 4x*SNCA* or isogenic control iPSCs (**Figure 4B**). Interestingly, a significant higher fraction of both 4x*SNCA* cortical and DA neurons were positive for pS129αSyn staining when compared to their relative control cellular counterparts (**Figure 4B**) (4x*SNCA*-CNs: 24% ± 5; 4x*SNCA*-DANs: 35% ± 4; 2x*SNCA*-CNs: 3% ± 1; 2x*SNCA*-DANs: 8% ± 5). Importantly, *SNCA*-KO DA neuronal cultures exposed to synthetic αSyn fibrils91 were negative for pS129αSyn immunostaining confirming that the pS129αSyn signal strictly related to the aggregation of endogenous αSyn (data not shown). This indicates that the relative abundance of endogenous αSyn is a key determinant of the extent of its pathological aggregation. Moreover, a direct comparison between DA and cortical neurons with the same 4x*SNCA* genotype showed that the former population presented more numerous pS129αSyn positive cells with larger aggregates both in soma and neurites (4x*SNCA*-CNs: 20% ± 3; 4x*SNCA*-DANs: 58% ± 6) (**Figure 4B**). Calcium dysregulation and mitochondrial oxidative stress induce dopamine oxidation with the formation of oxidized toxic intermediates that stimulate ROS production, protein dysfunction through cysteinyl residue formation, glutathione inactivation and αSyn aggregate stabilization (26,27). We, then, employed near-infrared fluorescence to profile oxidized dopamine levels that were substantially increased in 3 week old 4x*SNCA* DA neuronal cultures when compared to control and *SNCA*-KO cellular counterparts (**Figure 4C**). Thus, heightened oxidized DA products can additionally contribute to the induction and following stabilization of αSyn aggregation on foot of the rapid accumulation of αSyn deposits in 4x*SNCA* DA neurons. Next, we evaluated the consequences of αSyn aggregation on the fitness of the neuronal cultures in 3 week *in vitro* cultures. Simultaneous two-color fluorescence discrimination between live and dead cells based on intracellular esterase activity and plasma membrane integrity showed that 4x*SNCA* DA neurons treated with αSyn fibrils displayed a marked loss of survival three weeks after initial treatment (**Figure 5**). In addition, some viability loss was also detectable although to a much lower magnitude in 4x*SNCA* cortical neurons and 2x*SNCA* DA neurons with or without pre-formed fibrils when compared to 2x*SNCA* cortical neurons (+fibrils: 4x*SNCA*-DANs 27% ± 3; 4x*SNCA*-CNs 18% ± 3; 2x*SNCA*-DANs 15% ± 3; 2x*SNCA*-CNs 14% ± 2. Basal conditions: 4x*SNCA*-DANs 15% ± 3; 4x*SNCA*-CNs 14% ± 2; 2x*SNCA*-DANs 14% ± 3; 2x*SNCA*-CNs 14% ± 2) (**Figure 5**). Taken together, the rapid endogenous αSyn aggregation induced through seeding by exogenous αSyn fibrils91 leads to a loss of cell viability mostly evident in DA 4x*SNCA* DA neurons preceded by large pS129αSyn inclusions and aberrant levels of oxidized DA.

**Generation of Lewy body-like structures in 4x*SNCA* DA neurons upon seeding**

3 weeks after initial seeding with exogenous αSyn fibrils91 4x*SNCA* DA neurons were the only population to develop numerous large round deposits within the somata in close vicinity with the nucleus and smaller and elongated inclusions along the neurites positive for pS129αSyn (**Figure 6A**). Next, we performed triple immunohistochemistry to characterize the organization and internal constituents of these structures. The majority of these inclusions were uniformly immunodecorated by antibodies recognizing neurofilaments and the microtubule associated protein TAU (**Figure 6B**). Moreover, most of these structures both in the soma and neurites were associated with organelles as shown by the co-staining of selective markers for mitochondria (GRIMM19) and lysosomes (LAMP1). GM130 positive cis-Golgi membranes were often found to partially enwrap pS129αSyn deposits in the soma (**Figure 6B**). Finally, the two resident pre-synaptic proteins Synaptophysin (SYP) and Synapsin-1 (SYN1) were found ectopically clustered within the pS129αSyn deposits, suggesting that a significant fraction of synaptic vesicles got entrapped into these structures (**Figure 6B**). We next performed correlative light-electron microscopy (CLEM) combining fluorescence with electron microscopic analysis on 5 weeks old iPSC-derived neurons treated with αSyn fibrils. Ultrastructural assessment revealed excessive crowding of vesicles and membranous structures both at the periphery and at the core of the αSyn-positive inclusions (**Figure S12A**). Moreover, altered numerous mitochondria with abnormal morphology were located at the border of the αSyn-positive structures (**Figure S12A**). Finally, immunoblots with the insoluble fraction of the cell lysates detected higher molecular species of αSyn only in 2 week old 4x*SNCA* DA neuronal cultures treated with exogenous fibrils (**Figure S12B**), suggesting that αSyn aggregation is advanced only in 4x*SNCA* neurons at this stage in vitro. Thus, we renamed αSyn inclusions in 4x*SNCA* neurons as Lewy body-like structures given their resemblance in both morphological traits and associated organelles with typical Lewy bodies detectable in most PD brain tissues (28). Collectively, these results show that the relative high abundance of native αSyn protein levels in 4x*SNCA* DA neurons predispose them, upon an exogenous seeding trigger, to the rapid development of Lew body-like structures.

**Disruption of TAX1BP1 strongly exacerbates αSyn pathology in DA neurons**

Protein aggregates are preferentially eliminated by selective autophagy (aggrephagy). TAX1BP1 is an autophagy receptor with a key role in promoting the clearance of Huntingtin and polyQ protein inclusions (29). However, whether TAX1BP1 has a similar role in αSyn protein quality control has remained yet unaddressed. Thus, we decided to inactivate *TAX1BP1* in the 4x*SNCA* iPSC line by CRISPR/Cas9 gene editing. We selected a sgRNA targeting exon 4 which is in common with all the 5 *TAX1BP1* gene isoforms and induced indel mutations by transient transfection of spCas9 and sgRNA expressing plasmids followed by antibiotic selection in proliferating iPSCs. We selected one iPSC line based on the following criteria: presence of indel mutations harboring frameshift in both *TAX1BP1* gene copies, protein loss by Western blotting, correct colony morphology, stable growth and homogeneous expression of the pluripotency genes (**Figures 7,S13**). Next, *SNCA* iPSCs with or without *TAX1BP1* gene deletion were differentiated in DA neuronal cultures for 5 weeks and then inspected for the accumulation of native αSyn aggregates by Thioflavin S staining and pS129αSyn immunohistochemistry. As reported earlier, among *SNCA* DA neuronal cultures only in the presence of 4x*SNCA*, scattered pS129αSyn aggregates were detectable at this time point. However, additional *TAX1BP1* gene inactivation triggered a 5- and 3-fold increase of Thioflavin S and pS129αSyn staining, respectively (**Figures 7B,C**). Exacerbated protein aggregation in 4x*SNCA;TAX1BP1*-KO DA neuronal cultures was confirmed by the highest levels of P62 protein accumulation among the cell lines in analysis (**Figures 7D,E**). Moreover, only after *TAX1BP1*-KO gene targeting a significant loss of viability was ascertained in DA neuronal cultures by using a two-color fluorescence live/dead assay (2x*SNCA:* 9% ± 3; 4x*SNCA* % ± 3; 4x*SNCA;TAX1BP1:* 36% ± 5)(**Figures 7F**). Thus, 4x*SNCA;TAX1BP1*-KO DA neuronal cultures accumulated marked αSyn aggregates levels sufficient to impair neuronal viability in basal conditions without the need of any exogenous trigger.

**Discussion**

Herein we generated and characterized a panel of isogenic iPSC lines derived from a PD patient with one among the smallest genomic triplication of *SNCA*. Using CRISPR/Cas9 gene editing each of the 4 *SNCA* genes was destroyed with indel mutations inducing frameshift or exon3/4 deletions, generating a set of isogenic iPSCs with an incremental number of *SNCA* gene alleles from 4 (parental line) to none (*SNCA*-KO). PD patients with *SNCA* triplication are extremely rare and for this reason the multiple studies in the literature were performed using iPSCs derived from only two individuals, a 48 years old male (17,30) and a 55 years old female (16,18). Most of the early iPSC lines were generated with integrating reprogramming vectors and only in one case an isogenic control iPSC line is available although its corresponding parental line was reprogrammed with retroviral vectors (15). Thus, the new set of isogenic iPSCs generated in this study significantly expands the number of iPSC lines available for this important PD-causing genetic alteration and provides for the first time a full panel of isogenic cells that differ for only the incremental number of *SNCA* genes. Generating iPSCs from more individuals with *SNCA* triplication is also extremely relevant for identifying ultimately the molecular determinants explaining symptomatic differences between patients. In fact, despite the general severity of the disease phenotype in these patients, the two siblings on this study presented substantial differences in clinical motor and cognitive manifestations with different ages of onset (19). Thus, a wide collection of iPSCs from these patients together with their deep, homogeneous and longitudinal clinical assessments would represent a powerful resource to investigate *SNCA* genetic modifiers.

We showed that αSyn protein levels increase in accordance with the number of functional *SNCA* genes reaching a maximal peak in 4x*SNCA* cells. We showed that endogenous αSyn abundance was associated with an early αSyn aggregation in 2 weeks old DA neuronal cultures. These conclusions were reached by exploiting the new nanobody-based biosensor FluoReSyn which provided a sensitive and effective tool to visualize small αSyn assemblies when coupled with super-resolution imaging. Its high specificity was confirmed by the lack of any meaningful staining in isogenic *SNCA*-KO cells. These results emphasize the importance to have also available a *SNCA* knock-out cell line in the panel of iPSCs for specificity readouts. This analysis provided a neat description of the development of endogenous αSyn aggregates in early differentiated DA neurons in basal conditions without any exogenous stimuli. This finding identified 4x*SNCA* DA neurons as an excellent cellular system to investigate biophysical determinants of αSyn aggregation and identify specific inhibitors of this process by candidate testing or screening campaigns. These data are in line with previous observations, in which αSyn aggregates in 4x*SNCA* DA neurons were inferred by the proximity ligation assay (PLA) (13). However, this is a cumbersome technique with extremely low through-put. In contrast, AAV-based FluoReSyn expression enables a direct, multi-color and rapid assessment of αSyn assemblies on *in vitro* neuronal cultures, which is compatible with longitudinal analysis and time-lapse imaging. As a valuable example for this, FluoReSyn staining enabled us to identify a significant proportion of αSyn aggregates associated with mitochondria in both early and late neuronal cultures. This finding prompted us to investigate mitochondrial alterations in 4x*SNCA* DA neurons revealing the occurrence of mitochondrial fragmentation, loss of membrane potential and heightened oxidative damage. Interestingly, in concomitance with mitochondrial dysfunctions we observed altered calcium signaling in 4x*SNCA* DA neurons with a significant delay in cytosolic calcium efflux after KCl stimulation. High cytosolic calcium levels may also favor the opening of the mitochondrial permeability transition pore (PTB) which induces loss of the mitochondrial membrane potential (31, 32). Thus, calcium mishandling can represent an early alteration in 4x*SNCA* DA neurons which destabilizes mitochondrial homeostasis.

In addition, we showed that upon challenge to exogenous αSyn fibrils to accelerate endogenous αSyn seeding, 4x*SNCA* DA neurons showed the highest burden of αSyn aggregates which significantly impaired their survival in culture. In fact, 4x*SNCA* DA neurons showed the largest cell fraction with accelerated development of αSyn deposits as compared to 4x*SNCA* cortical neurons and isogenic control 2x*SNCA* neurons. This selective impairment should be routed within the specific DA metabolism. In fact, we found aberrant levels of oxidized DA in seeded 4x*SNCA* DA neurons which can further enhance oxidative damage and, thereby, accelerate αSyn aggregation. Given that altered oxidized DA has been already described in iPSC-derived neurons of other idiopathic and familiar PD patients (14), its accumulation can be considered a key event in the pathophysiological mechanisms leading to DA neuronal degeneration by enhancing oxidative damage and facilitating αSyn aggregation and, thereby, establishing a detrimental vicious cycle between these two pathological events.

We showed that over time the αSyn aggregates in seeded 4x*SNCA* DA neurons developed over time into large deposits localized in soma and processes. Immunohistochemistry analysis indicated that they are enriched in mitochondrial and lysosomal components suggesting a stable association with these organelles or part of them. Intriguingly, a recent ultrastructural analysis in *post-mortem* patient brain tissues nicely showed that major constituents of Lewy bodies are dysmorphic organelles and vesicles intermingled with heterogeneous membrane fragments (28). Given the high resemblance of their morphological structures, we consider appropriate to refer to αSyn aggregates in seeded 4x*SNCA* DA neurons as Lewy body-like structures. Similar findings were reported by Mahul-Mellier et al. (33) applying advanced imaging approaches to study the dynamics of formation of seeded αSyn aggregation in mouse hippocampal neurons. These results indicate that αSyn aggregation when forced through exogenous seeding can follow similar principles of spatial organization and internal constituents between different neuronal cell types. However, our results also show that only 4x*SNCA* DA neuron viability was severely affected by αSyn aggregation, highlighting that cell-type specific pathophysiological consequences caused by the development of these aggregates require the generation of authentic *in vitro* counterparts of midbrain-specific DA neurons.

Collectively, our data suggest that 4x*SNCA* DA neurons offer a remarkable cellular platform to investigate mechanisms and mediators of native and seeded αSyn aggregation. As proof-of-principle for this, we assessed the role of TAX1BP1, a key autophagy receptor, in this scenario.

Simultaneous inactivation of *TAX1BP1* by CRISPR/Cas9 gene editing in 4x*SNCA* DA neurons showed that αSyn aggregation is more aggressive and diffuse, revealing a key role of TAX1BP1 in modulating the clearance of αSyn aggregates. In addition, *TAX1BP1* gene loss sensitizes 4x*SNCA* DA neurons to cell death even in the absence of exogenous stimulated seeding. This result indicates that TAX1BP1 deficiency enhances total endogenous αSyn protein content which jeopardizes the fitness and survival of 4x*SNCA* DA neurons.

In conclusion, we produced iPSCs starting from a PD patient harboring a new and compact *SNCA* triplication and using CRISPR/Cas9 gene editing we generated an isogenic allelic series of iPSCs by inactivating each of the *SNCA* alleles. By differentiating midbrain DA neuronal cultures, we identified the early accumulation of endogenous αSyn aggregates by combining a new and sensitive biosensor with super-resolved imaging. In addition, we revealed novel cellular dysfunctions in both native and seeded conditions in 4x*SNCA* DA neurons which can partly explain the selective vulnerability of these neurons to αSyn aggregation burden.

**Methods**

*Plasmid cloning*

sgRNAs were designed using the online software CRISPOR (34) and selected according to the higher specificity score. Then, sgRNAs were cloned into the LV-U6-filler-crRNA-Ef1α-Blasticidin vector, previously described in (35). The expression vector pCAG-spCas9-P2A-Puromicin (35) was employed for transient expression of spCas9 in human iPSC cultures.

*Cell cultures*

Peripheral blood samples from patients were obtained from the IRCCS Neuromed of Pozzilli through informed consent approved by the local Ethical committee (Clinical Trials: #NCT03682458; ID: CGM-02). Primary peripheral blood mononuclear cells (PBMCs) were isolated from the blood withdrawals and reprogrammed by non-integrating Sendai viruses expressing the four Yamanaka’s factors using the CytoTune-2.0 kit (ThermoFisher) at the IRCCS San Raffaele Hospital in Milan. iPSC lines were maintained in feeder-free conditions in mTeSR1 (Stem Cell Technologies) and expanded in HESC qualified Matrigel (Corning)-coated 6-well plates.

*Gene editing*

iPSCs were maintained in feeder-free conditions in mTeSR1 (Stem Cell Technologies) and seeded in HESC qualified Matrigel (Corning)-coated 6-well plates. At >80% confluency the iPSCs were transfected with two separate plasmids expressing sgRNA-blast and spCas9-Puro using Lipofectamine^TM^ Stem Reagent (Thermo). Co-transfected colonies were then selected by the combination of puromycin (1 µg/ml, Sigma) and blastidicin (10 μg/ml, ThermoFisher Scientific) and then isolated by single colony picking. Finally, cell clones with the correct genomic deletions were assessed by genomic PCR analysis followed by Sanger sequencing.

*Neuronal differentiation*

NPCs were generated as previously described with appropriated optimization (36). Briefly, iPSCs were dissociated in cell clusters using Accutase (Sigma-Aldrich) and seeded onto low-adhesion plates in mTeSR1 supplemented with N2 (1:200, ThermoFisher Scientific), Pen/Strept (1%, Sigma-Aldrich), human Noggin (0.5 μg/ml, R&D System), SB431542 (5 μM, Sigma-Aldrich) and Y27632 (10 μM, Selleckchem). After 10 days, embryoid bodies were seeded onto matrigel-coated plates (1:100, matrigel growth factor reduced, Corning) in DMEM/F12 (Sigma-Aldrich) supplemented with N2 (1:100), non-essential amino acids (1%, MEM NEAA, ThermoFisher Scientific) and Pen/Strept. After 10 days, rosettes were dissociated with Accutase and plated onto matrigel coated-flasks in NPC media containing DMEM/F12, N2 (1:200), B27 (1:100, ThermoFisher Scientific), Pen/Strept (1%) and FGF2 (20 ng/ml, ThermoFisher Scientific). For differentiation, NPCs were dissociated with Accutase and plated on matrigel-coated 6-well plates (1× 300000 cells per well) in NPC medium. Two days after, the differentiation medium containing Neurobasal (ThermoFisher Scientific), Pen/Strep (1%), B27 (1:50), with SU5402 (Sigma-Aldrich, 10 µM), PD0325901 (Sigma-Aldrich, 8 µM), DAPT (Sigma-Aldrich, 10 µM) was added and kept for 2 days. Differentiation medium was replaced every day with a fresh one on days 1 and 2. At day 3, cells were detached by Accutase solution incubation at 37 °C for 10 min in order to obtain a single-cell suspension. Cells were centrifuged, counted, and seeded at a density of 55,000 cells/cm^2^ onto poly-L-lysine/laminin/fibronectin (all from Sigma-Aldrich, 100 µg/ml, 2 µg/ml, 2 µg/ml)-coated plates in neuronal maturation medium supplemented with ROCK inhibitor Y27632 (10 µM) for the first 24 h. Neuronal maturation medium was composed by Neurobasal A (ThermoFisher Scientific) supplemented with 1× B-27 supplement, 2 mM glutamine, 1% Pen/Strept, BDNF (Peprotech, 20 ng/ml), ascorbic acid (Sigma-Aldrich, 100 nM), Laminin (1 μg/μl), DAPT (10 μM), dbcAMP (Selleckchem, 250 μM). The culture medium was replaced the next day to remove the ROCK inhibitor, and then half of the medium was replaced with a fresh neuronal maturation medium twice a week.

Dopaminergic neurons were generated as previously described with small modifications (37,38). iPSCs were dissociated with Accutase and plated on matrigel-coated 6-well plates (1× 200.000 cells per well) in mTeSR1 medium. One day after, the medium was replaced by differentiation medium containing LDN193189 (100 nM, Stemgent), SB431542 (10 mM, Tocris), SHH C25II (100 ng ml21, R&D), Purmorphamine (2 mM, Sigma-Aldrich), FGF8 (100 ng/ml, Sigma-Aldrich) and CHIR99021 (CHIR; 3 mM, Milteny) in mTeSR1 medium for 11 days. mTeSR1 medium was gradually shifted to N2 medium starting on day 5 of differentiation. Half medium was changed every 2-3 days. After 9 days, cells were dissociated with Accutase and plated on poly-L-lysine/laminin-coated 24-well plates for the final maturation. BDNF (10 ng/ml), GDNF (10 ng/ml), DAPT (10 μM, Sigma-Aldrich) and Ascorbic Acid (10 μM, Sigma-Aldrich) were added from day 20 to promote neuronal maturation and survival.

*MPLA analysis*

The commercially available kit P051-P052 (MRC- Holland, Amsterdam, Netherlands) was used for the multiplex dosage of exons in iPSC genomic DNA for the following genes: TNFRSF9 (1 probe in P051), DJ1 (4 probes in P051), ATP13A2 (2 probes in P051, 2 probes in P052), SNCA (5 probes in P051, 1 probe in P052), LPA (1 probe in P051), PARKIN (12 probes in P051, 12 in P052), LRRK2 (8 probes in P052), PINK1 (8 probes in P051), GCH1 (5 probes in P052), PACRG (1 probe in P052), CAV1/2 (2 probes in P052), and UCHIL1 (4 probes in P052).

*α-Syn fibrils91 preparation*

Human wild-type *SNCA* was expressed in *E. coli* BL21 DE3 CodonPlus cells (Agilent Technologies) and purified as described previously (39). Pure monomeric αSyn was assembled into the fibrillar polymorph, named fibrils91 as it exhibits the highest seeding potency in neurons *in vitro* and *in vivo* (40,41) in 20mM KPO4, 150mM KCl at 37°C under continuous shaking in an Eppendorf Thermomixer set at 600 r.p.m for 7 days (42). The aggregation reaction was followed by withdrawing aliquots (10 µl) from the reaction at different time intervals, mixing them with Thioflavin T (400µl, 10 µM final) and recording the fluorescence increase on a Cary Eclipse Fluorescence Spectrophotometer (Varian Medical Systems Inc.) using an excitation wavelength = 440 nm, an emission wavelength = 480 nm and excitation and emission slits set at 5 and 10 nm, respectively. The resulting αSyn fibrils91 were centrifuged twice at 15,000*g* for 10 min and re-suspended twice in PBS at 250µM. αSyn fibrils91 were fragmented by sonication for 20 min in 2 mL Eppendorf tubes in a Vial Tweeter powered by an ultrasonic processor UIS250v (250 W, 2.4 kHz; Hielscher Ultrasonic, Teltow, Germany) to generate fibrillar particles with an average size of 42-52 nm as assessed by TEM analysis. The fibrillar nature of aSyn was assessed by Transmission Electron Microscopy (TEM) after adsorption of the fibrils onto carbon-coated 200 mesh grids and negative staining with 1% uranyl acetate using a Jeol 1400 transmission electron microscope before and after fragmentation. The images were recorded with a Gatan Orius CCD camera (Gatan, Pleasanton, CA, USA). We further quantified the endotoxin levels in αSyn fibrils91 preparations as described previously (43, 44) to make sure that endotoxin levels were below 0.02 endotoxin units/mg (EU/mg) using the Pierce LAL Chromogenic Endotoxin Quantification Kit.

*Immunocytochemistry*

Cells were seeded on matrigel-coated glass coverslips and they were fixed for 20 min in ice in 4% paraformaldehyde (PFA, Sigma), solution in phosphate-buffered saline (PBS, Euroclone). Then, cells were permeabilized for 30 min in blocking solution, containing 0.5% Triton X-100 (Sigma-Aldrich) and 10% donkey serum (Sigma-Aldrich), and incubated overnight at 4 °C with the primary antibodies in blocking solution. Then, cells were washed with PBS and incubated for 1 h at room temperature with Hoechst and with secondary antibodies. The following antibodies were used: anti-OCT4 (1:100, Abcam), anti-NANOG (1:100, Abcam), anti-FOXA2 (1:300, Abcam), anti-NESTIN (1:300 Millipore), anti-TH (1:200, Immunological Sciences), anti-MAP2 (1:500, Immunological Sciences), anti-TOMM20 (1:300, Novus), anti-α-Synuclein (clone LB509, 1:100, Thermo), anti-GFP (1:500, Thermo), anti-α-Synuclein (phosphoS129, 1:300, Abcam), anti-TAU (1:500, Millipore), anti-LAMP1 (1:500, Abcam), anti-Synapsin1 (1:500, Synaptic Systems), anti-SMI311 (1:500, BioLegend), anti GM130 (1:300, BD), anti-GRIM19 (1:300, Abcam). All the secondary antibodies used for the immunofluorescence staining are Alexa Fluor^TM^.

*Molecular cloning and viral production*

For the LV:LC3-GFP, LC3 coding region followed by GFP coding sequence was cloned into a lentiviral vector downstream to the EF-1α promoter. For the LV:Syn-GFP, GFP coding sequence was cloned under the control of α-Synapsin promoter in a lentiviral vector. Replication-incompetent VSVg-coated lentiviral particles were packaged in 293T cells.

*Cell lysate preparation and immunoblotting*

Cell lysates were prepared with a buffer containing 50 mM Tris-Hcl (pH 7.4), 150 mM NaCl, 0,1% SDS and 1% Triton-X100. Protease and phosphatase inhibitor cocktails (Roche) were added immediately prior to use. The protein concentration was measured by BCA Protein Assay Kit (Thermo Fisher Scientific). Generally, 25 μg of protein lysates were loaded onto SDS-polyacrylamide gel electrophoresis and protein transferred onto a nitrocellulose membrane. The membrane was blocked with 5% no-fat dry milk in PBST (phosphate buffered saline with 0.1% Tween-20). For αSYN fractioning immunoblot, cells lysates were processed in order to collect the PBS- and SDS-soluble fractions as described by Luk et al. (45). Briefly, cells were washed with PBS and scraped into ice-cold lysis buffer (50 mM Tris/150 mM NaCl/1% Triton X-100, pH 7.4) containing protease and phosphatase inhibitors.  After lyses, the supernatant was clarified by 100,000  g centrifugation at 4°C for 1 hr. The resulting supernatant represents the PBS-soluble fraction. Then pellets were solubilized in PBS-SDS (1% SDS w/v) by sonication and centrifuged at 100,000  g for 30 min at 25°C. Samples were loaded in a gradient gel (4%–12% Bis-tris gel, NP0322BOX; Invitrogen). The transfer was performed for 1 hr at 100 V on nitrocellulose membrane (GE Healthcare). Membranes were then blocked in 5% non-fat-milk for 1 hr and the primary antibody (Syn211) was incubated over-night at 4°C. After incubation with the appropriate HRP-conjugated secondary antibody for 30 min, the signal was then revealed and processed as previously described.

The primary antibodies and dilutions used were the following: anti-ACTIN (1:1000, Sigma-Aldrich), anti-CALNEXIN (1:2000, Sigma-Aldrich), anti-P62 (1:4000, Novus), anti-α-Synuclein (clone Syn211, 1:1000, Millipore), anti-TAX1BP1 (1:1000, Bethyl Laboratories). Antibody incubation was followed by a horseradish peroxidase (HRP)-conjugated goat anti-mouse and anti-rabbit secondary antibodies (1:10000, Dako). The signal was revealed using the ECL-chemiluminescence kit (GE Healthcare Amersham ECL Western Blotting Detection Reagent) and detected with ChemiDoc Touch Imaging System (Biorad). Quantitation of band intensity on unsaturated exposures was performed with the Volume tool of the Image Lab 5.0 software. The adjusted values of the proteins of interest were normalized on those of Actin or Calnexin bands of the corresponding lanes.

*Structured illumination microscopy (SIM)*

Samples for SIM were cultured on laminin-coated glass coverslips. They were fixed and stained for intracellular markers as described in the Immunostaing section. SIM was performed on an Elyra (Zeiss, ELYRA 7 with Lattice SIM²), using a 60x oil objective. Images were processed and channels aligned using the automatic settings on the ZEN Black software (Zeiss).

*Mitochondrial Morphology*

Dopaminergic neurons from patients and isogenic controls were seeded on matrigel-coated glass coverslips. Mitochondrial morphology was assessed by TOMM20 immunostaining. Cellular fluorescence images were acquired with a Nikon Eclipse Ni microscope. Images were collected using a X63/1.4 oil objective and analyzed using Mito-Morphology macro in ImageJ.

*Mitochondrial Membrane Potential determination*

Neurons from patients and isogenic controls were seeded on matrigel-coated glass coverslips. Mitochondrial membrane potential was measured based on the accumulation of Tetramethylrhodamine Methyl Ester (TMRM; Life Technologies). Cells were incubated with 2μM Oligomycin (Sigma-Aldrich) and loaded with 250nM TMRM for 20 minutes. At the end of each experiment, mitochondria were fully depolarized by the addition of 1μM of the protonophore carbonyl cyanide 4-(trifluoromethoxy) phenylhydrazone (FCCP; Sigma-Aldrich). Cellular fluorescence images were acquired every 3 minutes for each sample with a fluorescence microscope (Nikon Eclipse Ni) and analyzed by ImageJ.

*Mitophagy analysis*

For assessment of the mitophagy neuronal cultures were incubated with MitoTracker Green FM (1 μM final concentration) (Thermo Fisher Scientific) for 30 minutes at 37°C and then extensively washed with PBS. LysoTracker Red DND-99 (1 μM final concentration) (Thermo Fisher Scientific) was then added, and cells were immediately observed on a Nikon LiveScan Confocal Microscope. The green and red signal colocalization rate was evaluated using the colocalization counter JACOP available in Fiji software. For each condition, the colocalization of these two signals was also determined by manual counting of fluorescent puncta.

*ROS and glutathione quantitative analysis*

iPSC-derived neurons were incubated with Alexa Fluor 647 mouse anti-human CD56 (anti-NCAM, BD Biosciences, diluted 1:40) for 1 h, with 20 μM of 20 ,70 -dichlorodihydrofluorescein diacetate (H2DCFDA; Molecular Probes) for 15 min, and with 2 μg/ml of Hoechst 33342 for 2 min at 37°C. Cells were washed multiple times and randomly analyzed using a fluorescence microscope (Nikon Eclipse Ni). The DCFDA signal from NCAM–positive cells was quantified by ImageJ software analysis to determine the relative ROS content.

For Glutathione analysis, neurons were incubated with 20 μM ThiolTracker Violet (Invitrogen) for 30 min at 37°C, and then, washed with PBS and analyzed by fluorescence microscope (Nikon Eclipse Ni). The ThiolTracker Violet fluorescence was collected to compare relative glutathione contents by ImageJ image analysis.

*Live/Dead labeling*

Cells plated on matrigel-coated glass coverslips were incubated with 5 μM of Live/Dead Assay Kit (ThermoFischer) for 10 min at 37 °C. Then, cells were washed with PBS and acquired by inverted fluorescence microscope (Nikon, Eclipse Ti). For the analysis, the images were collected using a X20/0.45 objective and analyzed using ImageJ.

*Measurement of [Ca^2+^]*

Cells were seeded in 96 well optic black microplates and loaded with 4μM of Fluo-8 in KRH for 30 min at 37°C followed by 2x KRH washes. The images were taken with a fully automated microscope, ArrayScan XTI HCA Reader (Thermo Fisher Scientific), equipped with a liquid handling module for the dispensing of the stimuli and a Photometrics X1, 14bit, high resolution camera. A Zeiss LD Plan-NEOFLUAR 20x/0,4NA objective was used to capture one image per well with the following settings: Fluo-8 (excitation wavelength: 485/20nm, emission filter 520/21nm) and Hoechst (excitation wavelength: 386/23nm, emission filter: 440/20nm) were detected both with camera gain2 and 60% of LED intensity. 30 frames were acquired at 0,5 Hz with 20msec exposure time for Fluo-8 and 10msec exposure time for Hoechst. 50ul of 50 mM KCl was dispensed at a rate of 50ul/sec. At least 4 baseline images were acquired. The analysis was done with HCS Studio software using SpotDetector bioapplication (Thermo Fisher Scientific). Hoechst positive nuclei were identified and counted in all the images. The nuclei segmentation method was identical for all the conditions within the same experiment but was optimized among the experiments. The 3D_Surface Fitting method (parameter value:255) was applied for the removal of the background before the quantification of the Fluo-8 signal. The mean intensity of the Fluo-8 signal was then quantified in the cell body area of each single cell and the normalized mean intensity was obtained for each well.

*Near-infrared fluorescence for oxidized dopamine assessment*

Oxidized dopamine analysis were performed as described by Burbulla et al. (14). Briefly, for each experiment 1 x 10e6 neurons were harvested and cell pellets were homogenized in 1% Triton X-100 lysis buffer. Insoluble pellets were extracted in 2% SDS/50mM Tris by boiling and sonication. Leftover insoluble pellets from a 150,000x g spin (30min, 4°C) were further extracted in 1N NaOH followed by incubation at 55°C. Then, the solutions were lyophilized in a Speed Vac Concentrator until the pellet was completely dry. Pellets were washed once with ultrapure H2O for removal of hydroxides, and then lyophilized again before the dried pellet was taken up in ultrapure H2O and finally analyzed. 10mM oxidized dopamine (DA) stock was used as a standard and prepared starting from 10mM DA (in D-PBS) mixed with 20mM NaIO4. Each experimental sample or standard dilution was dropped onto Nylon membranes and scanned using an Odyssey infrared imaging system. Samples were quantified by obtaining integrated spot intensities using Odyssey infrared imaging software (LI-COR).

*Measurement of α-Syn protein levels in cell culture supernatant*

For the quantification of the α-Syn protein neurons were plated on laminin-coated 6-well plates. Collection of the medium was carried out after 7 days in which the medium was not changed and the analysis of protein in cell culture was performed with Human Alpha-Synuclein ELISA Kit (Abcam).

*ThT Fluorescence staining*

iPSC-derived neurons were incubated with Alexa Fluor 647 mouse anti-human CD56 (anti-NCAM, BD Biosciences, diluted 1:40) for 1 h, with 1 mM of Thioflavin T (Sigma-Aldrich) for 15 min, and with 2 μg/ml of Hoechst 33342 for 2 min at 37°C. Cells were washed multiple times and randomly analyzed using a fluorescence microscope (Nikon Eclipse Ni).

*Correlative light-electron microscopy (CLEM)*

Cells were grown on finder grids and Z-stacks of cells of interest were taken with the PerkinElmer UltraView ERS confocal microscope. The coordinates of the cells on the finder grid were determined by bright-field microscopy. Cells were fixed in 1% glutaraldehyde in 0.1 M cacodylate buffer (Sigma) and post-fixed with 1.5% potassium ferricyanide, 1% OsO4 in 0.1 M cacodylate buffer. Cells were stained with 0.5% uranyl acetate overnight, dehydrated in ethanol, and embedded in epon. After baking for 48 h at 60°C, the resin was released from the glass coverslip by temperature shock in liquid nitrogen. Serial sections (70–90 nm) were collected on carbon-coated formvar slot grids and imaged with a Zeiss LEO 512 electron microscope. Images were acquired by a 2k × 2k bottom-mounted slow-scan Proscan camera controlled by EsivisionPro 3.2 software. Immunofluorescence and IEM images were aligned using Icy bioimage analysis.

*Statistics*

All values are expressed as mean ± SEM. Differences between means were analysed using the Student t-test, one-way or two-way analysis of variance (ANOVA) depending on the number of groups and variables in each experiment. *In vitro* and *in vivo* data were then submitted to Tukey or Bonferroni post-hoc test using GraphPad Prism software. The null hypothesis was rejected when P-value was < 0.05.

**Acknowledgements**

We thank D. Zambroni for expert support on calcium imaging. We are thankful to V. Tiranti, E. Cattaneo, A. Ulusoy and D. Di Monte for sharing reagents and antibodies. This work was supported by the Italian Ministry of Health (PE-2016-02363550) and Mitochondrial Dysfunction in Parkinson’s Consortium (PD-MitoQUANT). PD-MitoQUANT has received funding from the Innovative Medicines Initiative 2 Joint Undertaking under grant agreement No. 821522. This Joint Undertaking receives support from the European Union’s Horizon 2020 research and innovation programme and EFPIA

**Conflict of interest**

The authors declare no competing interests.

**Author contributions**

V.B. conceived this study. V.B. and A.I. designed the experiments. A.I. performed and analyzed all the experiments with the help of L.M and S.G.G. R.F and S.G. performed the genome analysis of the iPSC lines. F.O., O.C. and J.P. provided key materials and help in interpretating the results. G.O. performed mitophagy and autophagy analyses. A.R. carried out CLEM imaging. R.M. supervised the production and characterization of recombinant aSyn fibrils. V.B. wrote the manuscript and ensured funding.

**Data Availability statement**

The datasets used and/or analyzed during the current study are available from the corresponding author on reasonable request.

**Figures**

**Figure 1: Generation of a set of iPSCs with an incremental number of *SNCA* alleles by CRISPR/Cas9 gene editing.** (**A**) Schematic representation of the protocol for generating 4x, 3x, 2x and 1x*SNCA* iPSC lines. (**B**) Immunoblot analysis and quantification for αSYN protein in 4x, 3x, 2x and 1x*SNCA* NPCs (n = 3 independent experiments). (**C**) Quantification of αSYN protein levels in cell supernatants (n = 3 independent experiments). (**D**) iPSC colonies immunostained with the pluripotency markers OCT4, NANOG and detected in bright field for morphology appearance assessment. Representative images and quantification for midbrain DA neuronal cultures from 4X, 3X, 2X and 1X *SNCA* and SNCA-KO iPSCs immunostained with the DA marker TH and neuronal marker MAP2 (3 fields, 3 independent experiments). Values are mean ± SEM of n = 3 independent experiments. *p < 0.05; **p < 0.01, ***p < 0.001. Statistical analysis is performed using one-way ANOVA followed by Tukey post-test. Scale bars, 100 µm

**Figure 2: FluoReSyn imaging enables the early detection of endogenous αSyn aggregates in DA neurons.** (**A**) Schematic view of the protocol to infect DA neuronal cultures with AAV-FluoReSyn. (**B**) Immunostaining for FluoReSyn shows high level αSyn assemblies in 4x*SNCA* compared to isogenic control 2x*SNCA* DA neurons. (**C-D**) Quantification of number of αSyn aggregates (**C**) and their size (**D**) in 2x and 4x*SNCA* DA neurons. Dots indicate quantifications in 5 different fields for 4 independent experiments. (**E**) Representative images of Thioflavin S signal in 4x*SNCA* DA neurons. (**F**) Images of super-resolved imaging show overlapping between αSyn puncta and mitochondrial structures in 2 and 4 weeks old 4x*SNCA* DA neurons. Values are mean ± SEM. *p < 0.05, **p < 0.01, ***p < 0.001. Statistical analysis is performed using one-way ANOVA followed by Tukey post-test. Scale bars, 100 µm.

**Figure 3:** **4xSNCA mDA neurons exhibit mitochondrial dysfunctions, heightened oxidative stress and calcium mishandling.** (**A**) Representative images of mitochondrial morphology stained with TOMM20 (green) in 2x and 4x*SNCA* mDA. (**B**) Quantification of mitochondrial morphology confirming the fragmented morphology in 4x*SNCA* mDA compared to isogenic control. Dots indicate quantifications in 5 different fields for 4 independent experiments. (**C-D**) Analysis of the mitochondrial membrane potential by TMRM live staining. TMRM signal is followed over time during exposure to Oligomycin (O) and FCCP (F) in SNCA-KO, 2x and 4x*SNCA* DA neuronal cultures. Quantification of TMRM signal is normalized on the number of cells (n = 25 somata). (**E-F**) Representative images of neuronal cultures stained with the ROS-sensitive fluorescent probe DCFDA (**E**) and ThiolTracker Violet (**F**). The antibody for the Neural Cell Adhesion Molecule (NCAM) is employed for live staining analysis of neurons. (**G-H**) Quantification of DCFDA (7 fields; 3 biological independent experiments) and ThiolTracker Violet fluorescence (6 fields; 3 biological independent experiments) reveals heightened oxidative state in 4x*SNCA* DA neurons. (**I**) Representative Ca^2+^ images in response to KCl and single-cell trace showing delayed recovery after KCl stimulation in 4x*SNCA* mDA neurons. Quantification of the Fluo-8 recovery rate is calculated from the Fluo8 intensity values recorded 80 seconds after peak stimulation with KCl. Values are mean ± SEM. ***p < 0.001. Statistical analysis is performed using one-way ANOVA followed by Tukey post-test. Scale bars, 100 µm.

**Figure 4: Accelerated accumulation of αSYN aggregates in 4xSNCA mDA compared to cortical neurons.** (**A**) Schematic view of the protocol to intoxicate cortical and DA neuronal cultures with P91 αSyn fibrils. (**B**) Immunostaining for pS129αSYN in *SNCA*-KO 2x and 4x*SNCA* iPSC-derived cortical and DA neuronal cultures. Representative high-magnification images of pS129αSyn+ aggregates in 4x*SNCA* iPSC-derived cortical and DA neuronal cultures after exposure to P91 fibrils. Quantification of the number of neurons exhibiting pS129αSyn+ aggregates (3 fields; 3 biological independent experiments) and the ratio between the areas of aggregates and somata (n = 25 somata). αSYN aggregate size was calculated by measuring the perpendicular axis respect to the larger diameter for all assemblies (50 aggregates in total). (**C**) Near-infrared fluorescence to profile oxidized dopamine and quantification shows high levels in 4x*SNCA* DA neuronal cultures compared to control and SNCA-KO DA neuronal cultures. Values are mean ± SEM. *p < 0.05, ***p < 0.001. Statistical analysis is performed using Student *t*- test, one-way ANOVA followed by Tukey post-test and two-way ANOVA followed by Bonferroni post-test. Scale bars, 100 µm.

**Figure 5: Impaired survival of 4xSNCA mDA neurons exposed to fibrils.** (**A**) Schematic view of the protocol to intoxicate cortical and DA neuronal cultures with P91 αSyn fibrils. (**B**) Live/dead fluorescence staining in iPSC-derived cortical neurons with or without exposure to P91 fibrils. (**C**) Live/dead fluorescence staining in iPSC-derived DA neurons with or without exposure to P91 fibrils. (**C-D**) Quantification of cell viability and toxicity of 2x and 4x*SNCA* cortical and DA neurons with or without P91 fibril exposure (for each group n=50 fields, automatic counts). Values are mean ± SEM of n = 3 biological independent experiments. **p < 0.01, ***p < 0.001. Statistical analysis is performed using two-way ANOVA followed by Bonferroni post-test. Scale bars, 100 µm

**Figure 6: Generation of Lewy body-like structures in seeded 4xSNCA mDA neurons.** (**A**) Schematic view of the protocol to intoxicate DA neuronal cultures with P91 αSyn fibrils. (**B**) Representative images of Lewy body-like structures stained with different markers for neurofilament (TAU), mitochondria (GRIMM19), lysosomes (LAMP1), cis-Golgi membranes (GM130) and synaptic protein (SYN1, SYP). Scale bar, 100 µm.

**Figure 7: TAXBP1 inactivation exacerbates αSyn aggregates in DA neurons.** (**A**) Immunoblot analysis for TAX1BP1 protein confirm inactivation of gene in 4x*SNCA*;TAXBP1-KO iPSCs. * indicates a non-specific signal. (**B**) Representative images **of** 4x*SNCA*;TAXBP1-KO stained with Thioflavin S and pS129αSyn. (**C**) Quantification of number of pS129αSyn+ aggregates in SNCA-KO, 2x , 4x*SNCA,* and 4x*SNCA*;TAXBP1-KO DA neurons. Dots represent quantifications in 5 fields for 4 independent experiments. (**D-E**) Immunoblot and quantification of p62 signal in SNCA-KO, 2x, 4x*SNCA,* and 4x*SNCA*;TAXBP1-KO DA neurons (n = 3 independent experiments). (**F**) Live/dead fluorescence staining and quantification of cell viability and toxicity 2x, 4x*SNCA,* and 4x*SNCA*;TAXBP1-KO DA neurons. Values are mean ± SEM of n=3 independent experiments (n = 50 fields for each group, automatic counts). **p < 0.01, ***p < 0.001. Statistical analysis is performed using one-way ANOVA followed by Tukey post-test. Scale bars, 100 µm.

**Supplementary Figures**

**Supplementary Figure 1:** (**A**) qPCR analysis of pluripotency genes in iPSCs compared to the corresponding human fibroblasts. (**B**) Karyotype analysis of iPSC lines displayed a normal euploid chromosome content. (**C**) MLPA analysis confirms the presence of the SNCA triplication. (**D**) Representative images and quantification of 4x*SNCA* NPCs show high level of αSyn compared to *SNCA*-KO and isogenic control neurons. Values are mean ± SEM. Dots indicate quantifications in 5 fields for 3 independent experiments. ***p < 0.001. Statistical analysis is performed using one-way ANOVA followed by Tukey post-test. Scale bars, 100 µm.

**Supplementary Figure 2:** (**A**) Schematic representation of the SNCA gene locus**.** (**B**) Tide analysis and Sanger sequencing of iPSCs line with 4x, 3x, 2x and 1x*SNCA* copies. (**C**) Schematic representation of two sgRNAs on exon 3 and exon 4 used to obtain the *SNCA*-KO iPSCs. (**D**) Image of PCR amplication used to select clone with deletion. (**E**) Tide analysis and Sanger sequencing confirms the deletion in the *SNCA*-KO iPSCs.

**Supplementary Figure 3:** (**A**) Differentiation protocol to generate DA neuronal cultures. (**B**) Representative images and quantification of regionalized midbrain NPCs stained for Nestin (red) and FOXA2 (green) in 4x, 3x, 2x, 1x*SNCA* and *SNCA*-KO midbrain NPCs. Values are mean ± SEM in 3 fields for 3 biological independent experiments. **p < 0.01, ***p < 0.001. Statistical analysis is performed using one-way ANOVA followed by Tukey post-test. Scale bars, 100 µm.

**Supplementary Figure 4:** Representative images of SNCA-KO**,** 2x and 4x*SNCA* neurons infected with the LV:Syn-GFP and stained for GFP and the pan-neuronal marker MAP2. Scale bars, 100 µm.

**Supplementary Figure 5:** Representative images and quantification of the FluoReSyn signal in 4x, 3x, 2x, 1x*SNCA* and *SNCA*-KO iPSC-derived DA neurons. Values are mean ± SEM. Dots represent quantification in 5 fields for 4 independent experiments. ***p < 0.001. Statistical analysis is performed using one-way ANOVA followed by Tukey post-test. Scale bars, 100 µm.

**Supplementary Figure 6:** Representative images and quantification of the Thioflavin S signal in 3 weeks old 4x, 3x, 2x, 1x*SNCA* and *SNCA*-KO DA neurons. Values are mean ± SEM of 4 biological independent experiments. ***p < 0.001. Statistical analysis is performed using one-way ANOVA followed by Tukey post-test. Scale bars, 100 µm.

**Supplementary Figure 7:** Representative images and quantification of 4x, 3x, 2x, 1x*SNCA* and *SNCA*-KO DA neurons co-stained for pS129αSyn and TH at 2, 3, 4 and 5 weeks of in vitro neuronal differentiation. Values are mean ± SEM. Dots indicate quantifications in 5 fields for 4 biological independent experiments. ***p < 0.001. Statistical analysis is performed using one-way ANOVA followed by Tukey post-test. Scale bars, 100 µm.

**Supplementary Figure 8:** Representative images of 4x, 3x, 2x, 1x*SNCA* and *SNCA*-KO mDA neurons immunostained for pS129αSyn (green) and total αSyn (red) showing the similar increase over time and relative co-distribution in neuronal cells (n=3 independent biological experiments). Scale bars, 100 µm.

**Supplementary Figure 9:** Representative images of 4x, 2x*SNCA* and *SNCA*-KO DA neurons immunostained for FluoReSyn-GFP signal (green) and pS129αSyn (red) to highlight their co-distribution in DA neuronal cultures. Signal corresponding to one αSyn inclusion is shown in the insets (n = 3 biological experiments). Scale bar, 100 µm.

**Supplementary Figure 10:** Super-resolved images showing the distribution of αSyn puncta and ATP5 positive mitochondrial figures and their relative proximity in 5 week old 4x*SNCA* DA neurons. Scale bars, 100 µm.

**Supplementary Figure 11:**(**A**) Representative images and quantification of the co-colocalization between mitochondria (Mitotracker green) and lysosomes (Lysotracker red) in 2x and 4x*SNCA* DA neurons. Dots indicate the number of cells analyzed (n = 30 somata). (**B**)  Representative images and quantification of the LC3-GFP signal in 2x, 4x*SNCA* and *SNCA*-KO DA neurons. Dots indicate quantifications in 5 fields for 3 independent experiments.  (**C**) Immunoblot for detecting LC3-I and LC3-II forms in 2x, 4x*SNCA* and *SNCA*-KO DA neuronal cultures. Values are mean ± SEM. ***p < 0.001. Statistical analysis is performed using one-way ANOVA followed by Tukey post-test. Scale bars, 100 µm.

**Supplementary Figure 12:** (**A**)Representative images of CLEM analysis in 4x*SNCA* DA neurons. Red arrows indicate the border of the αSYN inclusion. Green and light blue asterisks indicate abnormal mitochondria and autophagosomes, respectively. (**B**) Western blot analysis shows insoluble αSYN signal in 2 week old 4x*SNCA* treated with αSYN fibrils. (n = 3 independent experiments).

**Supplementary Figure 13:** (**A**) Schematic representation of TAXBP1 gene locus. (**B-C**) Sanger sequencing and Tide analysis confirm the nucleotide changes in the 4x*SNCA*;TAXBP1-KO iPSCs. (**D**) iPSC colonies immunostained for the pluripotency markers Oct4, NANOG and their morphology appearance of the 4x*SNCA*;TAXBP1-KO iPSC line. Scale bars, 100 µm.

**References**

1. Blauwendraat C, Nalls MA, Singleton AB. [The genetic architecture of Parkinson's disease.](https://pubmed.ncbi.nlm.nih.gov/31521533/) *Lancet Neurol.* **19**, 170-178 (2020).
2. Goedert M, Spillantini MG, Del Tredici K, Braak H. 100 years of Lewy pathology. *Nat Rev Neurol.* **9**, 13-24 (2013).
3. Vila M, Przedborski S. Genetic clues to the pathogenesis of Parkinson's disease. *Nat Med.* **10**, S58-62 (2004).
4. Polymeropoulos MH, Lavedan C, Leroy E, Ide SE, Dehejia A, Dutra A, Pike B, et al. Mutation in the alpha-synuclein gene identified in families with Parkinson's disease. *Science* **276**, 2045-2047 (1997).
5. Spillantini MG, Schmidt ML, Lee VM, Trojanowski JQ, Jakes R, Goedert M. [Alpha-synuclein in Lewy bodies.](https://pubmed.ncbi.nlm.nih.gov/9278044/) *Nature* **388**, 839-840 (1997).
6. Ibáñez P, Bonnet AM, Débarges B, Lohmann E, Tison F, Pollak P, et al. Causal relation between alpha-synuclein gene duplication and familial Parkinson's disease. *Lancet* **364**, 1169-1171 (2004).
7. Singleton AB, Farrer M, Johnson J, Singleton A, Hague S, Kachergus J, et al. alpha-Synuclein locus triplication causes Parkinson's disease. *Science* 302, 841 (2003).
8. Book A, Guella I, Candido T, Brice A, Hattori N, Jeon B, et al. A Meta-Analysis of α-Synuclein Multiplication in Familial Parkinsonism. *Front Neurol.* **9**:1021 (2018).
9. Ibáñez P, Lesage S, Janin S, Lohmann E, Durif F, Destée A, et al. Alpha-synuclein gene rearrangements in dominantly inherited parkinsonism: frequency, phenotype, and mechanisms. *Arch Neurol.* **66**, 102-108 (2009).
10. Guo Y, Sun Y, Song Z, Zheng W, Xiong W, Yang Y, et al. Genetic Analysis and Literature Review of SNCA Variants in Parkinson's Disease. *Front Aging Neurosci.* **13**:648151 (2021).
11. Tanudjojo B, Shaikh SS, Fenyi A, Bousset L, Agarwal D, Marsh J, et al. Phenotypic manifestation of alpha-synuclein strains derived from Parkinson's disease and multiple system atrophy in human dopaminergic neurons. *Nat Commun.* 12:3817 (2021).
12. Angelova PR, Choi ML, Berezhnov AV, Horrocks MH, Hughes CD, De S, et al. Alpha synuclein aggregation drives ferroptosis: an interplay of iron, calcium and lipid peroxidation. *Cell Death Differ.* **27**, 2781-2796 (2020).
13. Zambon F, Cherubini M, Fernandes HJR, Lang C, Ryan BJ, Volpato V, et al. [Cellular alpha-synuclein pathology is associated with bioenergetic dysfunction in Parkinson's iPSC-derived dopamine neurons.](https://pubmed.ncbi.nlm.nih.gov/30753527/) *Hum Mol Genet.* **28**, 2001-2013 (2019).
14. Burbulla LF, Song P, Mazzulli JR, Zampese E, Wong YC, Jeon S, et al. Dopamine oxidation mediates mitochondrial and lysosomal dysfunction in Parkinson's disease. *Science* 357, 1255-1261 (2017).
15. Heman-Ackah SM, Manzano R, Hoozemans JJM, Scheper W, Flynn R, Haerty W, et al. Alpha-synuclein induces the unfolded protein response in Parkinson's disease SNCA triplication iPSC-derived neurons. *Hum Mol Genet.* **26**, 4441-4450 (2017).
16. Devine MJ, Ryten M, Vodicka P, Thomson AJ, Burdon T, Houlden H, et al. Parkinson's disease induced pluripotent stem cells with triplication of the alpha-synuclein locus. *Nat Commun.* **2**:440 (2011).
17. Byers B, Cord B, Nguyen HN, Schüle B, Fenno L, Lee PC, et al. SNCA triplication Parkinson's patient's iPSC-derived DA neurons accumulate α-synuclein and are susceptible to oxidative stress. *PLoS One* **6**:e26159 (2011).
18. Haenseler W, Zambon F, Lee H, Vowles J, Rinaldi F, Duggal G, et al. Excess α-synuclein compromises phagocytosis in iPSC-derived macrophages. *Sci Rep.* **7**:9003 (2017).
19. Ferese R, Modugno N, Campopiano R, Santilli M, Zampatti S, Giardina E, et l. Four Copies of SNCA Responsible for Autosomal Dominant Parkinson's Disease in Two Italian Siblings. *Parkinsons Dis.* **2015**:546462 (2015).
20. Olgiati S, Thomas A, Quadri M, Breedveld GJ, Graafland J, Eussen H, et al. Early-onset parkinsonism caused by alpha-synuclein gene triplication: Clinical and genetic findings in a novel family. *Parkinsonism Relat Disord.* **21**, 981-986 (2015).
21. Iannielli A, Bido S, Folladori L, Segnali A, Cancellieri C, Maresca A, et al. Pharmacological Inhibition of Necroptosis Protects from Dopaminergic Neuronal Cell Death in Parkinson's Disease Models. *Cell Rep.* **22**, 2066-2079 (2018).
22. Gerdes C, Waal N, Offner T, Fornasiero EF, Wender N, Verbarg H, et al. A nanobody-based fluorescent reporter reveals human alpha-synuclein in the cell cytosol. *Nat Commun.* **11**:2729 (2020).
23. Ludtmann MHR, Angelova PR, Horrocks MH, Choi ML, Rodrigues M, Baev AY, et al. α-synuclein oligomers interact with ATP synthase and open the permeability transition pore in Parkinson's disease. *Nat Commun.* 9:2293 (2018).
24. Wong YC, Krainc D. α-synuclein toxicity in neurodegeneration: mechanism and therapeutic strategies. *Nat Med.* **23**, 1-13 (2017).
25. Zhao YG, Codogno P, Zhang H. Machinery, regulation and pathophysiological implications of autophagosome maturation. *Nat Rev Mol Cell Biol.* **22**, 733-750 (2021).
26. Zucca FA, Segura-Aguilar J, Ferrari E, Muñoz P, Paris I, Sulzer D, et al. Interactions of iron, dopamine and neuromelanin pathways in brain aging and Parkinson's disease. *Prog Neurobiol.* **155**, 96-119 (2017).
27. Meiser J, Weindl D, Hiller K. Complexity of dopamine metabolism. *Cell Commun Signal.* **11**:34 (2013).
28. Shahmoradian SH, Lewis AJ, Genoud C, Hench J, Moors TE, Navarro PP, et al. Lewy pathology in Parkinson's disease consists of crowded organelles and lipid membranes. *Nat Neurosci.* **22**, 1099-1109 (2019).
29. Sarraf SA, Shah HV, Kanfer G, Pickrell AM, Holtzclaw LA, Ward ME, et al. Loss of TAX1BP1-Directed Autophagy Results in Protein Aggregate Accumulation in the Brain. *Mol Cell.* 80, 779-795.e10 (2020).
30. Oliveira LM, Falomir-Lockhart LJ, Botelho MG, Lin KH, Wales P, Koch JC, et al. Elevated α-synuclein caused by SNCA gene triplication impairs neuronal differentiation and maturation in Parkinson's patient-derived induced pluripotent stem cells. *Cell Death Dis.* 6:e1994 (2015).
31. Bernardi P, Carraro M, Lippe G. The mitochondrial permeability transition: Recent progress and open questions. *FEBS J.* 2021 Oct 28. Online ahead of print.
32. Bauer TM, Murphy E. Role of Mitochondrial Calcium and the Permeability Transition Pore in Regulating Cell Death. *Circ Res*. **126**, 280-293 (2020).
33. Mahul-Mellier AL, Burtscher J, Maharjan N, Weerens L, Croisier M, Kuttler F, et al. The process of Lewy body formation, rather than simply alpha-synucleinfibrillization, is one of the major drivers of neurodegeneration. *Proc Natl Acad Sci U S A.* **117**, 4971-4982 (2020).
34. Concordet JP, Haeussler M. CRISPOR: intuitive guide selection for CRISPR/Cas9 genome editing experiments and screens. *Nucleic Acids Res.* **46**, W242-W245 (2018).
35. Giannelli SG, Luoni M, Castoldi V, Massimino L, Cabassi T, Angeloni D, et al. Cas9/sgRNA selective targeting of the P23H Rhodopsin mutant allele for treating retinitis pigmentosa by intravitreal AAV9.PHP.B-based delivery. *Hum Mol Genet.* 27, 761-779 (2018).
36. Marchetto MC, Carromeu C, Acab A, Yu D, Yeo GW, Mu Y, et al. A model for neural development and treatment of Rett syndrome using human induced pluripotent stem cells. *Cell* **143**, 527-39 (2010).
37. Kriks S, Shim JW, Piao J, Ganat YM, Wakeman DR, Xie Z, et al. Dopamine neurons derived from human ES cells efficiently engraft in animal models of Parkinson's disease. *Nature* 480, 547-51 (2011).
38. Kirkeby A, Grealish S, Wolf DA, Nelander J, Wood J, Lundblad M, et al. Generation of regionally specified neural progenitors and functional neurons from human embryonic stem cells under defined conditions. *Cell Rep*. **1**, 703-14 (2012).
39. Ghee M, Melki R, Michot N, Mallet J. PA700, the regulatory complex of the 26S proteasome, interferes with alpha-synuclein assembly. *FEBS J.* **272**, 4023-4033 (2005).
40. Shrivastava AN, Bousset L, Renner M, Redeker V, Savistchenko J, Triller A et al. [Differential Membrane Binding and Seeding of Distinct α-Synuclein Fibrillar Polymorphs.](https://pubmed.ncbi.nlm.nih.gov/32059758/) *Biophys J.* **118**, 1301-1320 (2020).
41. Rey NL, Bousset L, George S, Madaj Z, Meyerdirk L, Schulz E, et al. α-Synuclein conformational strains spread, seed and target neuronal cells differentially after injection into the olfactory bulb. *Acta Neuropathol Commun.* **7**:221 (2019).
42. Makky A, Bousset L, Polesel-Maris J, Melki R. Nanomechanical properties of distinct fibrillar polymorphs of the protein α-synuclein. *Sci Rep.* **6**:37970 (2016).
43. Grozdanov V, Bousset L, Hoffmeister M, Bliederhaeuser C, Meier C, Madiona K, et al. Increased Immune Activation by Pathologic α-Synuclein in Parkinson's Disease. *Ann Neurol.* **86**, 593-606 (2019).
44. Peelaerts W, Bousset L, Van der Perren A, Moskalyuk A, Pulizzi R, Giugliano M, et al. α-Synuclein strains cause distinct synucleinopathies after local and systemic administration. *Nature* **522**, 340-344 (2015).
45. Luk KC, Song C, O'Brien P, Stieber A, Branch JR, Brunden KR, et al. Exogenous alpha-synuclein fibrils seed the formation of Lewy body-like intracellular inclusions in cultured cells. *Proc Natl Acad Sci U S A.* **106**, 20051-20056 (2009).
